# Routemap for health impact assessment implementation: scoping review using the consolidated framework for implementation research

**DOI:** 10.1093/heapro/daaf080

**Published:** 2025-06-30

**Authors:** Tara Kenny, Ben Harris-Roxas, Sheena McHugh, Margaret Douglas, Liz Green, Fiona Haigh, Joanna Purdy, Paul Kavanagh, Monica O’Mullane

**Affiliations:** School of Public Health, University College Cork, 4th Floor, Western Gateway Building, Western Road, Cork T12 XF62, Ireland; School of Population Health, University of New South Wales, UNSW Sydney 2052, Australia; School of Public Health, University College Cork, 4th Floor, Western Gateway Building, Western Road, Cork T12 XF62, Ireland; Public Health Scotland, Gyle Square, 1 South Gyle Crescent, Edinburgh EH12 9EB, United Kingdom; School of Health and Wellbeing, University of Glasgow, 90 Byres Road, Glasgow G12 8TB, United Kingdom; International Health, WHO Collaborating Centre on ‘Investment in Health and Well-Being’, Public Health Wales, Number 2 Capital Quarter, Tyndall Street, Cardiff CF104BZ, United Kingdom; Department of International Health, Care and Public Health Research Institute—CAPHRI, Maastricht University, PO Box 616 6200 MD, Maastricht, The Netherlands; International Centre for Future Health Systems, University of New South Wales, UNSW Sydney 2052, Australia; Sydney Local Health District, PO Box M30, Missenden Road, Camperdown NSW, Sydney 2050, Australia; Institute of Public Health, 700 South Circular Road, Dublin 8 D08 NH90, Ireland; National Health Intelligence Unit, 4th Floor Jervis House, Jervis Street, Dublin DO1 E3W9, Ireland; School of Public Health, University College Cork, 4th Floor, Western Gateway Building, Western Road, Cork T12 XF62, Ireland

**Keywords:** health impact assessment (HIA), implementation science, consolidated framework for implementation science research (CFIR), health in all policies, scoping review

## Abstract

Health Impact Assessment (HIA) provides a practical set of tools to appraise the potential health effects of a policy, programme, or project prior to implementation. HIA has gained significant attention in recent decades due to its utility in facilitating a broader understanding of health and bringing diverse stakeholders and evidence into decision-making processes. Despite this interest in HIA its implementation remains challenging within governance, decision making, and regulatory contexts. The Consolidated Framework for Implementation Research (CFIR) 2.0 provides a methodological framework to identify potential factors influencing implementation and the domains in which they operate, within the framework. For the purpose of this scoping review, implementation refers to the process of carrying out an HIA, and where applicable, the implementation of its recommendations. This review presents a novel exploration of HIA from an implementation science perspective. It provides a synthesis of the factors influencing HIA implementation and identifies a range of considerations and strategies that may facilitate and strengthen HIA implementation and support. The findings suggest that the earlier steps are critical in assisting the practical application and implementation of HIA. However, building wider HIA support, awareness, and capacity essential to progressing HIA is dependent on wider public health advocacy and addressing challenges specific to HIA as a method and tool. CFIR offers a useful and adaptable framework that could be used for supporting HIA planning, practice, and implementation.

Contribution to Health Promotion

- HIA is a core component of the health promotion practise.
- CFIR provides a useful framework that could be used for supporting HIA planning, practice and implementation.
- The early planning steps of HIA are critical in assisting the practical application and implementation of HIA.
- HIA implementation that encapsulates HIAs core values of equity and participation require attention at the earlier stages of the HIA and may be difficult to retrofit post scoping.
- Building wider HIA support, awareness, and capacity essential to progressing HIA is dependent on wider public health advocacy and addressing challenges specific to HIA as a method and tool.

## BACKGROUND

Health Impact Assessment (HIA) is described as a ‘combination of procedures, methods, and tools’ (WHO 1999) used to systematically appraise the potential health effects of policy, plan, project, programme, or intervention is a core component of health promotion practise (Harris-Roxas and O’Mullane 2017). The Ottawa Charter identifies building healthy public policy, strengthening community action, and creating supporting environments as key action areas within health promotion (International Union for Health Promotion and Education 2017). HIA assists in translating the principles of the Ottawa Charter into practice by advocating for consideration of health and social determinants of health in policies, plans and projects across sectors (Kemm 2001; Gulis 2019). In 2010, the World Health Organization (WHO) Commission on the Social Determinants of health (CSDH) revived the WHO constitutional commitments to health equity described as ‘the absence of unfair and avoidable or remediable differences in health amongst social groups’ and called explicitly for ‘health equity impact assessments (IAs) of all economic agreements, market regulation and public policies’ (Solar and Irwin 2010 , 14). Through the assessment of the distribution of potential impacts across population groups, HIA identifies ways to mitigate negative health impacts and enhance positive impacts, ultimately contributing to improving health equity and reducing health inequalities (Wismar *et al*. 2007; Lynch *et al*. 2024).

HIA guidance generally includes five steps: screening, scoping, appraisal, reporting, and monitoring activities. Screening involves assessing whether a HIA is needed or not. Scoping is where the planning of the HIA commences with the identifying potential health risks and benefits, establishing steering/advisory/working groups/committees along with developing and adopting the terms of reference, boundaries for the HIA and the group(s) responsible for its implementation. This is followed by appraisal stage described as the ‘the core’ of HIA. Here, data and evidence are gathered and analysed, affected populations and impact estimates are identified, which lead to recommendations for actions to mitigate negative impacts and promote opportunities to elevate positive impacts. This is followed by reporting where results are reported along with overall findings and recommendations. The final step is monitoring to evaluate the process and uptake of recommendations (WHO n.d.). Whilst there is consensus around these basic HIA steps, there is considerable variation in both methods and application of HIAs’ guiding principles (Kim *et al*. 2024; McDermott *et al*. 2024).

Building on the HIA principles of democracy, equity, sustainable development, and ethical use of evidence outlined in the Gothenburg consensus paper (WHO 1999) the International Association for IA expanded these values to define five core guiding principles for HIA. These principles are Equity and Equality, Participation, Ethical use of evidence, Sustainability, and a Comprehensive approach to health. These overarching principles apply to each step in the HIA process and recognize the inter-sectional responsibility for population health (Winkler *et al*. 2021). Recognizing the utility of HIA as a means to protect population health, several countries have already institutionalized HIA within national laws and constitutions (Osofsky and Pongsiri 2018; Kalel *et al*. 2023) or are in the process of doing so (Welsh Government 2017).

Despite HIAs’ continued growth and importance as a tool and practice that can facilitate cross sectoral collaboration (Dills *et al*. 2022), a Health in All Policies (HiAP) approach (Osofsky and Pongsiri 2018; Greer *et al*. 2022) and support for a transition to a well-being economy aligned with human and planetary health (Douglas *et al*. 2024), its implementation remains *ad hoc* (O’Mullane *et al*. 2024). Common barriers and criticisms of HIA affecting its uptake often stem from its lack of standardized practise, limited skills, and resources for conducting HIA, methodological challenges, inadequate understanding of HIA amongst decision makers and public health professionals, and the lack of polices and legislative frameworks supporting its use (Winkler *et al*. 2020; Liu *et al*. 2023; Kumar and Rasania 2024). There is a need to improve HIA practice and theory to realize the wide-ranging benefits of HIA (Kemm 2005; Kim *et al*. 2024).

Implementation science (IS) emerged to reduce the gap between research and practice as a means to improving population health outcomes (Kilbourne *et al*. 2020). Characterized by a series of research designs and methodological approaches, IS draws from several disciplines including public health, organizational and social psychology, behavioural economics, and engineering (Lane-Fall *et al*. 2019). Its purpose is to identify barriers and facilitators influencing the uptake of evidence-based practise and policy and to produce evidence for implementation strategies and evidence on the effectiveness of strategies (Eccles and Mittman 2006; Powell *et al*. 2017). Theoretical frameworks are widely used in IS to strengthen understanding of the barriers and enablers of implementation.

The Consolidated Framework for Implementation Research (CFIR) (Damschroder *et al*. 2009, 2022) is concerned with factors affecting the implementation of ‘innovations’, the ‘thing being implemented’, which can be a programme, policy, or in this case a process that is recommended for routine practise. CFIR is amongst the most frequently used descriptive framework that lists key implementation determinants across five domains: (i) the innovation, (ii) the outer setting, (iii) the inner setting, (iv) the individual, and (v) the process (Damschroder *et al*. 2009, 2022). Using the example of HIA, domain one captures the characteristics of HIA as a tool or method. Domain two represents the outer setting, referring to the wider economic, political, and social context. This may include the local or national contextual factors such as external policies that may influence the working of the organizations involved in implementing HIA. Domain three refers to the inner setting context, such as the organizational context of those involved in implementing the HIA. Domain four is the individual domain and captures the beliefs, roles, and characteristics of those involved implementing HIA. Domain five captures the process of implementing and refers to the strategies and activities, such as planning and assessing needs used to implement HIA. Each domain is mapped to several constructs and sub-constructs built upon existing implementation theories and conceptual models (ibid).

To the best of our knowledge, HIA has not been explored from an IS perspective and here we propose CFIR as a conceptual framework to identify where potential implementation challenges and opportunities exist. Whilst there are weaknesses associated with assuming a ‘barrier and enabler’ approach to complex challenges, such as the failure to consider the level at which these barriers and enablers exist, using theories and frameworks to guide such research facilitates attention to different levels and domains of influence (Haynes and Loblay 2024). Differentiating these domains of potential influence and interpreting the inter-action between these domains may be useful for HIA practitioners and advocates and in developing strategies to address potential barriers and improve HIA practice more generally. Given HIAs recognition as a core component of health promotion (Harris-Roxas and O’Mullane 2017) and its ability to progress a HiAP approach (Osofsky and Pongsiri 2018; Greer *et al*. 2022) this research will also be of value to public health professionals more broadly.

The aim of this scoping review is to explore and synthesize the factors influencing HIA implementation using CFIR and to identify considerations and potential strategies which may contribute towards addressing the challenges raised. Accordingly, the objectives of this scoping review are three-fold. First, to identify and map the peer reviewed evidence concerning HIA implementation. Secondly, to employ CFIR to synthesize the barriers and enablers influencing HIA implementation and to illustrate the domain in which these influences operate. And thirdly, to identify considerations and potential strategies which may address the primary barriers raised. For the purpose of this CFIR informed scoping review, implementation refers to the process of carrying out the HIA steps and, where applicable, the implementation of its recommendations. Using CFIR to explore HIA implementation offers an approach that may improve practice as well as informing research on the use and adoption of HIA.

## METHODS

### Search methods and study selection

Given the exploratory nature of this research, a scoping review was chosen as the most suitable research method to achieve the objectives (Arksey and O’Malley 2005; Mak and Thomas 2022). Using the key search term ‘Health Impact Assessment’ AND ‘HIA’ AND ‘Implement*’ a search for English-language articles published in peer-reviewed journals was conducted from 1st January 2013 and 31st January 2025, across three electronic databases: PubMed, Web of Science, and Scopus. Peer reviewed journal articles that focussed on HIA implementation and that provided information relating to the process of implementation and/or factors influencing HIA implementation process was included. Eligible articles focussed on the experiences of (i) those involved in HIA or those likely to be involved in future HIA implementation or (ii) by examining individual HIA reports or case studies were included. Articles where HIA was part of another assessment were excluded (Supplementary File S1).

### Data extraction and analysis

Included articles were uploaded to NVivo 14. Metadata were extracted on title, authors, publication year, research question/objectives, country of study, methods, study participants, and, where applicable, number of HIA reports and case studies included each of the articles (Supplementary File S2). Considering the variance in study type and methods of data collection, each article was subsequently categorized and analysed into three groups of papers. Group 1 papers were those that collected primary data from HIA practitioners, participants, and experts directly involved in, or likely to involved in HIA implementation efforts (*n* = 27). Group 2 papers (*n* = 8) were articles based on secondary analysis of HIA case study reports, HIA programmes and HIA review papers (Supplementary File S3 and Tables S1 and S2). Group 3 papers are standalone HIA case studies describing the implementation process (*n* = 10). A second and separate data extraction process was applied to these case studies. Data concerning the authors, country, HIA type (Harris-Roxas and Harris 2011), focus, timeframe in which the HIA was completed, HIA objectives, stakeholders involved, evidence used, initiating party, and the stages of HIA completed, was extracted and tabled (Supplementary File S3 and Table S3).

### Operationalizing CFIR

CFIR 2.0 is intended to be used ‘to collect data from individuals’ involved in the implementation of an innovation (Damschroder *et al*. 2022 :5). This rendered some of the constructs in the individual domain, such as those related to the individuals motivation and commitment, less suitable for secondary analysis in the form of reviews when the included studies did not apply CFIR in the first instance. Consequently, this review attempted to apply CFIR 2.0 (ibid) with the addition of ‘knowledge and beliefs’ and ‘self-efficacy’ constructs within the individual domain from the original CFIR (Damschroder *et al*. 2009), which were more appropriate to the information and detail provided within the included studies.

The application of CFIR required an iterative and staged process carried out by two authors (TK & M’OM) in consultation with the co-authors. The initial phase involved first, inductively coding factors influencing HIA implementation identified in the results and discussion of each paper as a barrier or facilitator (e.g. assigning ‘lack of accepted screening and evaluation tools leading to subjective judgement’ as a barrier). Second, deductively assigning the barrier or facilitator to one of the five CFIR domains (e.g. classifying this barrier as a challenge related to HIA as a tool and method in the innovation domain). And thirdly, guided by definitions provided in the original and updated CFIR (Damschroder *et al*. 2009, 2022) assigning a CFIR construct and, where applicable, sub-construct best reflected in the data segment (e.g. assigning this innovation domain barrier within the ‘evidence base’ construct). Supplementary File S4 provides an overview of the application of CFIR to the barriers and facilitators identified within the literature and disaggregated by each group of papers.

Building on the first phase of analysis, the second phase focussed on identifying considerations and potential measures, which may assist in addressing common HIA barriers. This process began by first organizing facilitators located within individual and process domains, domains in which those directly involved in implementation HIA may have more control over in comparison to other CFIR domains such as inner and outer setting domains. We then aligned these considerations with specific steps in HIA in order to create a route map of measures which, depending on the context, may improve HIA practice (Table 1, results section). Lastly, HIA facilitators operating within the innovation, inner and outer domain were collated to identify strategies and opportunities aimed at addressing HIA capacity, awareness and support barriers (Table 2, results section).

**Table 1.** Individual and process domain measures to improve HIA implementation and aligned with the steps of HIA.

Potential measures located with the individual and process domains to improve HIA implementation					
Measures that may support HIA implementation	HIA stage where consideration is required	Concerned with:	CFIR Domain	CFIR Construct: sub-constructs (where applicable)	Source
Mapping potential stakeholders before HIA commences to facilitate diverse representation	Before HIA commences	Stakeholders	Process	Doing	Negev *et al*. (2013)
Adapting HIA to organization and legislative context and political and-administrative context including adapting language to municipal realties	All steps	Adapting to context	Process	Adapting	Haigh *et al*. (2015), Gamache *et al*. (2020), Jabot and Rivadeneyra-Sicilia (2022), Negev *et al*. (2013)
Establishing good communication channels throughout HIA, i.e. improving reporting and presenting skills of those involved in HIA	All steps	Developing HIA team skills/building capacity	Individual	Implementation facilitators	Kraemer *et al*. (2014)
Providing greater transparency re. decision making, potential vested interests, accountability and recommendations.	All steps	Transparency	Process	Doing	Busato and Grisotti (2022), Morteruel *et al*. (2020), Gamache *et al*. (2022), Linzalone *et al*. (2018), Kraemer and Gulis (2014), Berensson and Tillgren (2017), Linzalone *et al*. (2017)
Enhancing and strengthening assessment quality and rigour to build trust in HIA	All steps	Building trust amongst stakeholder	Process	Doing	Liu *et al*. (2023), Marincova *et al*. (2020)
Facilitating participation of affected populations at all steps	All steps	Equity	Process	Engaging recipients	Gamache *et al*. (2022), Roué-Le Gall and Jabot (2017); Fischer Chang and Muthoora (2024)
Considering if momentum is growing for a particular issue, if decision makers have basic knowledge about health relate issues, what connections exist between those conducting the HIA and decision makers, and how the timing of the HIA fits with the decision-making process	Before HIA commences	Paying attention to the context of the proposed HIA	Process	Assessing context	Bourcier *et al*. (2015)
Having decision makers on the HIA team/‘High level involvement’	Before HIA commences	HIA team and key stakeholder	Individual	High-level leaders	Bourcier *et al*. (2015), Harris-Roxas *et al*. (2014), Haigh *et al*. (2015)
Having the HIA led by an independent person with no political constraints, i.e. an academic without responsibility for health or urban planning in the context of an HIA on an urban planning project	Before HIA commences	Obtaining buy-in and support for the HIA	Individual	Role: implementation leads	Gamache *et al*. (2020)
Having people experienced in carrying out HIA with confidence in HIA methodology on the HIA committee/working group	Before HIA commences	Skills required to implement HIA	Individual	Implementation Facilitators/leads	Ison (2013), Jabot *et al*. (2020)
Involving people with knowledge, and access to decision making processes, and also people with relevant skills as early as possible	Before HIA commences	HIA team/Key stakeholders	Individual	Implementation facilitators/lead	Haigh *et al*. (2015), Linzalone *et al*. (2017)
Ensuring diverse, inter-disciplinary, multi-cultural, multi-disciplinary HIA teams with a range of competencies, skills and expert knowledge	Before HIA commences	HIA teams	Individual	Implementation leads/facilitators	Bourcier *et al*. (2015), Busato and Grisotti (2022), Haigh *et al*. (2015), Jabot *et al*. (2020), Morteruel *et al*. (2020), Negev *et al*. (2013), Green *et al*. (2020a)
Designing steering/advisory committee/groups to maximize diverse inter-actions	Before scoping stage	Planning	Process	Doing	Negev *et al*. (2013)
Considering Points of influence: Paying attention to national policymaking and planning systems and considering broad contextual factors such as political contexts: recognizing policy windows—opportunities. Focussing HIA on broad societal concerns to encourage social acceptance and linking actions to outside of the HIA process	Before HIA commences, screening, Scoping, appraisal and reporting	Wider policy context	Process	Assessing context	Damari *et al*. (2018), Fakhri and Harris (2021), Haigh *et al*. (2015), Kraemer *et al*. (2014), Gamache *et al*. (2020)
Review of literature at screening stage for insight into potential health impacts	Screening and scoping	Assessment	Process	Doing	Sheffield *et al*. (2014)
Clear identification of roles and actors responsible for activities within different HIA phases	Before scoping and throughout all steps	Clear identification of roles	Process	Planning	Ramirez-Rubio *et al*. (2019)
Tailoring training to the stakeholder role in the HIA process (i.e. people outside Public Health Sector)	Before scoping begins	Building capacity	Process	Tailoring strategies	Goff *et al*. (2016)
Contextualizing data (local data with data from other comparable setting)	Screening and scoping	Data	Process	Doing	Ramirez-Rubio *et al*. (2019)
Setting realistic expectations from the onset	Screening and scoping	Managing expectations	Process	Planning AND Assessing context	Kraemer *et al*. (2014)
Involving private project promoters to show that HIA can improve projects at a low cost and encourage social acceptance	Before HIA commences	Building wider support for HIA	Individual	Implementation facilitators	Gamache *et al*. (2020)
Establishing shared values/culture, explicit goals and clearly defined roles and responsibilities and expected outcomes	Scoping	Clarity in purpose and process	Process	Planning	Haigh *et al*. (2015), Jabot *et al*. (2020), Goff *et al*. (2016), Ramirez-Rubio *et al*. (2019)
Multi-level stakeholder engagement—different levels of decision making	Scoping	Stakeholder engagement	Process	Engaging	Thondoo *et al*. (2020a)
Encouraging understanding and the recognition of common goals and shared interests amongst key agents/stakeholder	Scoping	Building common goals, understanding	process	Engaging	Morteruel *et al*. (2020), Gamache *et al*. (2020)
Highlighting social responsibility of decision makers (long term versus short term vision)	Scoping, appraisal, and reporting	Building support	Process	Engaging	Thondoo *et al*. (2020a)
Public forum at preliminary phase of HIA to build trust amongst stakeholders (political–admin and civil society)	Scoping	Building support, HIA awareness and engaging	Process	Engaging	Linzalone *et al*. (2017)
Meaningfully engaging with and involving key stakeholders: community, decision makers, influential people, experts with specific knowledge	Scoping	Stakeholder engagement	Process	Engaging	Bourcier *et al*. (2015), Gamache *et al*. (2022), Buregeya *et al*. (2020), Goff *et al*. (2016), Haigh *et al*. (2015)
Identifying relevant stakeholders and points of influence within systems and consider how the HIA can affect these	Scoping	Stakeholders	Process	Assessing context	Haigh *et al*. (2015)
Developing methodological solutions to conduct stakeholder consultation (i.e. selecting issues that matter in the scoping stage)	Scoping	Citizen involvement/Equity	Process	Assessing needs	Linzalone *et al*. (2018)
Hosting equality themed workshops with impacted communities and paying people for their time	Screening, scoping, and appraisal	Citizen involvement/equality/equity	Process	Doing	Fischer *et al*. (2024)
Assuming a co-construction approach with citizens potentially impacted by the proposal.	Screening, scoping, appraisal	Moving beyond engagement	Process:	Engaging: innovation deliverers	Roué-Le Gall and Jabot (2017)
Providing space for citizen participation	Screening, scoping and appraisal	Citizen involvement	Process	Engaging: recipients	Morteruel *et al*. (2020), Thondoo *et al*. (2020a)
Building community capacity to engage in HIA process	Screening, scoping and appraisal	Building community capacity	Process	Engaging: recipients	Haigh *et al*. (2015), Pursell and Kearns (2013)
Recognizing and addressing the power discrepancies and cultural and language barriers between service agencies and communities	Scoping, appraisal	Equity	Process	Assessing needs	Pursell and Kearns (2013), Negev *et al*. (2013)
More in-depth consideration and inclusion of different vulnerable groups and of citizens opinions	Scoping, appraisal	Equity	Process	Assessing needs	Busato and Grisotti (2022)
Using locally relevant data and considering the scientific evidence and contextualizing scientific evidence with local evidence	Scoping and appraisal	Assessment	Process	Doing	Bourcier *et al*. (2015), Busato and Grisotti (2022), Morteruel *et al*. (2020)
Incorporating indirect health impacts when assessing impacts	Appraisal	Assessment	Process	Doing	Fakhri *et al*. (2015), Westenhöfer *et al*. (2023)
Paying more attention to the needs of vulnerable populations	Screening, scoping and appraisal	Equity	Process	Assessing needs: recipients	Bourcier *et al*. (2015), Kögel *et al*. (2020)
In view of limited resources, the use of simple tools to bring a health perspective to decisions and the use of pre-existing structures and procedures as an alternative to a full HIA	Scoping and appraisal	Tailoring HIA to available resources	Process	Assessing context	Morteruel *et al*. (2020)
Reporting results concisely and providing a shorter version of the report for stakeholders	Reporting	Accessibility of findings	Process	Doing	Gamache *et al*. (2022)
Encouraging a recognition of the value of lay knowledge and acceptance of this knowledge alongside expert opinion	Scoping and appraisal	Assessment	Process	Doing	Thondoo *et al*. (2020a), Negev *et al*. (2013)
Creating recommendations that are actionable, realistic, and sector-specific that consider the decision makers authority to act, timelines, and potential costs	Reporting and monitoring	Reporting	Process	Doing	Bourcier *et al*. (2015)
Ensuring that the recommendations reflect what the community impacted want	Reporting	Reporting	Process	Doing	Fischer *et al*. (2024)
Increased scrutiny of the monitoring stage to address evidence gap by illustrating health outcomes	Monitoring and evaluating	Addressing evidence gap	Process	Reflecting and evaluating	Fischer *et al*. (2024)
Establishing cross-sectoral collaborative mechanisms to allow review of implementation issues that arise and enhance acknowledgement and trust amongst participants	Monitoring	Addressing and communication implementation issues	Process	Reflecting and evaluating	Liu *et al*. (2023)

**Table 2.** Strategies to build HIA capacity and awareness and support.

Strategies targeting inner, outer, and innovation domain to build HIA capacity and awareness and support				
Strategy	Proposed mechanism	Domain	Construct: sub-construct (where applicable)	Source
Highlight return on investment—cost effectiveness/monetary value and/or the welfare costs of inaction—economic and social	Building awareness of the value of HIA	Innovation	Relative advantage	Mattig *et al*. (2017), Thondoo *et al*. (2020a)
Encouraging belief amongst all stakeholder (government and non-government) that HIA improves rather than hinders development	Building awareness of the value of HIA	Innovation	Relative advantage	Damari *et al*. (2018)
Addressing confusion between HIA and other IA procedures.	Providing clarity of HIA purpose and process/differentiation	Innovation	Relative advantage	Jabot and Rivadeneyra-Sicilia (2022)
Building awareness and understanding of how HIA can contribute to stakeholders agendas and its added value	Building awareness of the value of HIA	Innovation	Relative advantage	Jabot *et al*. (2020), O’Mullane (2014), Liu *et al*. (2023)
Promoting HIA as a tool that can be used to promote HiAP	Promoting/advocating HIA as a HiAP tool	Innovation	Relative advantage	Goff *et al*. (2016), Jabot and Rivadeneyra-Sicilia (2022)
Confronting misunderstandings and creating awareness of scope and effectiveness of HIA	Building awareness of HIA	Innovation	Relative advantage	Mattig *et al*. (2017)
Promoting HIA as a tool that facilitates cross-sectoral relationship, building and improved understanding of the determinants of health	Highlighting indirect benefits of HIA	Innovation	Relative advantage	Haigh *et al*. (2013)
Collaboration with media to introduce and explain concept. Create buy-in and value awareness	Building awareness of HIA	Outer setting	Partnerships and Connections	Marincova *et al*. (2020)
Public health advocacy for HIA and collaboration between regional health institutions and their partners i.e. regional and local authorities and communities.	Promoting collaboration and advocacy	Outer setting	Partnerships and Connections	Roué-Le Gall and Jabot (2017), Bever *et al*. (2021), Kögel *et al*. (2020), Liu *et al*. (2023)
Establishing HIA units within and across countries	Building networks	Outer setting	Partnerships and Connections	Marincova *et al*. (2020), O’Mullane (2014), Walpita and Green (2022)
Encouraging membership with external HIA networks, Health city networks and creating HIA platforms	Building networks	Outer setting	Partnerships and Connections	Goff *et al*. (2016), Kraemer *et al*. (2014), Mattig *et al*. (2017), O’Mullane (2014), Roué-Le Gall and Jabot (2017)
Supporting and promoting inter-sectoral and multi-sectoral work practices and collaboration	Collaboration	Outer setting AND inner setting	Partnerships and Connections AND relational connections	Gamache *et al*. (2020), Haigh *et al*. (2015), Thondoo *et al*. (2020a), Walpita and Green (2022), Kraemer *et al*. (2014), Marincova *et al*. (2020), Liu *et al*. (2023)
HIA dedicated sessions included in national conferences (France) to disseminate knowledge and to encourage the sharing of experiences	Building capacity and awareness of HIA	Outer setting	Local conditions	Jabot and Rivadeneyra-Sicilia (2022)
Involvement of inter-disciplinary students (i.e. public health and urban planners) in HIA in preparation for next generation of urban planners	Building capacity: educational setting	Outer setting	Local conditions: education systems	Gamache *et al*. (2020)
Including public health related teachings such as social medicine and health policy making in the Ministry of Health and Medical Education and promoting inter-disciplinary research	Building capacity: educational setting	Outer setting	Local conditions	Damari *et al*. (2018), Liu *et al*. (2023)
Promoting a broader understanding of health: social model of health	Building awareness of social determinants of health required for HIA	Outer setting	Local conditions	O’Mullane (2014), Linzalone *et al*. (2018)
Training in HIA, including multi-institutional training and training on determinants of health	Training on HIA and Social Determinants of Health	Inner setting AND Outer setting	Available resources: Access to knowledge and information	Ison (2013), Linzalone *et al*. (2018), O’Mullane (2014), Walpita and Green (2022), Jabot and Rivadeneyra-Sicilia (2022), Goff *et al*. (2016), Liu *et al*. (2023)
Access to information and HIA expertize (i.e. national access point to health portal containing a registry of case studies and health data (Linzalone *et al* 2018)	Access to guidance and tools	Inner setting	Available resources: Access to knowledge and information	Ison (2013), O’Mullane (2014), Linzalone *et al*. (2018)
Access to training, workshops and seminars and experience to advance HIA assessment methods, objective analysis, evaluation and discussion by multi-disciplinary experts	Peer learning and critical evaluation and discussion	Inner setting AND Outer setting	Access to knowledge and information	Liu *et al*. (2023)
Development of guidelines and tools, including adaptable and easy to use guidance	Access to guidance and tools	Inner setting AND Outer	Available resources: Access to knowledge and information	Jabot *et al*. (2020), Linzalone *et al*. (2018), Quin *et al*. (2023), Thondoo *et al*. (2020a), Marincova *et al*. (2020), Kraemer *et al*. (2014)
Access to HIA resources in several languages	Accessibility of resources	Inner setting AND Outer	Available resources: Access to knowledge and information	Marincova *et al*. (2020), Kraemer *et al*. (2014), O’Mullane (2014)
Financial support/allocation of funding for HIA	Financial support	Inner setting AND outer setting	Available resources: Funding and financing	Linzalone *et al*. (2018), Fakhri *et al*. (2015), Morteruel *et al*. (2020), Gamache *et al*. (2020), Thondoo *et al*. (2020a), Kögel *et al*. (2020), Liu *et al*. (2023)
Having dedicated HIA staff/posts within organizations	Human resources	Inner setting	Available resources	Ison (2013)
Guidance on how to access necessary data for private proponents	Access to guidance and tools	Inner setting AND outer setting	Available resources: Access to knowledge and information	Linzalone *et al*. (2018)
Creating Supplementary Planning Documents (SPDs) was proposed to help developers understand what they need to do and what the local authority would expect	Access to guidance and tools	Inner setting AND outer setting	Available resources: Access to knowledge and information	Quin *et al*. (2023)
Training for both community and local service agency members engaging in HIA	Access to training	Inner setting	Available resources: Access to knowledge and information	Pursell and Kearns (2013), Negev *et al*. (2013)
Improving technical and governance capacities to enhance the awareness of the potentiality of HIAs	Building capacity	Inner setting	Structural characteristics: governance and communications	Linzalone *et al*. (2018), Liu *et al*. (2023)
Collaboration with an academic/public health institution or local health agency	Collaboration	Inner setting AND outer settings	Partnerships and Connections and relational connections	Ison (2013)
Establishing a partnership between public health actors and municipalities	Collaboration	Inner setting AND outer settings	Partnerships and Connections and relational connections	Jabot *et al*. (2020), Linzalone *et al*. (2018)
Building relationships and understanding between sectors	Collaboration	Inner setting AND outer settings	Partnerships and Connections and relational connections	Morteruel *et al*. (2020), Ison (2013), Liu *et al*. (2023)

The following results section begins with an overview of the included studies before presenting a narrative synthesis of key HIA implementation barriers located across CFIR domains (bolded) and constructs (in italics). Next, HIA implementation facilitators located within process and individual domains are presented and aligned with particular HIA steps to create a practical routemap for those directly involved in implementing HIA. The final section of the results presents a series of strategies, identified from within the literature, that may assist in reducing barriers located within the inner, outer and innovation domain and improve HIA awareness, capacity, and wider support.

## RESULTS

**Figure 1.**
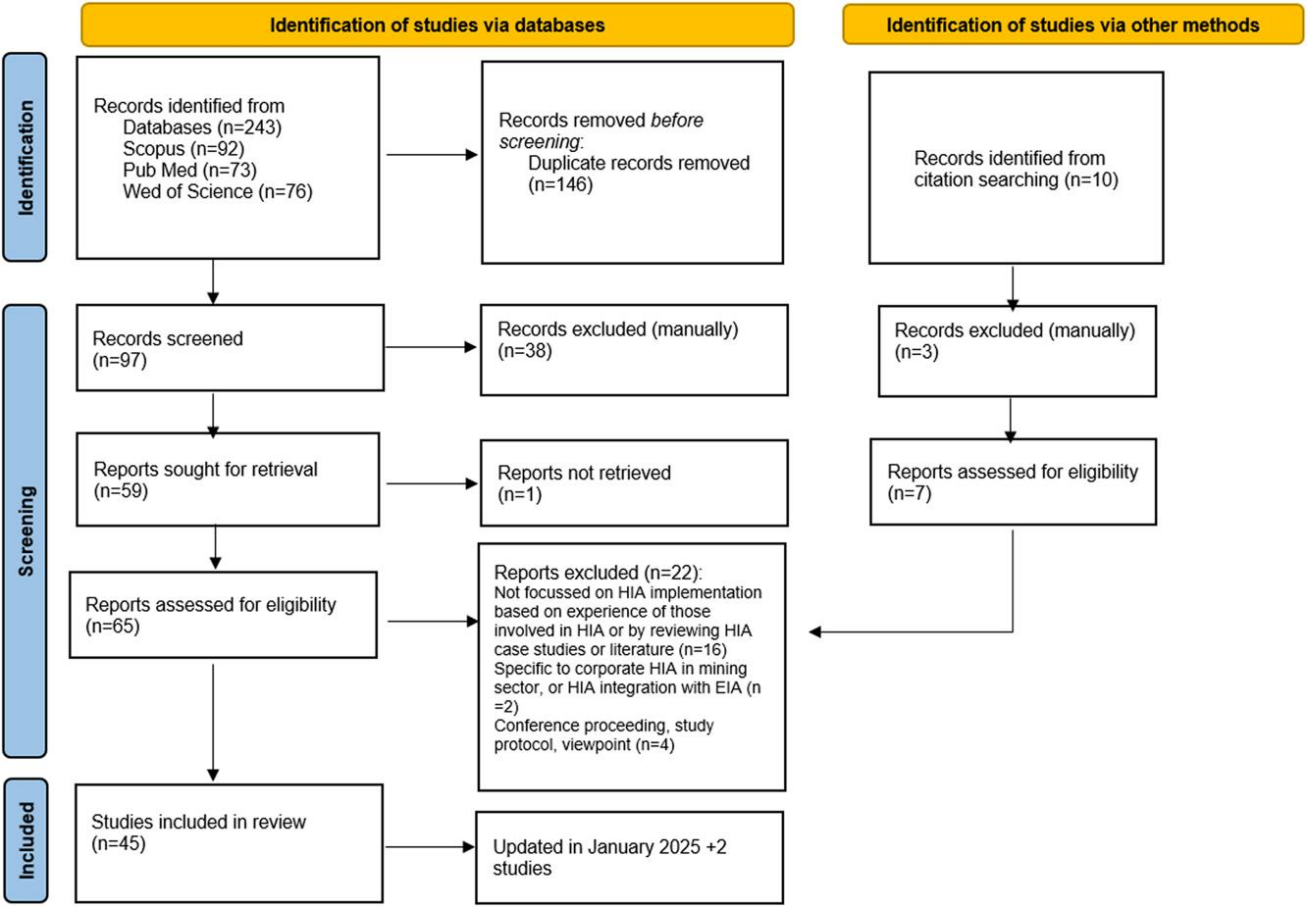
PRISMA flow diagram: literature identification process.

The initial search identified 243 articles (Fig. 1). Records were uploaded to Zotero (Digital Scholarship) and duplicates were removed. A total of 97 abstracts were screened by two researchers (T.K. and M.O.M.) using Rayyan software (Rayyan Systems, Inc., Massachusetts, United States). Conflicts concerning the inclusion of studies were resolved through discussion. 58 papers were retrieved for full text review plus an additional seven identified through citation searching. After reviewing the full text of 65 papers, 43 articles were included. The search was updated in January 2025 and two additional studies were included resulting in 45 articles included in this scoping review.

The majority of articles (*n* = 36/80%) published between January 2013 and January 2025 were based in high-income countries. Most studies (*n* = 32/71%) use qualitative or mixed methods approaches to examine HIA implementation from a range of perspectives. Studies in Group 1 (*n* = 27/60%), based on primary data, are mostly focussed on HIA in one country (*n* = 21/44%) and concentrated on exploring knowledge, attitudes, opinions, and factors inhibiting or facilitating HIA implementation from the perspective of those involved in HIA implementation (Supplementary File S3 and Table S4).

Group 2 studies (*n* = 8/19%) are review papers based on secondary data, such as HIA reports, case studies, focussed on both a single country or multiple countries. These studies are predominately focussed on HIA in an urban environment (Supplementary File S3 and Table S2). Four of these articles are based on a total of 138 HIAs published within the academic literature whilst the remaining four are based on 103 HIA reports from France and the USA.

Group 3 studies (*n* = 10/23%) are standalone case studies presenting and describing the approach taken in implementing a specific strategic or project level HIA (Supplementary File S3). Six case studies were prospective strategic level HIAs and four were prospective project level HIAs. Six were decision support HIAs conducted voluntarily by or with agreement of the organizations responsible for the proposal and four were advocacy HIAs conducted by organizations or groups who are not responsible for the proposal. The time taken to carry out these HIAs, where noted, varied from 6 months to 3 years. Each case study used various forms of stakeholder involvement and public participation was mostly pragmatic and for information gathering purposes. The methods used included surveys (Del Rio *et al*. 2017; Thondoo *et al*. 2020b; Pradyumna *et al*. 2021), discussion groups, focus groups, and workshops (Sheffield *et al*. 2014; Green *et al*. 2020a, 2020b; Kögel *et al*. 2020; Movia *et al*. 2022). This information tended to be gathered at appraisal stage in most of the case studies, or at the screening and scoping stage in two case studies (Del Rio *et al*. 2017; Thondoo *et al*. 2020b). Three HIAs (Negev *et al*. 2013; Sheffield *et al*. 2014; Linzalone *et al*. 2017) involved citizens throughout the HIA implementation process. Further information specific to these HIA case studies can be found in Supplementary File S3.

**Figure 2.**
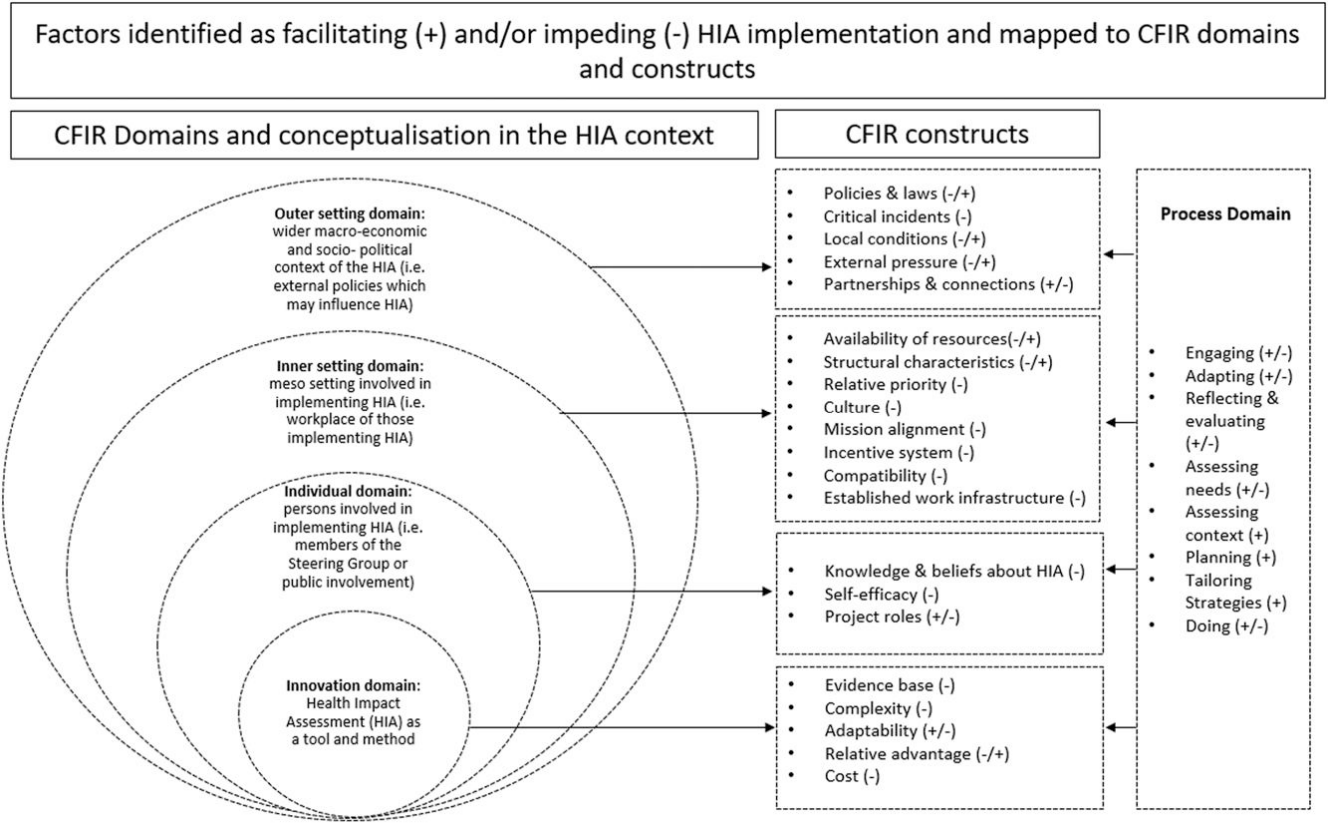
CFIR domains and constructs identified within the literature as facilitating (+) and/or impeding (−) HIA implementation.

As illustrated in Fig. 2, HIA implementation barriers (−) and facilitators (+) are evident across all CFIR domains. The summary table supporting this figure is available in Supplementary File S5.

### HIA implementation challenges across CFIR domains

Within the **‘inner setting domain’,** typically the organizational setting of those involved in implementing HIA, challenges concerning the ‘availability of resources’ to conduct HIA were dominant. This included access to knowledge, information, skills, and training (Negev *et al*. 2013; Harris-Roxas *et al*. 2014; Kraemer and Gulis 2014; Bourcier *et al*. 2015; Linzalone *et al*. 2017; Damari *et al*. 2018; Gamache *et al*. 2020; Jabot *et al*. 2020; Kögel *et al*. 2020; Morteruel *et al*. 2020; Thondoo *et al*. 2020a; Fakhri and Harris 2021; Jabot and Rivadeneyra-Sicilia 2022; Liu *et al*. 2023) along with financial and human resources required to support the assimilation of the recommendations after conducting an HIA (Ison 2013; Kraemer *et al*. 2014; O’Mullane 2014; Buregeya *et al*. 2020; Marincova *et al*. 2020; Morteruel *et al*. 2020; Thondoo *et al*. 2020a; Fakhri and Harris 2021). The ‘relative priority’ afforded to HIA is also a challenge due to perceptions that health is already a consideration across sectors along with competing demands with other health prevention and promotion tasks already being practiced within organizations (Kraemer *et al*. 2014). Or conversely, and related to ‘mission alignment’, that perception of health being outside the remit of some municipal departments (ibid). ‘The structural characteristics’ of the organizations involved in HIA may also make implementing HIA difficult. For example, the dispersion of responsibilities within some organizations may impact the leadership required to implement HIA (Jabot *et al*. 2020). Established working patterns of public administrations within municipalities may also present a challenge (Kraemer *et al*. 2014; Morteruel *et al*. 2020).

In the **‘outer domain’,** which relates to the broader macro environment that may influence the inner domain, challenges within the ‘polices and laws’ construct were prominent and included the lack of policies, laws, regulation, formal agreement, and political support for HIA (Damari *et al*. 2018; Morteruel *et al*. 2020; Thondoo *et al*. 2022; Quin *et al*. 2023). Barriers pertaining to ‘local conditions’ included potential tensions between citizens and those responsible for decision making (Morteruel *et al*. 2020), lack of HIA awareness within educational settings (Marincova *et al*. 2020; Liu *et al*. 2023) and the influence of the private sector in inhibiting formal support for HIA (O’Mullane 2014; Mattig *et al*. 2017). Local conditions may also impact HIAs compatibility with the broader policy and economic context. For example, the potential short term influence of housing focussed HIAs when property ownership and policies change rapidly (Bever *et al*. 2021). An additional challenge identified is HIAs reliance on external ‘partnerships and connections’ to access necessary information. For example, HIAs reliance on policy makers being willing and able to collaborate with relevant institutions to streamline data availability (Ramirez-Rubio *et al*. 2019) required to complete the HIA. However, this challenge may also relate back to the challenge of resources in the inner setting, the time required to build and maintain partnerships and connections and to take part in HIA training (Haigh *et al*. 2015).

Challenges within the **‘innovation domain’**, specific to HIA as a tool and method, related to concerns about HIAs ‘evidence base’, as well as doubts about the value and use of systematic HIA processes and tools (Kraemer *et al*. 2014; Ramirez-Rubio *et al*. 2019; Thondoo *et al*. 2019, 2022; Kögel *et al*. 2020). The lack of perceived ‘relative advantage’ of using HIA over other IAs was also raised with some studies highlighting a perceived overlap with, and competing demands between, HIA and other IAs (Ison 2013; Mattig *et al*. 2017; Damari *et al*. 2018). A lack of appreciation of what HIA can offer in comparison to other IAs was also identified (Ison 2013; Quin *et al*. 2023). Perceived ‘complexity’ in implementing HIA in the ‘real world’ (Ison 2013; Kraemer and Gulis 2014; Busato and Grisotti 2022) or a particular HIA step such as the monitoring and evaluation (Green *et al*. 2020a) in addition to challenges concerning increased ‘costs’ and bureaucracy associated with its implementation were also commonplace (Mattig *et al*. 2017; Bever *et al*. 2021; Quin *et al*. 2023).

In the **‘individual domain’,** which captures the beliefs, roles and characteristics of those involved in implementing HIA, most of the potential barriers related to the ‘knowledge and beliefs’ construct. This included HIA being perceived as an administrative burden (O’Mullane 2014), differing perceptions of the purpose and objectives of HIA (Harris-Roxas *et al*. 2014; Liu *et al*. 2023), its recommendations being overly critical (Harris-Roxas *et al*. 2014) and low awareness, understanding and inconsistency within HIA practice (Ison 2013; Damari *et al*. 2018; Marincova *et al*. 2020; Liu *et al*. 2023). Feelings of superficial involvement amongst those involved in its implementation (Gamache *et al*. 2020), the belief that involvement of communities hinders progress in HIA (Fakhri *et al*. 2015) and perceptions of local knowledge being less valuable in comparison to expert knowledge (Negev *et al*. 2013) were also noted. Potential barriers related to ‘self-efficacy’ included feelings of inadequate training or experience (Ison 2013; Gamache *et al*. 2020; Jabot *et al*. 2020) in addition to lack of agency in implementing HIA, including its recommendations (Harris-Roxas *et al*. 2014; Kraemer *et al*. 2014; O’Mullane 2014).

In **the ‘process domain’**, concerning the strategies and activities used to implement HIA, challenges related to ‘engaging’ with different stakeholders were frequently reported. These ranged from obtaining government and institutional buy in (Bourcier *et al*. 2015; Fakhri *et al*. 2015; Damari *et al*. 2018) engaging with key stakeholders (Bourcier *et al*. 2015) such as decision makers and lack of involvement from those responsible for implementing HIA recommendations (Harris-Roxas *et al*. 2014). Similar engagement challenges were highlighted by Ramirez-Rubio *et al*. (2019) with regards to policy-makers accounting for time to engage in HIA. In relation to the engaging with communities specifically, a potential lack of trust in those implementing the HIA due to a perceived failure to disseminate past research to the community was also noted (Pursell and Kearns 2013). Likewise, lack of skills and confidence to fully engage with and interact with other actors involved in the HIA may also impede community engagement and involvement (Pursell and Kearns 2013). ‘Adaptation’ issues, in the context of over adaption of the HIA process, resulting in partial adherence to standard practice such as involving the public (Jabot *et al*. 2020) and the absence of a plan for implementing HIA recommendations (Morteruel *et al*. 2020) were also identified along with challenges related to ‘reflecting and evaluating’ the HIA after completion (Bourcier *et al*. 2015; Damari *et al*. 2018).

### Factors facilitating implementation: individual and process domain considerations

Those involved in implementing HIA, such as the individuals on the HIA steering or working group, are responsible for process related activities outlined in CFIR such as planning, doing, assessing context and needs, and engaging with key stakeholders. To create a practical route map for those involved in HIA, this section provides an overview of **‘process’** and **‘individual’** domain considerations identified within the literature explored that may facilitate improved HIA implementation when considered at the appropriate step within the HIA. Within the **‘individual domain’** and particularly relevant to the earlier steps of HIA, is ensuring diversity in the group carrying out the HIA in terms of skills, competencies, expertize, and local knowledge (Negev *et al*. 2013; Harris-Roxas *et al*. 2014; Bourcier *et al*. 2015; Haigh *et al*. 2015; Jabot *et al*. 2020; Kögel *et al*. 2020; Busato and Grisotti 2022). This includes ensuring key decision makers are included in the HIA (Harris-Roxas *et al*. 2014; Bourcier *et al*. 2015; Haigh *et al*. 2015). Some studies suggests that a close relationship with, and involvement of those with the capacity to act on the findings of the HIA, may increase the likelihood of the HIA recommendations being considered and adopted (Haigh *et al*. 2013; Morteruel *et al*. 2020). In the context of HIA ‘effectiveness’, which generally refers to the uptake of the recommendations produced from the HIA, the experience and capacity of the people involved matters (Haigh *et al*. 2015). Thus, directly involving people with agency to act on the HIAs findings is recommended (Harris-Roxas *et al*. 2014; Bourcier *et al*. 2015). Whilst Gamache *et al*. (2020) suggests that having an independent person without political constraints leading the HIA may encourage wider support and buy in from the onset.

**‘Process level’** facilitators related to ‘planning’ and the earlier stage of HIA includes engaging and building partnerships with stakeholders, establishing shared values, setting realistic expectations, explicit goals, and clearly defining roles and responsibilities within the HIA working/steering group (Haigh *et al*. 2015; Goff *et al*. 2016; Linzalone *et al*. 2017; Ramirez-Rubio *et al*. 2019; Gamache *et al*. 2020; Jabot *et al*. 2020). Several factors outside of the control of those involved in the HIA (i.e. the outer and inner setting) can influence the HIA process, and being aware of these influences is beneficial to those carrying out the HIA (Haigh *et al*. 2015). Thus, ‘assessing’ the context of the HIA, for example, the broader political context, recognizing potential policy windows and linking the HIA with actions and relevant policies outside of the HIA process (Kraemer *et al*. 2014; Fakhri *et al*. 2015; Damari *et al*. 2018; Gamache *et al*. 2020) may strengthen HIA implementation. For example, aligning the HIA focus with societal concerns is recommended to encourage social acceptance of the recommendations (Fakhri *et al*. 2015). Correspondingly, assessing which health related issues are gaining momentum can also encourage wider support and engagement with HIA (Bourcier *et al*. 2015).

‘Engaging’ and collaborating with key stakeholders is recommended throughout the lifecycle of the HIA (Bourcier *et al*. 2015; Buregeya *et al*. 2020; Thondoo *et al*. 2020a; Gamache *et al*. 2022). Multi- and inter-sectoral collaboration is an essential component of HIA implementation (Ison 2013; Kraemer *et al*. 2014; Haigh *et al*. 2015; Linzalone *et al*. 2018; Gamache *et al*. 2020; Jabot *et al*. 2020; Marincova *et al*. 2020; Jabot and Rivadeneyra-Sicilia 2022; Walpita and Green 2022; Liu *et al*. 2023). One strategy to facilitate key stakeholder engagement and collaboration in HIA is to engage as early as possible (Haigh *et al*. 2015) and encourage understanding and recognition of common goals and interests amongst key stakeholders (Gamache *et al*. 2020; Kögel *et al*. 2020; Morteruel *et al*. 2020). Research from New Zealand and Australia highlights how inter-sectoral involvement improved the quality of HIAs (Haigh *et al*. 2015) whilst research from Quebec, Canada identifies that such collaboration facilitated a shared language between institutions which can support future HIA collaborations (Gamache *et al*. 2020).

Although stakeholders vary by context, public engagement is fundamental to HIA in order to understand the needs and realities of the populations likely to be impacted by the project, plan or policy under appraisal (Roué-Le Gall and Jabot 2017; Fischer *et al*. 2024). Thus, ‘assessing the needs’ of the public and planning for their engagement is essential (Negev *et al*. 2013; Roué-Le Gall and Jabot 2017; Linzalone *et al*. 2018). This requires a more in-depth consideration and inclusion of the views and experiences of vulnerable groups (Negev *et al*. 2013; Busato and Grisotti 2022), building community capacity to engage (Pursell and Kearns 2013; Haigh *et al*. 2015), providing space for participation, and building partnerships between professional and community stakeholders (Negev *et al*. 2013; Morteruel *et al*. 2020; Thondoo *et al*. 2020a; Fischer *et al*. 2024). This also necessitates recognizing and addressing power discrepancies and cultural and language barriers between these communities and decisions makers (Negev *et al*. 2013; Pursell and Kearns 2013). One Group 2 study, promotes a ‘co-construction’ approach to citizen engagement requiring a willingness to move beyond the ‘consultation’ approach (Roué-Le Gall and Jabot 2017). Whilst a Group 3 study, specific to carrying out an HIA using a multi-cultural participatory model, suggests mapping stakeholders before the HIA commences in order to facilitate diverse representation on the committee (Negev *et al*. 2013).

Within the ‘doing’ construct, focussed on the optimizing the delivery of the HIA, several actions aimed at strengthening HIA implementation are identified. These include highlighting both direct health impacts and indirect health impacts (Fakhri *et al*. 2015; Goff *et al*. 2016), focussing on health determinants and health equities as outcomes (Kraemer *et al*. 2014; O’Mullane 2014; Fakhri and Harris 2021; Westenhöfer *et al*. 2023), acceptance of lay knowledge along with expert opinion (Thondoo *et al*. 2020a) and using and contextualizing local data with the scientific literature (Bourcier *et al*. 2015; Morteruel *et al*. 2020; Busato and Grisotti 2022). Producing clear and actionable recommendations with assigned responsibility (Bourcier *et al*. 2015) that reflect what the community want (Fischer *et al*. 2024) along with concise and tailored audience reports (i.e. decision makers and the public) may also improve HIA implementation (Gamache *et al*. 2020).

Ensuring transparency in the HIA process is important for building trust in HIA (Haigh *et al*. 2015; Berensson and Tillgren 2017; Linzalone *et al*. 2018; Ramirez-Rubio *et al*. 2019; Morteruel *et al*. 2020; Busato and Grisotti 2022; Gamache *et al*. 2022; Liu *et al*. 2023). This can be facilitated by providing profiles (expertize and role in the HIA) of those directly involved in the HIA, such as the HIA steering/advisory committee, clearly documenting the decision-making process throughout each stage of the HIA (ibid), increased scrutiny of the monitoring stages, and bridging the evidence gap through HIA case studies illustrating health outcomes (Fischer *et al*. 2024). This may also be supported through follow up processes that identify which recommendations were accepted or otherwise and explaining why this was the case (Gamache *et al*. 2022; Fischer *et al*. 2024). This ties in with ‘reflecting and evaluating’ the HIA process via periodic assessment of stakeholders’ interests and continued information sharing between those involved in implementing HIA and the public (Bever *et al*. 2021; Liu *et al*. 2023). Table 1 presents a series of potential measures, derived from individual and process domain influences, and aligned with the relevant HIA steps where they ought to be considered.

### Potential strategies to build HIA support, capacity, and awareness: innovation, inner, and outer domains

Building HIA support, capacity, and awareness is essential to progressing HIA (Goff *et al*. 2016; Roué-Le Gall and Jabot 2017; Kögel *et al*. 2020; Jabot and Rivadeneyra-Sicilia 2022; Liu *et al*. 2023). Building a case for HIA by highlighting its ‘relative advantage’ may be achieved by identifying how HIA can improve rather than hinder development (Damari *et al*. 2018), addressing confusion between HIA and other types of IAs (Jabot and Rivadeneyra-Sicilia 2022), building awareness of how HIA can contribute to various stakeholders agendas (O’Mullane 2014; Jabot *et al*. 2020) and being clear on what HIA can and cannot achieve (Mattig *et al*. 2017). Collaborating with media may also facilitate broader awareness and increase stakeholder familiarity with HIA and its value (Marincova *et al*. 2020). For example, a case study of a participatory HIA (Linzalone *et al*. 2017) in Italy held a forum targeting local media agencies and the public at the beginning of the HIA to build trust and create awareness of HIA. Likewise, creating more favourable ‘local conditions’ supportive of HIA may assist in building HIA capacity and sustaining HIA into the future. For example, several studies suggest introducing HIA within educational settings (Damari *et al*. 2018; Marincova *et al*. 2020; Gamache *et al*. 2020; Liu *et al*. 2023).

Several studies also point to the benefits of building ‘partnerships and connections’ in terms of access to resources and support that can be derived from external HIA Networks, including the WHO Healthy Cities Network (Ison 2013; Kraemer *et al*. 2014; O’Mullane 2014; Mattig *et al*. 2017; Roué-Le Gall and Jabot 2017). Building partnerships between community-based organizations and health practitioners, local and regional authorities focussed on social and health related matters, and universities would also support improved access to resources required for implementing HIA (Goff *et al*. 2016; Bever *et al*. 2021; Jabot and Rivadeneyra-Sicilia 2022). Providing access to HIA training (Ison 2013; O’Mullane 2014; Kraemer *et al*. 2014; Goff *et al*. 2016; Linzalone *et al*. 2018; Jabot *et al*. 2020; Walpita and Green 2022; Jabot and Rivadeneyra-Sicilia 2022; Quin *et al*. 2023; Liu *et al*. 2023) developing accessible, easy to use and adaptable guidelines and tools (Linzalone *et al*. 2018; Jabot *et al*. 2020; Thondoo *et al*. 2020a; Quin *et al*. 2023) and allocating funding for HIA (Fakhri *et al*. 2015; Linzalone *et al*. 2018; Gamache *et al*. 2020; Morteruel *et al*. 2020; Thondoo *et al*. 2020b) would also assist in supporting and sustaining HIA. However, research from Spain identifies how more resources were not associated with greater effectiveness in the context of low political support (Morteruel *et al*. 2020) whilst evidence from Sweden suggests that even when a political decision has been made to utilize and implement HIA, realizing this intention and sustaining HIA can be difficult (Berensson and Tillgren 2017). Nonetheless, several studies highlight the importance of building supportive political and legal context for progressing HIA (Roué-Le Gall and Jabot 2017; Ramirez-Rubio *et al*. 2019; Jabot and Rivadeneyra-Sicilia 2022). Table 2 presents potential strategies identified within the literature, that may begin to address the primary barriers located within the **inner**, **outer,** and **innovation** domains and specifically in relation to the challenges of ‘access to resources, building partnerships, and connections’, creating supportive ‘local conditions’ and creating awareness of *‘*the relative advantage of HIA’.

## DISCUSSION

This scoping review provided an overview of a range of factors influencing HIA implementation from a diverse literature base. Consistent with existing literature, barriers affecting HIA implementation include the lack of HIA knowledge, resources, awareness, and legislative support (Winkler *et al*. 2020; Green *et al*. 2022; WHO 2023a, 2023b). This study adds to the existing HIA literature base by illustrating the level in which these challenges exist, allowing for the identification of potential measures that may strengthen HIA practice (Table 1) and build broader support, capacity, and awareness of HIA (Table 2).

Importantly, the use of CFIR in the HIA context draws particular attention to the earlier steps of HIA. HIA literature and some guidance generally refer to five steps, with the appraisal step being ‘the core’ and the most labour intensive (Pyper *et al*. 2022). Based on the potential barriers and facilitators synthesized here, the earlier steps of HIA appear to be especially important in assisting the practical application and implementation of HIA, including HIAs overarching core values and in particular equity and participation (Table 1). For example, McDermott *et al*. (2024) criteria for assessing equity and participation within existing HIA frameworks includes the participation of groups currently facing inequities in the screening and scoping steps of HIA. This recommendation is also reflected in the literature explored here (Roué-Le Gall and Jabot 2017; Gamache *et al*. 2022). Based on albeit a limited number of case studies presented here (Group 3 studies) this does not appear to be standard practice currently. Considering that equity and participation are core values within both HIA and health promotion practise more broadly, clearer reporting of who was involved, the rationale for their involvement, what level of power and control they had in the process, and the HIA step/s that they contributed to, may be beneficial for future practice and in facilitating greater transparency in HIA reporting and building trust in HIA.

Moreover, the earlier HIA steps determine whether an HIA is needed, the scope of the HIA, the prioritizing of potential impacts, the research questions, and the methods that will be used to gather and appraise the evidence and thus form a crucial component of HIA that lays the foundation for the proceeding steps. The inclusion of potentially affected populations in these decisions requires some knowledge of potential impacts and populations likely to be impacted by the policy, plan, and programme at the screening stage of the HIA. Stakeholder mapping before a HIA begins (Negev *et al*. 2013) along with insight from the literature regarding potential health impacts (Sheffield *et al*. 2014) at the screening rather than the scoping step may be helpful in addition to planning for adequate time and resource allocation to involve impacted communities.

CFIR also draws further attention the importance of additional process related activities, such as assessing needs and context at various stages throughout the HIA and critically, before the process begins. This included assessing the needs of individuals involved in the HIA (Haigh *et al*. 2015; Linzalone *et al*. 2018; Busato and Grisotti 2022), the local context (inner setting) and wider contextual factors (outer domains) in order to take advantage of opportunities to link the HIA with wider societal and policy concerns (Kraemer *et al*. 2014; Haigh *et al*. 2015; Damari *et al*. 2018; Gamache *et al*. 2020; Fakhri and Harris 2021). Further attention to and reporting of these process related activities may improve HIA practice and ultimately wider support for HIA.

Building HIA support, capacity and awareness is essential to progressing HIA (Goff *et al*. 2016; Roué-Le Gall and Jabot 2017; Kögel *et al*. 2020; Jabot and Rivadeneyra-Sicilia 2022) and based on the literature explored here, this is reliant on supportive inner and outer settings and addressing challenges specific to HIA as a method and tool. Notwithstanding the significant legal and policy gaps which vary by country, building support for HIA in the inner and outer settings may be facilitated by illustrating the relative advantage of HIA over other IA approaches, promoting greater awareness of benefits of HIA to decision and policy makers and members of the public using evidence from case studies, and strengthening HIA practice. This would support addressing multiple challenges identified across domains ranging from scepticism of HIA (individual domain) to difficulties in obtaining buy in and resources from decisions makers within organizations (inner domain) and broader policy circles (outer domain).

In terms of implementing HIA and focussing on what HIA practitioners have control over/degree of agency in, such as creating the HIA implementation team, aligning HIA practice with the guiding principles for HIA and facilitating essential process related activities, CFIR may be a particularly useful tool for planning an HIA. It offers a concise framework that could be used to assist in clearly identifying specific roles and skills required within the HIA team and the specific process related activities that may strengthen HIA practice.

## LIMITATIONS

This study has several limitations including potential publication bias and an over representation of studies from high-income countries. This scoping review relied on peer-reviewed studies available in English, potentially overlooking valuable insights from grey literature and non-English studies including consultant led HIAs. This is important considering that many HIA reports are not published in peer-reviewed literature (Kim *et al*. 2024). For example, there is a significant body of HIA in literature in Francophile regions. Whilst Group 2 studies did encompass 103 HIA reports, these were mostly focussed on the urban environment in high income countries. These limitations reduce the generalizability of findings to lower and middle-income contexts, as well as peri-urban and rural settings. Moreover, whilst there is some consistency in the barriers, facilitators and opportunities identified, the small number of studies included should also be taken into account when interpreting the results.

Additional methodological limitations are also noteworthy. This scoping review aimed to synthesize potential barriers and enablers of HIA implementation using CFIR rather than to provide a detailed account of the context of implementation which likely varies significantly across countries. This led to difficulties in categorizing some barriers and facilitators related to inner and outer setting domains and in some instances; these influences were coded to both domains. For example, access to resources such as funding, guidance and tools to implement HIA may be dependent on both the inner setting and the outer setting domains depending on the country and organizational context. However, this detail does not change the finding that access to these resources for building capacity are essential to progress and sustaining HIA. Moreover, whilst not all CFIR constructs and sub-constructs were utilized in classifying the factors influencing HIA implementation, this is likely due to the nature of a scoping review synthesizing existing literature and various types of studies rather than the constructs themselves being irrelevant to HIA implementation. CFIR may be more useful for future prospective HIA research when applied at the study design phase, as is the intended purpose of CFIR 2.0 (Damschroder *et al*. 2022). This research relied on the literature base included in the scoping review to identify potential HIA implementation barriers and facilitators. Future research could consider using the Expert Recommendations for Implementing Change tool (Powell *et al*. 2015) to match the identified barriers with potential solutions.

Despite these limitations, and some initial challenges applying CFIR retrospectively, CFIR provides a useful and adaptable framework for exploring HIA implementation. CFIRs application to HIA implementation identifies operational considerations for HIA practitioners and draws attention to process and individual influences that can progress HIA in becoming a more practical and transparent operational tool. Future HIA practitioners may therefore wish to consider CFIR as a guiding framework when planning, designing and implementing HIA.

## CONCLUSION

This scoping review presents a novel exploration of HIA from an IS perspective. Utilizing CFIR as a framework to explore potential factors influencing HIA implementation enabled the distinction of determinants operating across CFIR domains. This allowed for the identification of a range of practical considerations for those involved in HIA and at specific steps in the HIA process, along with the identification of potential strategies aimed at building broader HIA awareness, capacity and support necessary to sustain HIA into the future. HIA is a core component of health promotion practice that can facilitate a HiAP approach. CFIR provides a useful guiding framework to support HIA planning, practice, and implementation and in particular draws attention to the need for adequate time and attention to be given to the earlier steps of HIA and process related activities.

## Supplementary Material

daaf080_Supplementary_Data

## Data Availability

The data underlining this article are available in the article and in its online supplementary materials.
