# Supplementary material for: Routemap for health impact assessment implementation: scoping review using the consolidated framework for implementation research
Source: Health Promot Int. 2025 Jun 30;40(3):daaf080. doi: 10.1093/heapro/daaf080 (PMC12208066; doi:10.1093/heapro/daaf080)
Supplement: daaf080_Supplementary_Data [file daaf080_supplementary_data.zip › SM File 1. PCC guided inclusion criteria.docx]

Table 1 : PCC Pneumonic guided inclusion criteria

|  | **Inclusion criteria** | **Exclusion criteria** |
| --- | --- | --- |
| **Population/participants:** | Studies that report on HIA implementation based on the experience of those who were/are involved in the HIA process, or the authors of the paper were part of the HIA team and are reporting on its implementation | Studies not focussed on the experiences of (i) those involved in HIA or (ii) those likely to be involved in future HIA implementation or (iii) by examining individual HIA reports, or case studies |
| **Concept:**  **HIA and Implementation** | Peer reviewed journal articles focussed on HIA and its implementation and provides information relating to the process of implementation and/or factors influencing HIA implementation process | Peer reviewed journal articles not focussed on HIA implementation and the factors influencing implementation (i.e. guidelines for HIA development, modelling studies, papers focussed on methods of assessment, or specific health outcomes) |
| **Context:** | Peer reviewed journal articles primarily focussed on HIA implementation | Peer reviewed journal articles where HIA is part of another IA (i.e. integrating HIA within EIA) |
| **Types of studies** | Peer reviewed research not limited by research design.  Studies published in English | Commentary, conceptual, opinion articles, grey literature, and articles not available in English |
