# Supplementary material for: Routemap for health impact assessment implementation: scoping review using the consolidated framework for implementation research
Source: Health Promot Int. 2025 Jun 30;40(3):daaf080. doi: 10.1093/heapro/daaf080 (PMC12208066; doi:10.1093/heapro/daaf080)
Supplement: daaf080_Supplementary_Data [file daaf080_supplementary_data.zip › SM File 2. Included literature.docx]

Table 1: Metadata from included studies

| **Title** | **Authors, year** | **Research questions/objectives** | **Country/region** | **Method** | **Study Participants where applicable** | **Classification for scoping review** | **No. of HIA case studies, reports included if applicable** |
| --- | --- | --- | --- | --- | --- | --- | --- |
| Health impact assessments in Spain: Have they been effective? | (Morteruel et al., 2020) | To assess the effectiveness of five HIAs carried out in Spain at the local level, and the role played by context and process factors on these impacts | Spain | Qualitative interviews (n=14) | HIA participants from various sectors | Group 1 | NA |
| Implementation of health impact assessment in Danish municipal context | (Kræmer et al., 2014) | Knowledge and attitudes towards HIA, how far the Municipalities are in Implementing HIA and what are the barriers and facilitators. | Denmark | Qualitative interviews (n=12) | 12 interviews with one politician and one administrator from 6 municipalities. | Group 1 | NA |
| Framework for participatory quantitative health impact assessment in low-and middle-income countries | (Thondoo, et al., 2020) | What are the different components of a participatory quantitative model that can influence HIA implementation in LMICs? | Mauritius | Qualitative, field notes, focus groups, interviews (n=14) | Focus group discussions and feedback exercises with 14 stakeholders who participated in HIA “laypersons, health practitioners and policymakers…” | Group 1 | NA |
| Health impact assessment in the process of implementation of hydroelectric plants: methodological contributions | (Busato & Grisotti, 2022) | Analyses the contributions of specialists on the stages of assessment of health impacts in Brazil, described in the documents of the Ministry of Health, and aims to contribute to the improvement of HIA methods, especially for the areas of implementation of hydroelectric plants | Brazil | Qualitative interviews (n=18) | Specialist from 8 Brazilian higher education institutes | Group 1 | NA |
| A comparative analysis of health impact assessment implementation models in the regions of monteregie (Quebec, canada) and nouvelle-aquitaine (france) | (Jabot et al., 2020) | To compare HIA implementation models in two regions: Nouvelle-Aquitaine (France) and Montérégie (Québec, Canada) | France & Canada | Comparative case study. Field data and reflective analysis. analysis of documents  and reports describing the two implementation schemes, informal interviews with key stakeholders and direct observations during seminars, workshops, and other HIA-related meetings held in the two regions. | key stakeholders (regional and municipal policy-makers, departmental oﬃcials and technical staﬀ, public health oﬃcials, HIA consultants, and practitioners), plus five authors (evaluators, practitioners, and assessors) in the HIA schemes under study | Group 1 | NA |
| What makes health impact assessments successful? Factors contributing to effectiveness in Australia and New Zealand Health policies, systems and management in high-income countries | (Haigh et al., 2015) | To ascertain which factors are associated with increased or reduced effectiveness of HIAs in changing decisions and in the implementation of policies, programs or projects | Australia & New Zealand | Mixed: survey and structured interviews (n=34) and retrospective multiple case studies using qualitative methods | i) key decision-makers who were responsible for taking the recommendations forward  (ii)practitioners who conducted HIAs and (iii)other stakeholders (including community stakeholders) involved in the process. | Group 1 | NA |
| Experiences and needs of licensed health risk assessors conducting health impact assessment in the Czech Republic | (Marincova et al., 2020) | Explores the utilization of health impact assessment (HIA) among HIA certificate holders, and to ascertain their opinions on the current situation in the Czech Republic and on the possible future nationwide implementation of health impact assessment | Czech Republic | Questionnaire- open and closed (n=12) | Holders of professional competence certificates in the field of health impact assessment. | Group 1 | NA |
| Walkability and urban built environment: a systematic review of health impact assessments (HIA) | (Westenhöfer et al., 2023) | How and by whom are HIAs of walkability implemented in practice | Multi | SR – partial relevance | NA | Group 2 | 40 HIAs |
| Use of health impact assessments in the housing sector to promote health in the United States, 2002-2016 | (Bever et al., 2021) | Describes key characteristics of housing HIAs (n=54) conducted in the U.S., including the types of decisions examined, types of organizations leading the assessments, and location; (2) examined common methods and approaches used in housing HIAs; and (3) documents the effects of housing HIAs on policymaking, where data are available, and provide three illustrative examples of how HIAs can influence housing decisions | US | Systematically reviewed 54 HIAs using various forms of data beyond HIA reports | NA | Group 2 | 54 HIAs |
| Using health impact assessments to implement the sustainable development goals in practice: a case study in Wales | (Green, Gray, et al., 2020) | Reﬂects on the process of carrying out the HIA and the methods used. - Describes HIA undertaken on a proposed major electricity cable connection development in Wales. Discusses how this HIA was the catalyst for considered thinking about the HIA process and how it can be used to consider and implement the Well-being Goals in practice, and initiates further thinking about how this can be extended to implement and address the SDGs at a local, regional and national level | Wales | Case study/reflection on HIA process. Mixed methods used in HIA | NA | Group 3 | NA |
| The Built Environment and Health in Low- and Middle-Income Countries: a Review on Quantitative Health Impact Assessments. | (Thondoo et al., 2022) | Reviews selected HIA case studies from LMICs and reports the methods and tools used to support further implementation of quantitative HIAs in cities of LMICs | Algeria, Brazil, China, India, Iran, Kenya, Thailand, Turkey, and Mauritius | analysed a subset of case studies selected from a systematic review on HIAs conducted in LMICs published in 2019 | NA | Group 2 | 24 HIAs |
| The Elaboration of an Intersectoral Partnership to Perform Health Impact Assessment in Urban Planning: The Experience of Quebec City (Canada). | (Gamache et al., 2020) | Presents the approach developed with partners, the process, and the outcomes of HIA implementation after seven years of interinstitutional collaborations in Quebec City. | Canada | Direct observation/documentary/minutes of meeting | Consultants from the municipality of Quebec (QC)- the Regional Public Health Authority (RPHA), the non-governmental organism (NGO) Vivre en Ville (VEV), and the National Collaborating Centre for Healthy Public Policy (NCCHPP) and subcommittees | Group 1 | NA |
| Process, Practice and Progress: A Case Study of the Health Impact Assessment (HIA) of Brexit in Wales | (Green, Ashton, et al., 2020) | reflects on the process of carrying out the HIA and the methods used. It discusses the stages of the HIA, and shares the findings and reflections of implementation which will be beneficial to other HIA practitioners and policy makers. | Wales | Case study, HIA carried out in 6 months. Mixed methods | See SM File 3 | Group 3 | NA |
| Participatory quantitative Health Impact Assessment of urban Transport Planning: A case study from Eastern Africa. | (Thondoo, et al., 2020a) | What are the major risk exposures and health impacts derived from urban transport planning policies in an African city? | Mauritius | Mixed methods:  estimated health and economic impacts associated to transport scenarios with Qualitative itative data and quantitative comparative risk assessment methods. No timeline provided. | See SM File 3 | Group 3 | NA |
| Health impact assessment of a watershed development project in southern India: a case study | (Pradyumna et al., 2021) | presents the main HIA implementation issues encountered in the last two years and the solutions applied or envisaged | Southern India | Standalone case study of an HIA of a proposed project in the semi-arid Kolar district in the southern part of India. | See SM File 3 | Group 3 | NA |
| Transportation Matters: A Health Impact Assessment in Rural New Mexico | (Del Rio et al., 2017) | case study of the impacts of public transportation in rural areas in the U.S./Mexico border region. | New Mexico | North American HIA Practice Standards Working Group 6 step approach  Mixed methods: surveys of community members, key informant interviews, a focus group with community health workers, and passenger surveys | See SM File 3 | Group 3 | NA |
| How do experts define relevance criteria when initiating Health Impact Assessments of national policies? | (Kraemer & Gulis, 2014) | to present how the Danish Disease Prevention Committee (DDPC) members and HIA experts understand and perceive when HIA is “relevant”, i.e. “presumed to have a direct and documented effect on the health and morbidity of the citizens”. | Denmark | Mixed methods: interviews with DDPC and survey of HIA experts | Semi-structured interviews (n=10) with DDPC members (areas of expertise included; medical, environmental, social, economic and technical sciences).  Survey (n=100) of HIA experts defined as HIA practitioners and HIA academics | Group 1 | NA |
| Implementing Health Impact Assessment Programs in State Health Agencies: Lessons Learned from Pilot Programs, 2009-2011 | (Goff et al., 2016) | to identify the role of state health agencies (SHAs) in building capacity for conducting HIAs and the key components of initiatives that produced effective HIAs and HIA programs | United States (California, Minnesota, Oregon, and Wisconsin) | Reviewed program reports from the pilot state health agencies that between 2009 and 2011, created HIA programs to provide HIA training, conduct HIAs, and build practitioner networks. | Information from 3 primary sources: (1) the pilot states’ end-of-year grant reports; (2) the pilot states’ presentations of their HIA capacity building programs at national conferences and meetings; and (3) individual HIA reports. | Group 2 | 9 program reports |
| Implementing **Health Impact Assessment policy** on infrastructure development in the London Borough of Tower Hamlets | (Quin et al., 2023) | explores key challenges to the development and adoption of a Health Impact Assessment (HIA) Policy within the infrastructure development setting in the London Borough of Tower Hamlets. | UK | Case study: mixed methods. Structured lit review, policy analysis, knowledge, attitudes and Practise (KAP) survey and stakeholder interviews | Survey - exact number not provided.  Survey (n 20-25) of civil servants in the London Borough of Tower Hamlets  Interviews (n=5) two from Development Management, one from Planning Policy, one from Public Health and one from Infrastructure Planning. | Group 1 | NA |
| Implementing health impact assessment at national level: An experience in Iran | (Damari et al., 2018) | Proposes a model for implementing HIA in Iran | Iran | Case study, Qualitative itative research. Interviews and focus groups. | Interviews (n=15) with ‘informed people’:  members of permanent commission of the SCHFS (representatives of members of different ministries and organizations )  Focus group with ‘experts’(n=6) | Group 1 | NA |
| Health Impact Assessment (HIA) of a fluvial environment recovery project in a medium-sized Spanish town. | (Kögel et al., 2020) | presents the results of the implementation of the HIA on the urban redesign of the Llobregat fluvial area in Sant Andreu, Spain. | Spain | Standalone case study; A prospective nonquantitative HIA. Mixed methods | See SM File 3 | Group 3 | NA |
| Urban health: an example of a "health in all policies" approach in the context of SDGs implementation. | (Ramirez-Rubio et al., 2019) | To understand the links between social determinants of health, environmental exposures, behaviour, health outcomes and urban policies within the SDGs, following a HiAP rationale; to review and analyze the key elements of a HiAP approach as an accelerator of the SDGs in the context of urban and transport planning; and to describe lessons learnt from practical implementation of HIAs in cities across Europe, Africa and Latin-America. | Multiple | Review paper.  Review of HIAs in a number of cities worldwide based on the experiences of the co-authors of this paper involved in HIA | NA | Group 2 | 17 HIAs |
| Systematic Literature Review of Health Impact Assessments in Low and Middle-Income Countries. | (Thondoo et al., 2019) | systematically reviews, geographically maps, and characterises HIA activity in LMIC and applies a process evaluation method to identify factors which are important to improve HIA implementation in LMICs | LMICs | systematic review focussing on process evaluation | NA | Group 2 | 57 HIAs |
| Institutionalizing Health Impact Assessment: A consultation with experts on the barriers and facilitators to implementing HIA in Italy. | (Linzalone et al., 2018) | To map the factors that inﬂuence HIA introduction and use, and to identify speciﬁc local barriers and opportunities for the Italian context. (Recommendations are provided for a generalized implementation plan which may contribute to a greater institutionalization of HIA) | Italy | Mixed methods. A comparative review of the international literature was carried out to identify the general mechanisms that enhance the implementation of HIA. Subsequently, various private and public technical experts were consulted to facilitate the introduction of HIA into the mandatory assessment procedures of EIA and SEA in Italy. | Consultation workshop with 11 participants currently involved in or potentially will be involved in HIA. Participants selected based on their particular interest in the issue, business sector, and expertise in EIA and SEA processes | Group 1 | NA |
| Health impact assessment on urban development projects in France: finding pathways to fit practice to context. | (Roué-Le Gall & Jabot, 2017) | To analyse the practice of HIA implemented in urban settings in France in order to find ways of adapting them to the specific context of urban project development | France | Case study of four HIAs (at various stages of progress) applied to urban development project | NA | Group 2 | 4 HIAs |
| HIA in Switzerland: strategies for achieving Health in All Policies. | (Mattig et al., 2017) | Review the status of Health Impact Assessment (HIA) in Switzerland and assess whether HIA can be used to implement Health in All Policies by exploring local (bottom- up) and federal (top-down) processes of HIA institutionalization in Switzerland. | Switzerland | The methods include expert opinion and an extensive literature review, as well as targeted interviews with key informers in the regions of Geneva, Jura and Ticino | No details provided on who the interviews were with other than geographical location | Group 1 | NA |
| Participatory health impact assessment used to support decision-making in waste management planning: A replicable experience from Italy | (Linzalone et al., 2017) | Describes the participatory HIA process used in deciding on the possible doubling of the municipal solid waste incinerating plant located near the city of Arezzo, Italy. | Italy | Standalone case study of the application of a Participatory HIA. Mixed method HIA | See SM File 3 | Group 3 | NA |
| Proposing a framework for Health Impact Assessment in Iran. | (Fakhri et al., 2015) | To Qualitative itatively identify the various factors required to progress HIA in Iran and to develop a conceptual framework for progressing HIA in Iran including all factors influencing HIA planning and practice | Iran | Qualitative interviews | Key informant interviews with those experienced in HIA and related areas (n = 14) | Group 1 | NA |
| Implementing the legal provisions for HIA in Slovakia: an exploration of practitioner perspectives. | O Mullane  (O’Mullane, 2014) | Explores the perceptions of key informants and practitioners of the upcoming enforcement of the HIA part of legislation | Slovakia | Qualitative Open ended questionnaire | Participants (n=14): leading public health practitioners in the Slovak state public health system, and follow up semi -structured interviews (n=3) | Group 1 | NA |
| The effectiveness of health impact assessment in influencing decision-making in Australia and New Zealand 2005-2009 | (Haigh et al., 2013) | Identify if and how HIAs changed decision-making and implementation and impacts that participants report following involvement in HIAs | New Zealand & Australia | Mixed methods: questionnaires with follow-up interview, semi-structured interviews with (i) key decision-makers who were responsible for taking the recommendations forward and those who could influence them, (ii) HIA assessors and (iii) other stakeholders involved in the process along with document collation | Questionnaire (n=48) and follow up interviews(n=34): HIA practitioners  who had designed and implemented the HIAs. A further 33 semi-structured interviews with various stakeholders (decision making organisations, those involved in the HIA and other stakeholders such as members of the steering groups) involved in the 11 HIAs chosen for in-depth-case study analysis. | Group 1 | NA |
| Health impact assessment in a network of European cities. | (Ison, 2013) | Explores the implementation of HIA in cities across Europe with differing economies and sociopolitical contexts | Multiple European cities | Two electronic surveys – Qualitative (n=58 cities) | Members of the healthy cities network (58 cities) involved in HIA. No further detail provided in relation to responders | Group 1 | NA |
| Contribution analysis to analyze the effects of the health impact assessment at the local level: A case of urban revitalization | (Buregeya et al., 2020) | To analyse HIA impacts on the revitalization of road infrastructure, parks and green spaces, and residential housing by examining the HIA process and its influence at the municipal level, assessing its impacts and exploring its impacts on health. | Canada | Mixed methods: in-depth interviews with stakeholder (n=19), documentary analysis, scoping review, and contribution analysis to analyse the effects of HIA | People involved in the HIA process (n=5) and organisations with detailed knowledge of issues in the city center relative to the determinants of health (n=9) | Group 1 | NA |
| Health impact assessment (HIA) of political proposals at the local level: successful introduction, but what has happened 15 years later? | (Berensson & Tillgren, 2017) | To describe and analyse the implementation of HIA in municipalities and regions, at two time points ( 3 years and 15 years after HIA was launched at the local and regional levels) in Sweden. | Sweden | Two cross-sectional questionnaires sent by to municipalities and regions in Sweden in 2001 and 2013. | 2001: municipalities (n= 289) and health care directors in regions (n = 21)  2013: strategic sample of municipalities that were members of four different national networks with a focus on applied public health work and the UN Convention on the Rights of the Child (n= 36), and to public health managers in regions (n = 21). | Group 1 | NA |
| Internationally validating a conceptual framework for health impact assessment | (Fakhri & Harris, 2021) | Exploring factors that influence HIA to develop a conceptual framework for the establishment and practise of HIA | Multi-country | Quantitative survey | International academics and practitioners (n = 38) inclusion criteria: Publishing peer review papers in the HIA field or conducting HIA projects | Group 1 | NA |
| The use of health impact assessments performed in Quebec City (Canada)-2013-2019: Stakeholders and participants' appreciation | (Gamache et al., 2022) | To 1) analyse the implementation of the HIA procedure in the urban planning process and to identify levers, constraints and potential for improvement; 2) analyse the deployment of the HIA approach, including the stakeholders' involvement, the ability of HIA to support decision making and to make changes in the stakeholders' practice; and 3) identify success factors and challenges for the integration of HIA into the urban planning process. | Canada | Mixed methods: 1) elaborating a conceptual  framework based on a literature review as well as a review of existing conceptual frameworks; 2) conducting a survey to obtain HIAs' participants (stakeholders) opinion regarding their experience and the HIA process; and 3) the analysis of the gathered data | Stakeholders (HIA participants )survey (n=25): included SC-HIA members (n = 5), project coordinators (n=9) | Group 1 | NA |
| Health Impact Assessment (HIA): A Comparative Case Study of Sri Lanka and Wales: What Can a Developing Country Learn From the Welsh HIA System? | (Walpita & Green, 2022) | What are the barriers and opportunities for implementing HIA in Sri Lanka | Sri Lanka/Wales | Comparative case study: A mixed methodology of concurrent triangulation, combining document analysis, interviews, and observations | Interviews with members of the Welsh Health Impact Assessment Support Unit (WHIASU)  Observations by taking part in monthly team meetings and workshops of WHIASU for 7 months.  Interviews (n=8) participants from Sri Lanka, including ofﬁcials of the Ministry of Health, Central Environmental Authority, and WHO Collaborating Centre for Occupational Health and Safety; public health consultants; and members of academia | Group 1 | NA |
| Stakeholder participation in health impact assessment: A multicultural approach | (Negev et al., 2013) | Presents and analyses a case study of participatory HIA focused on land use and involving diverse stakeholders. | Israel | Qualitative multi-method Case study, interviews, focus groups, observation | See SM File 3 | Group 3 | NA |
| Health impact assessments for environmental restoration: the case of Caño Martín Peña | (Sheffield et al., 2014) | This HIA examined the potential health impacts of the dredging and various infrastructure interventions of the proposed environmental and development plan in this environmental justice community | Puerto Rico | HIA Case study based on a six-part HIA methodology over 15 months | See SM File 3 | Group 3 | NA |
| An evaluation of health impact assessments in the United States | (Bourcier et al., 2015) | to assess results of health impact assessments (HIAs) in the United States and to identify elements critical for their success. | US | mixed-methods comparative case study (literature review; site visits; interviews with investigators, stakeholders, and decision makers for 23 HIAs in 16 states that were completed from 2005 through 2013; and a Web-based survey HIA practitioners). | Interviews (n=166 – HIA teams, 1-2 decision makers and 1-2 community stakeholders) with investigators, stakeholders, and decision makers for 23 HIAs  Survey of HIA practitioners (n=144) | Group 1 | NA |
| Impacts of an HIA on inter-agency and inter-sectoral partnerships and community participation: lessons from a local level HIA in the Republic of Ireland | (Pursell & Kearns, 2013) | Evaluates the impacts of a health  impact assessment (HIA) on community participation, inter-sectoral and inter-agency partnership in local decision and policy-making processes. | Ireland | Qualitative research design to gather accounts of the HIA’s impacts from the core stakeholders. | Interviews (n=21) 11 from the initial 20 member HIA SG and 10 from the community and other organizations and agencies involved in the HIA | Group 1 | NA |
| Evaluating the impact of equity focused health impact assessment on health service planning: three case studies | (Harris-Roxas et al., 2014) | To investigate whether EFHIA could improve the development and implementation of plans within the health sector; which changes occurred as a result of conducting and implanting the recommendations of EFHIAs; and whether EFHIAs are effective and under what circumstances | Australia | Qualitative retrospective case study of three completed EFHIAs: 14 semi-structured interviews | Interviews (n=14) with those involved in EFHIA and responsible for developing health sector plan and/or acting on the EFHIA recommendations | Group 1 | 3 |
| Health impact assessment institutionalisation in France: state of the art, challenges and perspectives | (Jabot & Rivadeneyra-Sicilia, 2022) | How can HIA be institutionalised in France | France | Structured lit review and multi case study | NA | Group 2 | 41 HIAs |
| Health Impact Assessment (HIA) of a Daily Physical Activity Unit in Schools: Focus on Children and Adolescents in Austria Up to the 8th Grade | (Movia et al., 2022) | Health Impact Assessment (HIA) examined the potential impact of a daily physical activity unit in Austrian schools, with a focus on children and adolescents up to eighth grade | Austria | Standalone case study | Focus groups (n=4) parents, principals, school management, admin, physical activity experts.  Note: Child’s voice captured via parents. | Group 3 | 1 |
| Health impact assessment in two planning  projects in England: reflections on normative  effectiveness | (Fischer et al., 2024) | To examine normative effectiveness of HIA in English spatial planning on the basis of two case studies that had previously been identified as examples of good practice with regards to how they were conducted procedurally and the Qualitative ity of documentation | England | Qualitative retrospective study of two HIAs | Semi-structured interviews (n=7) with public health, planning and other actors originally involved in the HIAs were conducted in 2021 | Group 1 | 2 |
| Current status and influencing factors of policy identification in health impact assessment: a case study of Zhejiang Province | (Liu et al., 2023) | Three objectives: (i) to construct an initial conceptual model of the dimensions and influencing factors of policy identification status; (ii) analyse the current status of policy identification to understand the level of identification, concern, participation, attitudes and other aspects among relevant individuals towards the HIA system; and (iii) to refine and summarize the final set of factors influencing policy identification that are suitable for the HIA field | China | Mixed methods: interviews and surveys | Semi-structured interviews (n=30): 15 with members of the HIA system leadership group from pilot cities/counties and 15 experienced practitioners specializing in in HIA  Survey (n=665) of personnel responsible for HIA in Zhejiang Province | Group 1 | NA |

**References**

Berensson, K., & Tillgren, P. (2017). Health impact assessment (HIA) of political proposals at the local level: Successful introduction, but what has happened 15 years later? *Global Health Promotion*, *24*(2), 43–51. https://doi.org/10.1177/1757975916683386

Bever, E., Arnold, K. T., Lindberg, R., Dannenberg, A. L., Morley, R., Breysse, J., & Pollack Porter, K. M. (2021). Use of health impact assessments in the housing sector to promote health in the United States, 2002–2016. *Journal of Housing and the Built Environment*, *36*(3), 1277–1297. https://doi.org/10.1007/s10901-020-09795-9

Bourcier, E., Charbonneau, D., Cahill, C., & Dannenberg, A. L. (2015). An Evaluation of Health Impact Assessments in the United States, 2011–2014. *Preventing Chronic Disease*, *12*, 140376. https://doi.org/10.5888/pcd12.140376

Buregeya, J. M., Loignon, C., & Brousselle, A. (2020). Contribution analysis to analyze the effects of the health impact assessment at the local level: A case of urban revitalization. *Evaluation and Program Planning*, *79*, 101746–15. https://doi.org/10.1016/j.evalprogplan.2019.101746

Busato, M. A., & Grisotti, M. (2022). Health impact assessment in the process of implementation of hydroelectric plants: Methodological contributions. *Ambiente & Sociedade*, *25*(Journal Article). https://doi.org/10.1590/1809-4422asoc20200068r1vu2022l3oa

Damari, B., Vosoogh-Moghaddam, A., & Riazi-Isfahani, S. (2018). Implementing health impact assessment at national level: An experience in Iran. *Iranian Journal of Public Health*, *47*(2), 246–255. https://go.exlibris.link/XVYpPW91

Del Rio, M., Hargrove, W. L., Tomaka, J., & Korc, M. (2017). Transportation Matters: A Health Impact Assessment in Rural New Mexico. *International Journal of Environmental Research and Public Health*, *14*(6). https://doi.org/10.3390/ijerph14060629

Fakhri, A., & Harris, P. (2021). Internationally validating a conceptual framework for health impact assessment. *International Archives of Health Sciences*, *8*(4), 231–236. https://doi.org/10.4103/iahs.iahs_42_21

Fakhri, A., Harris, P., & Maleki, M. (2015). Proposing a framework for Health Impact Assessment in Iran. *BMC Public Health*, *15*(1), 1–7. https://doi.org/10.1186/s12889-015-1698-1

Fischer, T. B., Chang, M., & Muthoora, T. (2024). Health impact assessment in two planning projects in England: Reflections on normative effectiveness. *BMC Public Health*, *24*(1), 2819. https://doi.org/10.1186/s12889-024-20203-7

Gamache, S., Diallo, T., & Lebel, A. (2022). The use of health impact assessments performed in Quebec City (Canada) – 2013–2019: Stakeholders and participants’ appreciation. *Environmental Impact Assessment Review*, *92*(Journal Article), 106693. https://doi.org/10.1016/j.eiar.2021.106693

Gamache, S., Lebel, A., Diallo, T. A., & Shankardass, K. (2020). The elaboration of an intersectoral partnership to perform health impact assessment in urban planning: The experience of quebec city (canada). *International Journal of Environmental Research and Public Health*, *17*(20), 1–15. https://doi.org/10.3390/ijerph17207556

Goff, N., Wyss, K., Wendel, A., & Jarris, P. (2016). Implementing Health Impact Assessment Programs in State Health Agencies: Lessons Learned From Pilot Programs, 2009-2011. *Journal of Public Health Management and Practice : JPHMP*, *22*(6), E8–E13. https://doi.org/10.1097/PHH.0000000000000392

Green, L., Ashton, K., Edmonds, N., & Azam, S. (2020). Process, Practice and Progress: A Case Study of the Health Impact Assessment (HIA) of Brexit in Wales. *International Journal of Environmental Research and Public Health*, *17*(18), 1–14. https://doi.org/10.3390/ijerph17186652

Green, L., Gray, B. J., & Ashton, K. (2020). Using health impact assessments to implement the sustainable development goals in practice: A case study in Wales. *Impact Assessment and Project Appraisal*, *38*(3), 214–224. https://doi.org/10.1080/14615517.2019.1678968

Haigh, F., Baum, F., Dannenberg, A. L., Harris, M. F., Harris-Roxas, B., Keleher, H., Kemp, L., Morgan, R., Chok, H. N., Spickett, J., & Harris, E. (2013). The effectiveness of health impact assessment in influencing decision-making in Australia and New Zealand 2005-2009. *BMC Public Health*, *13*(1), 1188–1188. https://doi.org/10.1186/1471-2458-13-1188

Haigh, F., Harris, E., Harris-Roxas, B., Baum, F., Dannenberg, A. L., Harris, M. F., Keleher, H., Kemp, L., Morgan, R., Chok, H. N. G., & Spickett, J. (2015). What makes health impact assessments successful? Factors contributing to effectiveness in Australia and New Zealand. *BMC Public Health*, *15*(1), 1009–1009. https://doi.org/10.1186/s12889-015-2319-8

Harris-Roxas, B., Haigh, F., Travaglia, J., & Kemp, L. (2014). Evaluating the impact of equity focused health impact assessment on health service planning: Three case studies. *BMC Health Services Research*, *14*(1), 371–371. https://doi.org/10.1186/1472-6963-14-371

Ison, E. (2013). Health Impact Assessment in a Network of European Cities. *Journal of Urban Health*, *90*(Suppl 1), 105–115. https://doi.org/10.1007/s11524-011-9644-8

Jabot, F., & Rivadeneyra-Sicilia, A. (2022). Health impact assessment institutionalisation in France: State of the art, challenges and perspectives. *IMPACT ASSESSMENT AND PROJECT APPRAISAL*, *40*(3), 179–190. https://doi.org/10.1080/14615517.2021.2012011

Jabot, F., Tremblay, E., Rivadeneyra, A., Diallo, T. A., & Lapointe, G. (2020). A comparative analysis of health impact assessment implementation models in the regions of montérégie (Québec, canada) and nouvelle-aquitaine (france). *International Journal of Environmental Research and Public Health*, *17*(18), 1–18. Scopus. https://doi.org/10.3390/ijerph17186558

Kögel, C. C., Peña, T. R., Sánchez, I., Tobella, M., López, J. A., Espot, F. G., Claramunt, F. P., Rabal, G., & Viana, A. G. (2020). Health impact assessment (HIA) of a fluvial environment recovery project in a medium-sized Spanish Town. *International Journal of Environmental Research and Public Health*, *17*(5), 1484. https://doi.org/10.3390/ijerph17051484

Kraemer, S. & Gulis, G. (2014). How do experts define relevance criteria when initiating Health Impact Assessments of national policies? *Scandinavian Journal of Public Health*, *42*(1), 18–24. https://doi.org/10.1177/1403494813504254

Kræmer, S. Johnsdatter, R., Theilgaard Nikolajsen, L and Gulis, G. (2014). Implementation of health impact assessment in Danish municipal context. *Central European Journal of Public Health*, *22*(4), Article 4. https://doi.org/10.21101/cejph.a3943

Linzalone, N., Ballarini, A., Piccinelli, C., Viliani, F., & Bianchi, F. (2018). Institutionalizing Health Impact Assessment: A consultation with experts on the barriers and facilitators to implementing HIA in Italy. *Journal of Environmental Management*, *218*, 95–102. https://doi.org/10.1016/j.jenvman.2018.04.037

Linzalone, N., Coi, A., Lauriola, P., Luise, D., Pedone, A., Romizi, R., Sallese, D., Bianchi, F., Santoro, M., Minichilli, F., Maurello, M. T., Scaringi, M., Zuppiroli, M. E., HIA21 Project Working Group, & HIA21 Project Working Grp. (2017). Participatory health impact assessment used to support decision-making in waste management planning: A replicable experience from Italy. *Waste Management (Elmsford)*, *59*(Journal Article), 557–566. https://doi.org/10.1016/j.wasman.2016.09.035

Liu, X., Liu, Y., Xu, Y., Song, L., Huang, Z., Zhu, X., & Zhang, M. (2023). Current status and influencing factors of policy identification in health impact assessment: A case study of Zhejiang Province. *Health Research Policy and Systems*, *21*(1), 118. https://doi.org/10.1186/s12961-023-01064-9

Marincova, L., Loosova, J., & Valenta, V. (2020). Experiences and needs of Licences Health Risk Assessors using Health Impact Assessment in the Czech Republic. *Central European Journal of Public Health*, *28*(2), 108–113. https://doi.org/10.21101/cejph.a5833

Mattig, T., Cantoreggi, N., Simos, J., Kruit, C. F., & Christie, D. P. T. H. (2017). HIA in Switzerland: Strategies for achieving Health in All Policies. *Health Promotion International*, *32*(1), 149–156. https://doi.org/10.1093/heapro/dav087

Morteruel, M., Bacigalupe, A., Aldasoro, E., Larrañaga, I., & Serrano, E. (2020). Health impact assessments in Spain: Have they been effective? *International Journal of Environmental Research and Public Health*, *17*(8), Article 8. https://doi.org/10.3390/ijerph17082959

Movia, M., Macher, S., Antony, G., Zeuschner, V., Wamprechtsamer, G., Delle Grazie, J., Simi, H., & Fuchs-Neuhold, B. (2022). Health Impact Assessment (HIA) of a Daily Physical Activity Unit in Schools: Focus on Children and Adolescents in Austria Up to the 8th Grade. *International Journal of Environmental Research and Public Health*, *19*(11), Article 11. https://doi.org/10.3390/ijerph19116428

Negev, M., Davidovitch, N., Garb, Y., & Tal, A. (2013). Stakeholder participation in health impact assessment: A multicultural approach. *Environmental Impact Assessment Review*, *43*(Journal Article), 112–120. https://doi.org/10.1016/j.eiar.2013.06.002

O’Mullane, M. (2014). Implementing the legal provisions for HIA in Slovakia: An exploration of practitioner perspectives. *Health Policy*, *117*(1), 112–119. Scopus. https://doi.org/10.1016/j.healthpol.2014.03.005

Pradyumna, A., Farnham, A., Utzinger, J., & Winkler, M. S. (2021). Health impact assessment of a watershed development project in southern India: A case study. *IMPACT ASSESSMENT AND PROJECT APPRAISAL*, *39*(2), 118–126. https://doi.org/10.1080/14615517.2020.1863119

Pursell, L., & Kearns, N. (2013). Impacts of an HIA on inter-agency and inter-sectoral partnerships and community participation: Lessons from a local level HIA in the Republic of Ireland. *Health Promotion International*, *28*(4), 522–532. https://doi.org/10.1093/heapro/das032

Quin, M., Carmichael, L., & Hopper, C. (2023). Implementing Health Impact Assessment policy on infrastructure development in the London Borough of Tower Hamlets. *Cities and Health*, *7*(3), 303–311. Scopus. https://doi.org/10.1080/23748834.2022.2148843

Ramirez-Rubio, O., Daher, C., Fanjul, G., Gascon, M., Mueller, N., Pajin, L., Plasencia, A., Rojas-Rueda, D., Thondoo, M., & Nieuwenhuijsen, M. J. (2019). Urban health: An example of a ‘health in all policies’ approach in the context of SDGs implementation. *Globalization and Health*, *15*(1), 87–87. https://doi.org/10.1186/s12992-019-0529-z

Roué-Le Gall, A., & Jabot, F. (2017). Health impact assessment on urban development projects in France: Finding pathways to fit practice to context. *Global Health Promotion*, *24*(2), 25–34. https://doi.org/10.1177/1757975916675577

Sheffield, P., Rowe, M., Agu, D., Rodríguez, L., & Avilés, K. (2014). Health Impact Assessments for Environmental Restoration: The Case of Caño Martín Peña. *Annals of Global Health*, *80*(4), 296–302. https://doi.org/10.1016/j.aogh.2014.07.001

Thondoo, M., De Vries, D. H., Rojas-Rueda, D., Ramkalam, Y. D., Verlinghieri, E., Gupta, J., & Nieuwenhuijsen, M. J. (2020). Framework for Participatory Quantitative Health Impact Assessment in Low- and Middle-Income Countries. *International Journal of Environmental Research and Public Health*, *17*(20), 1–20. https://doi.org/10.3390/ijerph17207688

Thondoo, M., Goel, R., Tatah, L., Naraynen, N., Woodcock, J., & Nieuwenhuijsen, M. (2022). The Built Environment and Health in Low- and Middle-Income Countries: A Review on Quantitative Health Impact Assessments. *Current Environmental Health Reports*, *9*(1), 90–103. https://doi.org/10.1007/s40572-021-00324-6

Thondoo, M., Mueller, N., Rojas-Rueda, D., de Vries, D., Gupta, J., & Nieuwenhuijsen, M. J. (2020a). Participatory quantitative health impact assessment of urban transport planning: A case study from Eastern Africa. *Environment International*, *144*, 106027. https://doi.org/10.1016/j.envint.2020.106027

Thondoo, M., Rojas-Rueda, D., Gupta, J., de Vries, D. H., & Nieuwenhuijsen, M. J. (2019). Systematic Literature Review of Health Impact Assessments in Low and Middle-Income Countries. *International Journal of Environmental Research and Public Health*, *16*(11), 2018. https://doi.org/10.3390/ijerph16112018

Walpita, Y. N., & Green, L. (2022). Health Impact Assessment (HIA): A Comparative Case Study of Sri Lanka and Wales: What Can a Developing Country Learn From the Welsh HIA System? *International Journal of Health Services*, *52*(2), 283–291. https://doi.org/10.1177/0020731420941454

Westenhöfer, J., Nouri, E., Reschke, M. L., Seebach, F., & Buchcik, J. (2023). Walkability and urban built environments-a systematic review of health impact assessments (HIA). *BMC Public Health*, *23*(1), 518–518. https://doi.org/10.1186/s12889-023-15394-4
