# Supplementary material for: Routemap for health impact assessment implementation: scoping review using the consolidated framework for implementation research
Source: Health Promot Int. 2025 Jun 30;40(3):daaf080. doi: 10.1093/heapro/daaf080 (PMC12208066; doi:10.1093/heapro/daaf080)
Supplement: daaf080_Supplementary_Data [file daaf080_supplementary_data.zip › SM File 3. Summary overview of studies disaggregated by Group.docx]

# **Summary overview of studies included disaggregated by Groups of studies**

### Table 1: Group 1 (n=27) Articles based on primary data collected from HIA stakeholder (participants, practitioners and experts)

| **Focus of papers using primary data** | **Methods** | **What does it tell us about implementation** | **No. of studies (n=27)** | **Study participants** | **Authors** |
| --- | --- | --- | --- | --- | --- |
| Assessing effectiveness and role of context and process factors, factors associated with effectiveness/success | Qualitative interviews (n=1), mixed methods (n=3), survey (n=1) | Factors influencing implementation | 6 | HIA particpants and practitioners | Morteruel et al. 2020, Haigh et al. 2015, Haigh et al. 2013, Ison 2013, Bourcier et al. 2015; Fischer, Chang, Muthoora, 2024 |
| Knowledge, attitudes, opinions, barriers and challenges, and facilitators of HIA | Qualitative interviews (n=1), mixed methods (n=4), survey (n=3) | Factors influencing implementation | 9 | HIA particpants, practitioners, HIA policy developers and executioners (Quin et al.,), current and potential HIA users (Linzalone et al.,, Walpita and Green) | Kraemer et al. 2014, Marincova, Loosova, and Valenta 2020, Kraemer and Gulis 2014, Quin, Carmichael, and Hopper 2023, Linlazone et al. 2018, O Mullane 2014, Fakhri et al. 2021, Walpita and Green 2022: Liu et al. 2023 |
| Components of participatory quantitative model influencing implementation | Mixed methods (n=1) | Considerations throughout the six step HIA | 1 | HIA participants | Thondoo et al. 2020a |
| Models and frameworks for HIA implementation | Mixed methods (n=1), qualitative interviews and focus groups (n=1), qualitative interview (n=1) | Factors influencing implementation/considerations | 3 | HIA participants and practitioners and those likely to be involved in future HIAs | Jabot et al. 2020, Damari, Vosoogh-Moghaddam, and Riazi-Isfahani 2018, Fakhri, Harris, and Maleki 2015 |
| Process and HIA outcomes | Direct observation and minute taking | Implementation process, participants perceptions and factors influencing implementation | 1 | HIA particpants and practitioners | Gamache et al. 2020 |
| Contribution of specialist to HIA implementation process | Qualitative interviews | Considerations to strengthen HIA process and methods | 1 | Experts from higher education institutes | Busato and Grisotti 2022. |
| HIA practise and its ability to progress a HIaP approach | Mixed methods | Barriers and facilitators and Considerations to strengthen process and institutionalisation | 1 | HIA ‘key informers’ (practitioners/experts) | Mattig et al. 2017 |
| HIA process, practises, influences and impacts at city level | Mixed methods (n=2), qualitative interviews (n=1) | Factors influencing implementation | 3 | HIA participants | Gamache 2022, Purcell and Kearns 2013; Buregeya, Loignon, and Brousselle 2020 |
| Implementation at city level and barriers to implementation | surveys | Factors influencing implementation | 1 | HIA practitioners | Berrenson and Tillgren 2017 |
| Evaluating the impact of equity focussed HIA (EFHIA) | Qualitative retrospective case study and interviews | Factors influencing EFHIA implementation | 1 | HIA participants and persons involved in developing the health sector plan | Harris-Roxas et al.2014 |

### Table 2: Group 2 (n=8) Articles based on secondary data

| **Focus of review papers and case studies (secondary data)** | **Methods** | **What does it tell us about implementation** | **Country** | **Authors** |
| --- | --- | --- | --- | --- |
| Walkability and urban environments: systematic review | Systematic review of the literature | Types of HIAs, data sources used, stakeholders, data missing in terms of implementation (who is doing the work) health impacts considered | Multi-country | Westenhofer et al. 2023 |
| HIA in US housing sector | Systematic review of 54 HIAs | Types of decisions examined, types of organizations leading the assessment, process and methods and policy impacts | US | Bever et al. 2021 |
| Built environment and health in low- and middle-income countries: review | Review of 24 HIA case studies | Methods and tools used to support implementation | LMIC | Thondoo et al. 2022 |
| Urban health and HIA as a tool for HiAP | Review of urban HIAs | Opportunities and barriers to the implementation of HIAs in cities across Europe, Africa and Latin-America | Multi-country | Ramirez-Rubio et al.2019 |
| HIA in low and middle income countries: systematic review | Systematic Review of 57 case studies | Factors to improve HIA implementation | LMIC | Thondoo et al.2019 |
| HIA on urban development projects in France | Case study review of four urban HIAs | Ways to adapt HIA implementation to the specific context of urban project development | France | Roué-Le Gall and Jabot 2017 |
| Institutionalizing HIA in France | Case study review of 41 HIAs | Implementation process, challenges and opportunities. | France | Jabot and Rivadeneyra-Sicilia, 2022 |
| Lessons from Pilot HIA in the US | Review of nine HIA programmes | components of initiatives that produced effective HIAs, methods for developing effective HIA programs | US | Goff et al. 2016 |

### Table 3: Group 3, HIA case studies

| **Authors** | **HIA case study: type, country, level and focus** | **Timeframe** | **Objectives of HIA if noted.** | **Stakeholders involved** | **Evidence used** | **Initiated by** | **HIA steps included and steps not explored (if mentioned)** |
| --- | --- | --- | --- | --- | --- | --- | --- |
| (Green et al. 2020) | Wales, advocacy*, Prospective strategic HIA, Brexit. | 6 months | To capture the impacts of Brexit | Core group and working group. No details provided  Strategic Advisory Group: cross-sector and disciplinary, internal and external stakeholders such as PHW, Welsh Government, academia and from a range of backgrounds (environmental public health; health policy, Brexit planning.  Interviews: included representatives from a wide range of identiﬁed aﬀected sectors, including the Welsh NHS Confederation, Nursing and General Practice, WG, Food Standards Agency Wales and the Local Government | A focussed Literature review (Brexit impacts across Wales and UK populations).  Quantitative health intelligence (level of deprivation, demographics, prevalence of health conditions).  Semi structured interviews (n=17) with 25 representatives from 12 organisations.  Workshop (n=14) range of disciplines and agencies including environmental and public health, sustainable development, healthcare services and the housing sectors | Supported by Wales Health Impact Assessment Support Unit (WHIASU) and Public Health Wales (PHW) | Screening, scoping, appraisal, reporting, monitoring  Appraisal of specific Brexit scenarios not explored |
| (Green, Gray, and Ashton 2020)  . | Wales, decision support*, prospective, project HIA, electricity cable connection development | None provided | HIA undertaken to consider the wider impacts of the electricity cable connection development on key stakeholders who could be aﬀected by the preferred route | Steering Group: ‘small multidisciplinary included stakeholders from the local public health team, local authority environmental health, inequalities and wellbeing oﬃcers; the WHIASU leads; consultants who were appointed by the power company in the ﬁelds of health, environmental and social impact and the Project Manager and Director’  Citizens via workshop with other stakeholders | Local health intelligence, community statistics and other quantitative baseline information (such as traﬃc information, noise or vibration and air quality statistics)  A participatory workshop (n=39) was undertaken with a broad range of local stakeholders as participants (i.e. power company, local authority oﬃcers (well-being; environmental health; transport and planning) public health and health services oﬃcers, third sector  representatives, residents) | The Deputy Chief Executive of the originating local authority, with agreement from the energy company, approached the Wales Health Impact Assessment Support Unit (WHIASU) to independently facilitate the HIA | Screening, scoping, appraisal, reporting.  monitoring not reported. |
| (Thondoo et al. 2020a) | Mauritius, advocacy*, prospective, strategic, HIA of transport scenarios | No dates provided | To estimate the health impacts of shifting transport modes via participatory quantitative HIA approach | Stakeholders consulted by expertise to identify context specificity and policy relevance: Social worker, Politician, Journalist, Economic investments, Food services and Sustainable development executive, Public agencies x 4 (Town and planning services, technician: sustainable economic development, Traffic planning, Statistician: land transport), International multilateral organisation (sustainable development), Urban planner, Elected Officials x 2 (Medicine and Health, Land Transport), Consultant (sustainable development) and Expert in Ecosystems.  Citizens via survey. | Interviews (IDIs) with 14 stakeholders  Closed-ended survey questions (n=600 citizens)  2 focus group discussions (FGDs) with the same 14 stakeholders above  Modelling for all-cause mortality using three indicators (Air pollution, Traffic Deaths, Physical Activity)  WHO Survey travel data, survey PA data. existing AP data, existing traffic death data and natural all cause mortality.  Three future transport scenarios built from stakeholder feedback. | Proposed by the first author | screening, scoping, appraisal and reporting.  Monitoring phase excluded due to time constraints |
| (Pradyumna et al. 2021) | Prospective, decision support*, project level HIA on a watershed project in Kolar district, India. | 7 months | To describe the baseline health conditions of the local population;  To identify potential health impacts of the proposed WSD project, including considerations of magnitude and significance; and  To make evidence-based recommendations towards mitigation of potential negative health impacts and promotion of health opportunities | NGO (project proponent supported decision to carry out HIA, was not involved the design or reporting but did collect data for baseline survey), healthcare providers, village residents.  Citizens via health census | Review of proposal documents, desktop literature review, group discussions with local people  Interviews with healthcare providers in project villages;  Health Census of all households in the projects villages (socio-demographic aspects, and knowledge, attitudes and practices related to health, and household health concerns) | Proposed by first author to NGO team managing watershed projects | screening, scoping, appraisal and reporting  monitoring and evaluation not included |
| (Del Rio et al. 2017) | Prospective, advocacy*, strategic HIA of public transport in rural areas, US | 2 years | HIA focussed on direct health impacts related primarily to access to health care and fresh fruits and vegetables (because of their impact on a healthy diet, as well as other conditions conducive to safety (such as road accidents) and healthy living (such as walking to bus stops). | Initial consultation with a local chapter of the Empowerment Congress in Doña Ana County who has already conducted a survey among its members to assess the need for and beneﬁts of public transportation in the area. This survey was used to focus the HIA.  Rural residents and key informants (professionals from a range of sectors including health, education, business or economic development, social services, and environment) | Community survey of resident over the age of 15 from 21 different communities (n=1054).  Bus ridership survey (n=33)  Interviews with 44 key informants via recommendations from local leaders, members of the Empowerment Congress, and key stakeholders.  focus group (n=13) community health workers |  | Screening Scoping, Assessment, Recommendations, Reporting, and Monitoring |
| (Kögel et al. 2020) | A prospective, decision support*, project level, HIA of fluvial environment recovery, Spain | 8 months | To assess potential positive and negative health impacts of the project  To estimate the distribution regarding age, sex, and socioeconomic class of potential health impacts  To elaborate recommendations to propose changes and improvements. | Steering group: the  mayor, environment and public health area councillors, the Public Health Agency of Catalonia (ASPCAT) area manager, and the architect in charge of the project from the AMB.  An evaluation: four technicians from the city council (public health area), the AMB, and the ASPCAT.  Experts  Citizens | Data from Health Report of general practitioners  and the Municipal Health Plan (2013–2018)  An experts’ meeting (n=11) with technical personnel to assess the potential eﬀects of the project. Comprised of six technicians from the city council (areas of culture, environment,  public health, and public transport), the AMB architect in charge of the project, two representatives from the ASPCAT, and two representatives from the provincial government.  Primary health care professionals and the police were interviewed as they could not assist in the meeting  A citizens discussion group (n=16) was formed with the general population (convenience sample) and associations.  Literature to verify potential health impacts identified (WHO report on green spaces and health) | City Council of Sant Andreu with methodological support from the Public Health Agency of Catalonia | Screening Scoping, appraisal, Recommendations, Reporting, and Monitoring & evaluation |
| (Linzalone et al. 2017) | Participatory, strategic, decision support* HIA, Italy, waste management planning. | 3 years | To develop a participatory methodology. Paper focussed on describing the democratic path of participation and the results of the HIA21 project. | General public was involved  to provide input in the definition of the methodological project  advancements.  Focus groups participants were asked to cooperate in the HIA steps of the scoping and assessment phases and in the definition of the monitoring plan  Focus group invitees: municipal and provincial administrators and decision makers, departments from environmental and health agencies, professional corporations, civil associations for health and social promotion, business enterprises, citizen leagues, general practitioners. | The environmental agency supplied information re. the industrial activities in the area, maps of pollution levels, and previous environmental studies;  The local health department provided technical support, historical and current health data  The association of doctors for the environment provided training and expertise on the relationship between the environment and health, and supported the improvement in knowledge and public awareness.  Two questionnaires concerning risk perception (n=326) filled out by majority of participants  Retrospective cohort study considering the residents living in the area over previous decade | The municipal council of Arezzo and academics | Screening, Scoping, Assessment, reporting recommendations, monitoring |
| (Sheffield et al. 2014) | Prospective , decision support*, strategic HIA on environmental restoration and community development plan (dredging a channel and various infrastructure interventions), Puerto Rico | 15 months | Limited the scope of the HIA to address three key elements of the proposed plan: 1) dredging and debris removal, 2) road, sewer, and storm water drainage infrastructure changes including development of the waterfront Paseo, and 3) housing changes including resident relocation due to the necessary demolition of homes located along the channel  Scoping training 40 participants that included representatives from government agencies, multiple public health academic institutions, non-profit advocacy organizations, and community member | Two groups established at screening stage to guide HIA: a Community Advisory Committee (local community residents) Steering Committee (health and urban planning professionals) | Health data from previous studies in the community  A literature review of epidemiologic data (links between environmental exposures and health outcomes)  Interviews with school administrators regarding flooding impacts  Department of Health dengue surveillance data  Focus groups (n=5) with community members (explored current environmental conditions and health concerns, vulnerable groups, and perceptions of how the proposed plan would affect the community) | Puerto Rico Highway and Transportation Authority | screening, scoping, assessment, recommendations, reporting, and monitoring and evaluation |
| (Negev et al. 2013) | Advocacy*, prospective strategic HIA on the metropolitan plan of the city of Be'er Sheva in Southern Israel | No dates provided | To develop a model for multicultural HIA Model. The health issue scoped by the stakeholders in the HIA is related to land uses in the vicinity of the national hazardous industry and hazardous waste site | Three meetings of the stakeholders' HIA steering committee (the scoping stage of the HIA used diverse methods to maximize participation including general discussions, heterogeneous focus groups and anonymous voting)  The steering committee represented the public sector (Ministry of Health, Ministry of Environmental Protection, Planning Authority, and local authorities, including the industrial authority), the general public (Jewish and Bedouin residents of diverse settlements in the metropolitan area: the city with 200,000 residents, afﬂuent satellite Jewish towns, Bedouin towns and Bedouin villages), academia, the private sector (factories in the Site) and NGOs (local and national, environmental and social).  30 members, including approximately 1–2 representatives of each governmental institution, about 8 members of the public representing the diverse communities, 2 members of academia, 3 private sector representatives, and 4 NGO workers. | Risk assessment carried out by an independent expert. (focused on acute health impacts in the case of a toxic event)  Review literature on health impacts (including culturally related impacts)  Local evidence gathered in steering committee meetings.  Interviews with 27 stakeholders (Ministry of Health, Ministry of Environmental Protection, Planning Authority, and local authorities, including the industrial authority, the general public (Jewish and Bedouin residents of diverse settlements in the metropolitan area, academia, the private sector (factories in the Site) and NGOs (local and national, environmental and social). Excellent paper but focussed on more about the challenges of multicultural participation. | The Ben Gurion University's Center for Health Policy Research promoted an HIA of the Be'er Sheva metropolitan plan independent of this research, and later conducted a participatory HIA in collaboration with the ﬁrst author of this paper | Screening, scoping, appraisal, reporting and evaluation. |
| (Movia et al. 2022) | Prospective, decision support*, project level, HIA on physical activity units in Austrian schools | 6 months. Literature search began in April 2021 and report produced in September 2021 | Health Impact Assessment on the introduction of the daily physical activity unit for school children in Austria from the ﬁrst to the eighth grade. Focus on four determinants of health   1. individual health. 2. social and community-related networks, impact further target groups. 3. 3. living and working conditions (school-setting). 4. 4. general conditions of system, socio-economic, and environmental factors. | No information provided on board.  Focus group participants included: Principals, school administration, school management (n=10) parents/legal guardians – inclusive of statements form children (n=8), special inspectors for physical activity (n=10)  Teachers (n=8)  Appraisal workshop (x2) participants (‘members of the advisory board, parent representatives, experts from the ﬁelds of health, education, and sports, and other relevant stakeholders’) | Literature review (articles that include ﬁndings on physical activity in relation to the individual health of children and adolescents; social and community-related networks, impact on further target groups; living and working conditions (school-setting); and general conditions of system, socio-economic, and environmental factors. Specific focus on vulnerable groups  Online survey (n=712) of teachers, principal, admin and parents  Four focus groups (n=4) with teachers, inspectors for physical activity and sport, parents and principals  Appraisal workshops x 2 (three hours each) | The Federal Ministry of Education, Science and Research and the Federal Ministry of Arts, Culture, Public Service and Sports commissioned an interdisciplinarity project working group to develop a concept for the “Daily Physical Activity Unit” in schools.  HIA conducted on behalf of the Federal Ministry of Social Affairs, Health, Care and Consumer  Protection, FH JOANNEUM—University of Applied Sciences, Institute of Health and Tourism Management, | Screening and scoping, appraisal, and formulation of recommendations.  Monitoring and evaluation excluded |

### Table 4. Basis of reported findings: number of countries and HIAs explored and methods used across studies

| **Article group** | **Findings based on:** | **No. of studies** | **Qualitative: Interviews, focus groups, ethnographic approaches** | **Surveys or questionnaires** | **Secondary analysis/reflective/ no study participants** | **Mixed methods** |
| --- | --- | --- | --- | --- | --- | --- |
| Group 1 | **One country** and exploring **more than one HIA process** | 18 | 7 | 3 | - | 8 |
| Group 1 | **One country** and **focused on a specific HIA process** | 2 | 2 |  | - |  |
| Group 1 | **Multiple countries** (more than one) and **exploring more than one HIA process** | 5 | 1 | 2 | - | 2 |
| Group 1 | Exploring **models of HIA implementation** (pre or post) | 2 |  |  |  | 2 |
| Group 2 | **Review papers** focussed on **single country** (no primary data collection) | 4 | - | - | 4 | - |
| Group 2 | **Review papers** focussed on **multiple countries** (no primary data collection) | 4 | - | - | 4 | - |
| Group 3 | **HIA case study** describing how the HIA was carried out | 10 |  |  |  | 10 |

*Decision support HIA are HIAs conducted voluntarily by or with the agreement of organisations responsible for a proposal (Harris-Roxas and Harris 2011) (Harris-Roxas & Harris, 2011).

*Advocacy HIAs are HIA by conducted by organisations or groups who are not responsible for the proposal (Harris-Roxas & Harris, 2011).

**Reference List**

Berensson, K., & Tillgren, P. (2017). Health impact assessment (HIA) of political proposals at the local level: Successful introduction, but what has happened 15 years later? *Global Health Promotion*, *24*(2), 43–51. https://doi.org/10.1177/1757975916683386

Bever, E., Arnold, K. T., Lindberg, R., Dannenberg, A. L., Morley, R., Breysse, J., & Pollack Porter, K. M. (2021). Use of health impact assessments in the housing sector to promote health in the United States, 2002–2016. *Journal of Housing and the Built Environment*, *36*(3), 1277–1297. https://doi.org/10.1007/s10901-020-09795-9

Bourcier, E., Charbonneau, D., Cahill, C., & Dannenberg, A. L. (2015). An Evaluation of Health Impact Assessments in the United States, 2011–2014. *Preventing Chronic Disease*, *12*, 140376. https://doi.org/10.5888/pcd12.140376

Buregeya, J. M., Loignon, C., & Brousselle, A. (2020). Contribution analysis to analyze the effects of the health impact assessment at the local level: A case of urban revitalization. *Evaluation and Program Planning*, *79*, 101746–15. https://doi.org/10.1016/j.evalprogplan.2019.101746

Busato, M. A., & Grisotti, M. (2022). Health impact assessment in the process of implementation of hydroelectric plants: Methodological contributions. *Ambiente & Sociedade*, *25*(Journal Article). https://doi.org/10.1590/1809-4422asoc20200068r1vu2022l3oa

Damari, B., Vosoogh-Moghaddam, A., & Riazi-Isfahani, S. (2018). Implementing health impact assessment at national level: An experience in Iran. *Iranian Journal of Public Health*, *47*(2), 246–255. https://go.exlibris.link/XVYpPW91

Del Rio, M., Hargrove, W. L., Tomaka, J., & Korc, M. (2017). Transportation Matters: A Health Impact Assessment in Rural New Mexico. *International Journal of Environmental Research and Public Health*, *14*(6). https://doi.org/10.3390/ijerph14060629

Fakhri, A., & Harris, P. (2021). Internationally validating a conceptual framework for health impact assessment. *International Archives of Health Sciences*, *8*(4), 231–236. https://doi.org/10.4103/iahs.iahs_42_21

Fakhri, A., Harris, P., & Maleki, M. (2015). Proposing a framework for Health Impact Assessment in Iran. *BMC Public Health*, *15*(1), 1–7. https://doi.org/10.1186/s12889-015-1698-1

Fischer, T. B., Chang, M., & Muthoora, T. (2024). Health impact assessment in two planning projects in England: Reflections on normative effectiveness. *BMC Public Health*, *24*(1), 2819. https://doi.org/10.1186/s12889-024-20203-7

Gamache, S., Diallo, T., & Lebel, A. (2022). The use of health impact assessments performed in Quebec City (Canada) – 2013–2019: Stakeholders and participants’ appreciation. *Environmental Impact Assessment Review*, *92*(Journal Article), 106693. https://doi.org/10.1016/j.eiar.2021.106693

Gamache, S., Lebel, A., Diallo, T. A., & Shankardass, K. (2020). The elaboration of an intersectoral partnership to perform health impact assessment in urban planning: The experience of quebec city (canada). *International Journal of Environmental Research and Public Health*, *17*(20), 1–15. https://doi.org/10.3390/ijerph17207556

Goff, N., Wyss, K., Wendel, A., & Jarris, P. (2016). Implementing Health Impact Assessment Programs in State Health Agencies: Lessons Learned From Pilot Programs, 2009-2011. *Journal of Public Health Management and Practice : JPHMP*, *22*(6), E8–E13. https://doi.org/10.1097/PHH.0000000000000392

Green, L., Ashton, K., Edmonds, N., & Azam, S. (2020). Process, Practice and Progress: A Case Study of the Health Impact Assessment (HIA) of Brexit in Wales. *International Journal of Environmental Research and Public Health*, *17*(18), 1–14. https://doi.org/10.3390/ijerph17186652

Green, L., Gray, B. J., & Ashton, K. (2020). Using health impact assessments to implement the sustainable development goals in practice: A case study in Wales. *Impact Assessment and Project Appraisal*, *38*(3), 214–224. https://doi.org/10.1080/14615517.2019.1678968

Haigh, F., Baum, F., Dannenberg, A. L., Harris, M. F., Harris-Roxas, B., Keleher, H., Kemp, L., Morgan, R., Chok, H. N., Spickett, J., & Harris, E. (2013). The effectiveness of health impact assessment in influencing decision-making in Australia and New Zealand 2005-2009. *BMC Public Health*, *13*(1), 1188–1188. https://doi.org/10.1186/1471-2458-13-1188

Haigh, F., Harris, E., Harris-Roxas, B., Baum, F., Dannenberg, A. L., Harris, M. F., Keleher, H., Kemp, L., Morgan, R., Chok, H. N. G., & Spickett, J. (2015). What makes health impact assessments successful? Factors contributing to effectiveness in Australia and New Zealand. *BMC Public Health*, *15*(1), 1009–1009. https://doi.org/10.1186/s12889-015-2319-8

Harris-Roxas, B., Haigh, F., Travaglia, J., & Kemp, L. (2014). Evaluating the impact of equity focused health impact assessment on health service planning: Three case studies. *BMC Health Services Research*, *14*(1), 371–371. https://doi.org/10.1186/1472-6963-14-371

Ison, E. (2013). Health Impact Assessment in a Network of European Cities. *Journal of Urban Health*, *90*(Suppl 1), 105–115. https://doi.org/10.1007/s11524-011-9644-8

Jabot, F., & Rivadeneyra-Sicilia, A. (2022). Health impact assessment institutionalisation in France: State of the art, challenges and perspectives. *IMPACT ASSESSMENT AND PROJECT APPRAISAL*, *40*(3), 179–190. https://doi.org/10.1080/14615517.2021.2012011

Jabot, F., Tremblay, E., Rivadeneyra, A., Diallo, T. A., & Lapointe, G. (2020). A comparative analysis of health impact assessment implementation models in the regions of montérégie (Québec, canada) and nouvelle-aquitaine (france). *International Journal of Environmental Research and Public Health*, *17*(18), 1–18. Scopus. https://doi.org/10.3390/ijerph17186558

Kögel, C. C., Peña, T. R., Sánchez, I., Tobella, M., López, J. A., Espot, F. G., Claramunt, F. P., Rabal, G., & Viana, A. G. (2020). Health impact assessment (HIA) of a fluvial environment recovery project in a medium-sized Spanish Town. *International Journal of Environmental Research and Public Health*, *17*(5), 1484. https://doi.org/10.3390/ijerph17051484

Kraemer, S. R. J., & Gulis, G. (2014). How do experts define relevance criteria when initiating Health Impact Assessments of national policies? *Scandinavian Journal of Public Health*, *42*(1), 18–24. https://doi.org/10.1177/1403494813504254

Kræmer, S. R. J., Nikolajsen, L. T., & Gulis, G. (2014). Implementation of health impact assessment in Danish municipal context. *Central European Journal of Public Health*, *22*(4), Article 4. https://doi.org/10.21101/cejph.a3943

Linzalone, N., Ballarini, A., Piccinelli, C., Viliani, F., & Bianchi, F. (2018). Institutionalizing Health Impact Assessment: A consultation with experts on the barriers and facilitators to implementing HIA in Italy. *Journal of Environmental Management*, *218*, 95–102. https://doi.org/10.1016/j.jenvman.2018.04.037

Linzalone, N., Coi, A., Lauriola, P., Luise, D., Pedone, A., Romizi, R., Sallese, D., Bianchi, F., Santoro, M., Minichilli, F., Maurello, M. T., Scaringi, M., Zuppiroli, M. E., HIA21 Project Working Group, & HIA21 Project Working Grp. (2017). Participatory health impact assessment used to support decision-making in waste management planning: A replicable experience from Italy. *Waste Management (Elmsford)*, *59*(Journal Article), 557–566. https://doi.org/10.1016/j.wasman.2016.09.035

Liu, X., Liu, Y., Xu, Y., Song, L., Huang, Z., Zhu, X., & Zhang, M. (2023). Current status and influencing factors of policy identification in health impact assessment: A case study of Zhejiang Province. *Health Research Policy and Systems*, *21*(1), 118. https://doi.org/10.1186/s12961-023-01064-9

Marincová, L., Loosova, J., & Valenta, V. (2020). Experiences and needs of Licences Health Risk Assessors using Health Impact Assessment in the Czech Republic. *Central European Journal of Public Health*, *28*(2), 108–113. https://doi.org/10.21101/cejph.a5833

Mattig, T., Cantoreggi, N., Simos, J., Kruit, C. F., & Christie, D. P. T. H. (2017). HIA in Switzerland: Strategies for achieving Health in All Policies. *Health Promotion International*, *32*(1), 149–156. https://doi.org/10.1093/heapro/dav087

Morteruel, M., Bacigalupe, A., Aldasoro, E., Larrañaga, I., & Serrano, E. (2020). Health impact assessments in Spain: Have they been effective? *International Journal of Environmental Research and Public Health*, *17*(8), Article 8. https://doi.org/10.3390/ijerph17082959

Movia, M., Macher, S., Antony, G., Zeuschner, V., Wamprechtsamer, G., Delle Grazie, J., Simi, H., & Fuchs-Neuhold, B. (2022). Health Impact Assessment (HIA) of a Daily Physical Activity Unit in Schools: Focus on Children and Adolescents in Austria Up to the 8th Grade. *International Journal of Environmental Research and Public Health*, *19*(11), Article 11. https://doi.org/10.3390/ijerph19116428

Negev, M., Davidovitch, N., Garb, Y., & Tal, A. (2013). Stakeholder participation in health impact assessment: A multicultural approach. *Environmental Impact Assessment Review*, *43*(Journal Article), 112–120. https://doi.org/10.1016/j.eiar.2013.06.002

O’Mullane, M. (2014). Implementing the legal provisions for HIA in Slovakia: An exploration of practitioner perspectives. *Health Policy*, *117*(1), 112–119. Scopus. https://doi.org/10.1016/j.healthpol.2014.03.005

Pradyumna, A., Farnham, A., Utzinger, J., & Winkler, M. S. (2021). Health impact assessment of a watershed development project in southern India: A case study. *IMPACT ASSESSMENT AND PROJECT APPRAISAL*, *39*(2), 118–126. https://doi.org/10.1080/14615517.2020.1863119

Pursell, L., & Kearns, N. (2013). Impacts of an HIA on inter-agency and inter-sectoral partnerships and community participation: Lessons from a local level HIA in the Republic of Ireland. *Health Promotion International*, *28*(4), 522–532. https://doi.org/10.1093/heapro/das032

Quin, M., Carmichael, L., & Hopper, C. (2023). Implementing Health Impact Assessment policy on infrastructure development in the London Borough of Tower Hamlets. *Cities and Health*, *7*(3), 303–311. Scopus. https://doi.org/10.1080/23748834.2022.2148843

Ramirez-Rubio, O., Daher, C., Fanjul, G., Gascon, M., Mueller, N., Pajin, L., Plasencia, A., Rojas-Rueda, D., Thondoo, M., & Nieuwenhuijsen, M. J. (2019). Urban health: An example of a ‘health in all policies’ approach in the context of SDGs implementation. *Globalization and Health*, *15*(1), 87–87. https://doi.org/10.1186/s12992-019-0529-z

Roué-Le Gall, A., & Jabot, F. (2017). Health impact assessment on urban development projects in France: Finding pathways to fit practice to context. *Global Health Promotion*, *24*(2), 25–34. https://doi.org/10.1177/1757975916675577

Sheffield, P., Rowe, M., Agu, D., Rodríguez, L., & Avilés, K. (2014). Health Impact Assessments for Environmental Restoration: The Case of Caño Martín Peña. *Annals of Global Health*, *80*(4), 296–302. https://doi.org/10.1016/j.aogh.2014.07.001

Thondoo, M., De Vries, D. H., Rojas-Rueda, D., Ramkalam, Y. D., Verlinghieri, E., Gupta, J., & Nieuwenhuijsen, M. J. (2020). Framework for Participatory Quantitative Health Impact Assessment in Low- and Middle-Income Countries. *International Journal of Environmental Research and Public Health*, *17*(20), 1–20. https://doi.org/10.3390/ijerph17207688

Thondoo, M., Goel, R., Tatah, L., Naraynen, N., Woodcock, J., & Nieuwenhuijsen, M. (2022). The Built Environment and Health in Low- and Middle-Income Countries: A Review on Quantitative Health Impact Assessments. *Current Environmental Health Reports*, *9*(1), 90–103. https://doi.org/10.1007/s40572-021-00324-6

Thondoo, M., Mueller, N., Rojas-Rueda, D., de Vries, D., Gupta, J., & Nieuwenhuijsen, M. J. (2020a). Participatory quantitative health impact assessment of urban transport planning: A case study from Eastern Africa. *Environment International*, *144*, 106027. https://doi.org/10.1016/j.envint.2020.106027

Thondoo, M., Rojas-Rueda, D., Gupta, J., de Vries, D. H., & Nieuwenhuijsen, M. J. (2019). Systematic Literature Review of Health Impact Assessments in Low and Middle-Income Countries. *International Journal of Environmental Research and Public Health*, *16*(11), 2018. https://doi.org/10.3390/ijerph16112018

Walpita, Y. N., & Green, L. (2022). Health Impact Assessment (HIA): A Comparative Case Study of Sri Lanka and Wales: What Can a Developing Country Learn From the Welsh HIA System? *International Journal of Health Services*, *52*(2), 283–291. https://doi.org/10.1177/0020731420941454

Westenhöfer, J., Nouri, E., Reschke, M. L., Seebach, F., & Buchcik, J. (2023). Walkability and urban built environments-a systematic review of health impact assessments (HIA). *BMC Public Health*, *23*(1), 518–518. https://doi.org/10.1186/s12889-023-15394-4
