# Supplementary material for: Routemap for health impact assessment implementation: scoping review using the consolidated framework for implementation research
Source: Health Promot Int. 2025 Jun 30;40(3):daaf080. doi: 10.1093/heapro/daaf080 (PMC12208066; doi:10.1093/heapro/daaf080)
Supplement: daaf080_Supplementary_Data [file daaf080_supplementary_data.zip › SM File 4. Barriers & Facilitators by groups.docx]

# **Overview of HIA implementation barriers and facilitators assigned to CFIR domain and constructs disaggregated by Group 1, 2 and 3.**

## Table 1: HIA implementation challenges (Group one papers, N=27)

| **Challenges to implementation identified from group one papers (n=27)** | | | |
| --- | --- | --- | --- |
| **Potential barriers** | **CFIR Domain** | **Construct: subconstruct** | **Source** |
| Lack of evidence on effectiveness, based on HIA evaluations | Innovation | Evidence base: strength of evidence & quality | Kraemer, Nikolajsen, and Gulis 2014, Fischer, Chang and Muthoora, 2024) |
| Concerns about increased costs and bureaucracy associated with HIA implementation | Innovation | Cost | Quin, Carmichael, and Hopper 2023, Mattig et al. 2017 |
| Lack of accepted screening and evaluation tool leading to subjective judgement in screening | Innovation | Evidence base: strength of evidence & quality | Kraemer, Nikolajsen, and Gulis 2014 |
| Combining scientific literature and stakeholder perspectives can lead to methodological problem that can undermine the validity of assessment | Innovation | Evidence base: Strength of evidence & quality | Kraemer, Nikolajsen, and Gulis 2014 |
| Difficulties in implementing strategic HIAs (ie. Policy HIAs less practical than project HIAs) | Innovation | Complexity | Damari, Vosoogh-Moghaddam, and Riazi-Isfahani 2018 |
| Deciding where to start and who are those affected | Innovation | Complexity | Busato and Grisotti 2022, |
| Overlap with additional impact assessment (EIA, CIAs, SIAs), idea that HIA is a clone of EIA, and competing demands with other IAs | Innovation | Relative advantage | Damari, Vosoogh-Moghaddam, and Riazi-Isfahani 2018, Ison 2013, Mattig et al. 2017 |
| Reservations about the value of HIAs / Lack of appreciation of what HIA can offer | Innovation | Relative advantage | Quin, Carmichael, and Hopper 2023. Ison 2013 |
| Perceived complexity of HIA/difficult to implement in the ‘real world’ | Innovation | Complexity | Ison 2013, Kraemer and Gulis 2014 |
| Lack of HIA regulations, statutory or policy requirement, legal basis for implementation | Outer setting | Policies and Laws | Damari et al., 2018, Quin, Carmichael, and Hopper 2023, Ison 2013 |
| Pace of decision making and political administration (ie. keeping up with and responding to fast pace of political process) | Outer setting | Local conditions | Bourcier et al. 2015 |
| Lack of political support | Outer setting | Policies and Laws | Ison 2013, Morteruel et al. 2020 |
| Lack of professional support | Outer setting | Partnerships and Connection | Ison 2013 |
| Socio-political and economic context of economic crisis | Outer setting | Critical incidents | Morteruel et al. 2020; Liu, Liu, Xu et al. 2023 |
| Tensions between citizens institutions responsible for decision making | Outer setting | Local conditions | Morteruel et al. 2020 |
| Lack of political consensus, divergence among politicians and the public servants in relation to priorities | Outer setting | Local conditions | Kraemer, Nikolajsen, and Gulis 2014 |
| Lack of formal agreement on HIAs | Outer setting | Policies and Laws | Morteruel et al. 2020 |
| Lack of education system/setting that include HIA | Outer setting | Local condition: HIA within educational settings | Marincová, Loosova and Valenta 2020 |
| Lack of institutional preparedness to operationalise HIA | Outer setting | Local conditions | O’Mullane 2014 |
| ‘Conservative lobbies/pro business organisations’ (re. embedding HIA as HiAP tool in public health law)  The inﬂuence of the private sector in formulation or assessing HIA | Outer setting | External pressure | Mattig et al. 2017, O’Mullane 2014 |
| Cost/resources (financial and human) required to implementing HIA recommendations/assimilating the HIA process after initial implementation | Inner setting | Available resources: funding | Buregeya, Loignon and Brousselle 2020, Kraemer, and Gulis 2014, Ison 2013, Fakhri, Harris, and Maleki 2015, Ison 2013 , Marincová, Loosova and Valenta 2020; Morteruel et al. 2020, O Mullane 2014, Thondoo et al. 2020 |
| Time required for eﬀective acculturation to HIA values and principles, time to ensure public participation and timing of HIA (ie. impacts scope to alter aspects) | Inner setting | Available resources: Time | Jabot et al. 2020, Gamache et al. 2020, Harris-Roxas et al. 2014 |
| Adequate HIA training | Inner setting | Available Resources: Access to Knowledge & information | Damari, Vosoogh-Moghaddam, and Riazi-Isfahani. 2018 |
| Access to data, evidence, tools and methodologies | Inner setting | Available Resources: Access to Knowledge & information | Bourcier et al. 2015, Fakhri, Harris, and Maleki 2015, Kraemer, Nikolajsen, and Gulis 2014, Linzalone et al. 2018, Thondoo et al. 2020a, Kraemer, Nikolajsen, and Gulis 2014 |
| Lack of capacity to carry out HIA (Knowledge, Skills, Resources and Training/experience) | Inner setting | Available Resources | Kraemer, Nikolajsen, and Gulis 2014, Quin, Carmichael, and Hopper 2023, Ison 2013, Liu et al. 2023 |
| Lack of deep understanding of HIA | Inner setting | Available Resources: Access to knowledge & information | Liu et al. 2023 |
| Lack of scientific literature relating to interventions on the social determinants of health (key component of HIA) in a particular country context | Inner setting | Available Resources: Access to Knowledge & information | Morteruel et al. 2020 |
| Lack of information from the National Public Health Authority | Inner setting | Access to knowledge and information | O’ Mullane 2014 |
| Resistance, lack of readiness, cultural barriers or the hierarchical structure of the organisation | Inner setting | Culture | Ison 2013 |
| Lack of capacity to respond to all requests for HIAs | Inner setting | Available resources | Ison 2013 |
| Inexperienced HIA providers | Inner setting | Available Resources: Access to Knowledge & information | Jabot et al. 2020 |
| Low familiarity with health promotion approaches and low support for HIA advocacy | Inner setting | Compatibility | Jabot et al. 2020 |
| Dispersion of responsibilities weakening the leadership required for the execution of an HIA approach. | Inner setting | Structural characteristics: Work infrastructure | Jabot et al. 2020 |
| Organisational structure of a municipality (high degree of formality - fixed rules and procedures)/ structure and working patterns of public administration. | Inner setting | Structural characteristics | Kraemer, Nikolajsen, and Gulis 2014. Morteruel et al. 2020 |
| Lack of a culture of public participation in government affairs  Lack of established intersectoral work culture | Inner setting | Compatibility | Morteruel et al. 2020 |
| Perception that health, thus HIA, considered outside the remit of some sectors, lack of attention to health from other departments within the municipal | Inner setting | Mission alignment | Kraemer, Nikolajsen, and Gulis 2014 |
| Belief that health is already a consideration across sectors | Inner setting | Relative priority | Kraemer, Nikolajsen, and Gulis 2014 |
| Several other prevention and health promotion tasks already consuming time | Inner setting | Relative Priority | Kraemer, Nikolajsen, and Gulis 2014 |
| Governmental economic incentives to carry out certain types of prevention and health promotion projects | Inner setting | Organisational incentives and rewards | Kraemer, Nikolajsen, and Gulis 2014 |
| Legacy of EIA being a thorn in the side of industry | Individual | Knowledge & Beliefs  about the Innovation | Mattig et al. 2017 |
| Perception of recommendations being unduly critical | Individual | Knowledge & Beliefs  about the Innovation | Harris-Roxas et al. 2014 |
| Lack of agency: (HIA forced on them/no control) | Individual | Self-efficacy (including agency) | Harris-Roxas et al. 2014 |
| Lacking competency and legitimacy to carry out HIA mandate – feelings of inadequate training provided | Individual | Self-efficacy (including agency) | Jabot et al. 2020 |
| Decision making powers in the hands of politicians regardless of what evidence is put forward/politics and belief politicians unlikely to use HIA evidence | Individual | Self-efficacy | O’Mullane, 2014 |
| Lack of experience in carrying out HIA | Individual | Self-efficacy | Gamache et al. 2020, Ison 2013 |
| Dissatisfaction with implementation of HIA and its effects | Individual | Knowledge and beliefs | Liu et al. 2023 |
| Varying interpretation of HIA objectives and anticipated affects which may influence trust in process and lead to negative evaluations | Individual | Knowledge and beliefs | Liu et al. 2023 |
| Belief that direct community involvement (opposed to formal community representation) will hinder progressing the HIA | Individual | Knowledge & Beliefs about the Innovation | (Fakhri, Harris, and Maleki 2015, |
| Lack of substantial research results and useful recommendations | Individual | Knowledge & Beliefs about the Innovation | Gamache et al. 2020, |
| HIA Skepticism | Individual | Knowledge & Beliefs about the Innovation | Gamache et al. 2020, Ison 2013 |
| Feeling of superficial involvement amongst working groups as not all familiar with HIA | Individual | Knowledge & Beliefs about the Innovation | Gamache et al. 2020, |
| Low awareness of the concept of HIA/newness of concept/inconsistency in concept | Individual | Knowledge & Beliefs  about the Innovation | Damari, Vosoogh-Moghaddam, and Riazi-Isfahani 2018, Ison 2013, Marincová, Loosova and Valenta 2020 |
| Potentially too focussed on a particular minority group (limiting other potential health equity impacts on other disadvantaged groups) | Individual | Knowledge & Beliefs  about the Innovation | Harris-Roxas et al. 2014 |
| Idea that HIA is an admin burden rather than a force for public health promotion | Individual | Knowledge & Beliefs about the Innovation | O’Mullane, 2014 |
| Differing perceptions of the purpose of HIA (and legitimacy) | Individual | Knowledge & Beliefs about the Innovation characteristics | Harris-Roxas et al. 2014 |
| Consistency and meaningfully incorporating equity and vulnerable populations (from capturing diverse experiences to engaging key stakeholders) | Process | Engaging: innovation recipients | Bourcier et al. 2015, |
| Lack of skills and confidence of the local community to fully participate and interact with agency providers in working groups | Process | Assessing Needs/ innovation recipients | Purcell & Kearns 2013 |
| Scientific language not accessible to community within the working group | Process | Assessing Needs: innovation recipients | Purcell & Kearns 2013 |
| Following up on application of HIA recommendations/monitoring recommendations | Process | Reflecting and evaluating: implementation | Bourcier et al. 2015, Damari, Vosoogh-Moghaddam, and Riazi-Isfahani 2018 |
| Government buy in/supportive institutions | Process | Engaging | Damari, Vosoogh-Moghaddam, and Riazi-Isfahani 2018; Fakhri, Harris, and Maleki 2015; |
| Limited number of people involved in HIA and no involvement of people responsible for implementing recommendations | Process | Engaging | Harris-Roxas et al. 2014 |
| Perceived lack of dissemination of past research, gathered by previous bodies, to the community | Process | Engaging: trust | Purcell & Kearns 2013 |
| Partial adherence to practice standards (ie. public involvement) | Process | Adapting | Jabot et al. 2020 |
| Lack of an agreed implementation plan for the recommendations | Process | Adapting | Morteruel et al. 2020 |

## Table 2: Measures and strategies which may improve and strengthen HIA Implementation (Group one papers, N=27)

| **HIA Implementation Facilitators (Group One, n=27)** | | | |
| --- | --- | --- | --- |
| **Measure** | **Domain** | **Construct: subconstruct** | **Source** |
| HIA Led by an independent person with no political constraints. Ie. an academic without responsibility for health or urban planning | Individual | Role: implementation leads | Gamache et al. 2020 |
| Building awareness and understanding of how HIA can contribute to multiple stakeholders’ agendas and its added value | Innovation | Relative advantage | Jabot et al. 2020; O’Mullane 2014 |
| Experience in carrying out HIA and confidence in HIA methodology | Individual | Implementation Facilitators | Ison 2013; Jabot et al. 2020 |
| Involving stakeholders such as decision makers, people with knowledge about and access to decision making processes | Individual | High level leaders | Bourcier et al. 2015; Harris-Roxas et al. 2014; Haigh et al. 2015 |
| Involving people with knowledge, and access to decision making processes, and people with relevant skills as early as possible | Individual | Implementation facilitators/lead | Haigh et al. 2015 |
| Highlighting welfare costs of inaction - economic and social | Innovation | Relative advantage | Mattig et al. 2017; Thondoo et al. 2020 |
| Meaningfully engaging with and involving key stakeholders: community, decision makers, influential people, experts with specific knowledge | Process | Engaging | Bourcier et al. 2015; Gamache, Diallo, and Lebel 2022; Buregeya, Loignon, and Brousselle 2020; Haigh et al. 2015; Fischer, Chang, Muthoora et al. 2024 |
| Involving local community stakeholders and building networks with HIA commissioning organisations | Process | Engaging | Fischer, Chang, Muthoora et al. 2024 |
| Paying attention to national policymaking and planning systems and considering broad contextual factors such as political contexts: recognising policy windows – opportunities. Focussing HIA on broad societal concerns to encourage social acceptance and linking actions to outside of the HIA process | Process | Assessing context | Damari, Vosoogh-Moghaddam, and Riazi-Isfahani 2018; Fakhri and Harris 2021; Haigh et al. 2015; Kraemer, Nikolajsen, and Gulis 2014; Gamache et al. 2020 |
| Flexibility in process to adapt to circumstances. Adapting HIA to organisation and legislative context and political and-administrative context: adapting language to municipal realties, | Process | Adapting | Haigh et al. 2015; Gamache et al. 2020; Jabot et al. 2020 |
| Encouraging of understanding and the recognition of common goals and shared interests among key agents | process | Engaging | Morteruel et al. 2020; Gamache et al. 2020 |
| Involving private project promoters to show that HIA can improve projects at a low cost and encourage social acceptance | Individuals | Implementation facilitators | Gamache et al. 2020 |
| Highlighting social responsibility of decision makers | Process | Engaging | Thondoo et al. 2020 |
| Highlight return on investment – cost effectiveness/monetary value | Innovation | Relative advantage | Mattig et al. 2017; Thondoo et al. 2020 |
| Collaboration with an academic/public health institution or local health agency | Outer setting | Partnerships & Connections | Ison 2013 |
| Establishing a partnership between public health actors and municipalities | Outer setting AND Inner setting | Partnerships & Connections AND relational connections | Jabot et al. 2020; Linzalone et al. 2018 |
| Building good relationship and understanding between sectors and stakeholders | Outer setting | Partnerships & Connections | Morteruel et al. 2020; Ison 2013; Liu et al. 2023 |
| Establishing good communication channels – reporting and presenting skills of those involved in HIA | Individual | Implementation facilitators | Kraemer, Nikolajsen, and Gulis 2014 |
| Collaboration with media to introduce and explain HIA and facilitate buy-in and awareness of potential benefits and value of HIA | Outer setting | Partnerships & Connections | Marincová, Loosova, and Valenta 2020 |
| Confronting misunderstandings and creating awareness of scope and effectiveness of HIA | Innovation | Relative advantage | Mattig et al. 2017 |
| Hosting/presenting at events to communicate HIA information | Individual |  | Kraemer, Nikolajsen, and Gulis 2014 |
| Promoting a broader understanding of health: social model of health | Outer setting | Local conditions: education systems | O’Mullane 2014; Linzalone et al. 2018 |
| Improving technical and governance capacities to enhance the awareness of the potentiality of HIAs | Inner setting | Structural characteristics: governance and communications | Linzalone et al. 2018 |
| Involvement of interdisciplinary students (i.e.. public health and urban planners) in HIA in preparation for next generation of urban planners | Outer setting | Local conditions: education systems | Gamache et al. 2020 |
| Creating education system for experts, employees of the Public Health Authority and public health students, and support capacity building. | Outer setting | Local conditions: education systems | Marincová Loosova, and Valenta 2020 |
| Including public health related teachings such as social medicine and health policy making in the Ministry of Health and Medical Education and promoting interdisciplinary research | Outer setting | Local conditions: education systems | Damari, Vosoogh-Moghaddam, and Riazi-Isfahani 2018; Liu et al. 2023 |
| Supporting and promoting Intersectoral and multisectoral work practices and collaboration | Outer setting AND Inner setting | Partnerships & Connections AND relational connections | Gamache et al. 2020; Haigh et al. 2015; Thondoo, et al. 2020a; Walpita and Green 2022; Kraemer, Nikolajsen, and Gulis 2014; Marincová, Loosova, and Valenta 2020; Liu et al. 2023 |
| Training in HIA, including multi-institutional training and training on determinants of health | Inner setting  AND  Outer setting | Available Resources: Access to Knowledge & information | Ison 2013; Linzalone et al. 2018; O’Mullane 2014; Walpita and Green 2022 |
| Access to information and HIA expertise, i.e.. national access point to health portal containing a registry of case studies and health data (Linzalone et al 2018) | Inner setting AND  Outer setting | Available Resources: Access to Knowledge & information | Ison 2013, O’Mullane 2014, Linzalone et al 2018 |
| Providing HIA education and training | Inner setting AND Outer setting | Access to knowledge and information and Local conditions | Liu et al. 2023 |
| Improving perceived value of HIA and trust in the process | Inner setting AND Outer setting | Access to knowledge and information and Local conditions | Liu et al. 2023 |
| Development of guidelines and tools, including adaptable and easy to use guidance | Inner setting  AND  Outer setting | Access to knowledge and information | Quin, Carmichael, and Hopper 2023; Thondoo et al. 2020; Marincová, Loosova, and Valenta 2020; Kraemer, Nikolajsen, and Gulis 2014 |
| Access to HIA resources in several languages | Inner setting AND Outer setting | Available Resources: Access to Knowledge & information | Marincová, Loosova, and Valenta 2020; Kraemer, Nikolajsen, and Gulis 2014; O Mullane 2014 |
| Establishing HIA units within and across countries | Outer setting | Partnerships & Connections | Marincová, Loosova, and Valenta 2020; O’Mullane 2014; Walpita and Green 2022 |
| Encouraging membership with external HIA networks, Health city networks and creating HIA platforms | Outer setting | Partnerships & Connections | Kraemer, Nikolajsen, and Gulis 2014; Mattig et al. 2017; O’Mullane 2014 |
| Financial support/allocation of funding for HIA | Inner setting AND Outer setting | Available resources: Funding and financing | Linzalone et al. 2018; Fakhri, Harris, and Maleki 2015; Morteruel et al. 2020; Gamache et al. 2020; Thondoo et al. 2020a |
| Having dedicated HIA staff/posts within organisations including HIA ‘champions’ | Inner setting | Available resources | Ison 2013; Fischer, Chang, Muthorra 2024 |
| Guidance on how to access necessary data for private proponents | Inner setting  AND Outer setting | Available Resources: Access to Knowledge & information | Linzalone et al. 2018 |
| Peer learning to advance and support HIA assessment methods, objective analysis, evaluation and discussion by multidisciplinary experts | Inner setting  AND Outer setting | Available Resources: Access to Knowledge & information | Liu et al. 2023 |
| Creating Supplementary Planning Documents (SPDs) to help developers understand what they need to do and what the local authority would expect | Inner setting AND Outer setting | Available Resources: Access to Knowledge & information | Quin, Carmichael, and Hopper 2023 |
| Training for both community and local service agency members engaging in HIA | Inner setting | Available Resources: Access to Knowledge & information | Pursell and Kearns 2013 |
| Building community capacity to engage in HIA process | Process | Engaging: recipients | Haigh et al. 2015; Pursell and Kearns 2013 |
| Investing time at the beginning of the HIA process to discuss and clarifying purposes, goals, values and expected outcomes | Process | Planning (to allow for time in HIA process to achieve this) | Haigh et al. 2015 |
| Diverse, interdisciplinary, multidisciplinary HIA teams with a range of competencies, skills and expert knowledge | Individual | Implementation leads/facilitators | Bourcier et al. 2015; Busato and Grisotti 2022; Haigh et al. 2015; Jabot et al. 2020; Morteruel et al. 2020 |
| Establishing shared values, culture, explicit goals and clearly defined roles and responsibilities. | Process | Planning | Haigh et al. 2015, Jabot et al. 2020 |
| Identifying relevant stakeholders and points of influence within systems and consider how the HIA can affect these | Process | Assessing context | Haigh et al. 2015 |
| Meaningfully engaging and involving key stakeholders: community, decision makers, influential people | Process | Engaging | Bourcier et al 2015, Gamache et al. 2022, Buregeya et al 2020 |
| Providing space for citizen participation | Process | Engaging: recipients | (Morteruel et al. 2020; Thondoo et al. 2020) |
| Encouraging participation of affected populations at all stages | Process | Engaging recipients | Gamache, Diallo, and Lebel 2022 |
| Recognizing and addressing the power discrepancies and cultural and language barriers between service agencies and communities | Process | Assessing needs | Pursell and Kearns 2013 |
| Setting realistic expectations from the onset | Process | Assessing context | Kraemer, Nikolajsen, and Gulis 2014 |
| Developing methodological solutions to conduct stakeholder consultation (scoping stage to select issues that matter) | Process | Assessing needs | Linzalone et al. 2018 |
| More in-depth consideration and inclusion of different vulnerable groups and of citizens opinions | Process | Assessing needs | Busato and Grisotti 2022 |
| Flexibility in process to adapt to circumstances | process | Adapting | Haigh et al. 2015; Gamache et al. 2020; Jabot et al. 2020 |
| Incorporating indirect health impacts when assessing impacts | Process | Doing | Fakhri, Harris, and Maleki 2015; Westenhöfer et al 2023 |
| Using locally relevant data and considering the scientific evidence and contextualising scientific evidence with local evidence | Process | Doing | Bourcier et al. 2015; Busato and Grisotti 2022; Morteruel et al. 2020 |
| Paying more attention to the needs of vulnerable populations | Process | Assessing needs: recipients | Bourcier et al. 2015 |
| In view of limited resources, the use of simple tools to bring a health perspective to decisions and the use of pre-existing structures and procedures were cited as potential good practices that could be used as an alternative to a full HIA | Process | Assessing context | Morteruel et al. 2020 |
| Reporting results concisely and providing a shorter version of the report for stakeholders | Process | Doing | Gamache, Diallo, and Lebel 2022 |
| Willingness to consider lay knowledge alongside expert opinion | Process | Doing | Thondoo et al. 2020; |
| Providing greater transparency re. decision making, accountability and recommendations. | Process | Doing | Busato and Grisotti 2022; Morteruel et al. 2020; Gamache, Diallo, and Lebel 2022; Linzalone et al. 2017; Liu et al. 2023) |
| Establishing cross-sectoral collaborative mechanisms to allow review of implementation issues that arise and enhance acknowledgement and trust among participants | Process | Doing | Liu et al. 2023 |
| Creating recommendations that are actionable, realistic, and sector-specific that consider the decisionmakers authority to act, timelines and potential costs | Process | Doing | Bourcier et al. 2015 |
| Increased scrutiny of monitoring stages of HIA to bridge evidence gap though case studies of HIA illustrating health outcomes | Process | Reflecting and evaluating | Fischer, Chang and Muthoora 2024 |
| Designing equality themed workshop with impacted populations and paying people for their time. | Process | Doing | Fischer, Chang and Muthoora 2024 |
| Considering if momentum is growing for a particular issue, if decisionmakers have basic knowledge about health relate issues, what connections exist between those conducting the HIA and decisionmakers, and how the timing of the HIA fits with the decision-making process | Process | Assessing context | Bourcier et al. 2015 |
| Reinforcing and strengthening assessment quality and rigour to build trust amongst stakeholders and improve HIA practice | Process | Doing | Marincová, Loosova and Valenta 2020; Liu et al.2023). |
| Ensuring that the recommendations reflect what the community impacted want | Process | Doing | Fischer, Chang and Muthoora 2024 |
| Compulsory follow up of recommendations to strengthen perceived value of HIA | Process | Reflecting and evaluating | Fischer, Chang and Muthoora 2024 |

## Table 3: Potential HIA Implementation facilitators (Group 2 papers, N=8)

| **Potential facilitators identified within Group 2 papers** | **Focus of action** | **Domain** | **Construct: subcontract** | **Source** |
| --- | --- | --- | --- | --- |
| Addressing confusion between HIA and other procedures. | Providing clarity in HIA purpose and process/differentiation | Innovation | Relative advantage | Jabot & Rivadeneyra-Sicilia 2022 |
| Adequate adaption of international HIA standards to political-administrative context | Adapting HIA standards to local context | Innovation | Adaptability | Jabot & Rivadeneyra-Sicilia 2022 |
| Recognizing and promoting HIA as a tool that can be used to promote HiAP | Promoting/advocating HIA as a tool to support HIaP | Innovation | Relative advantage | Goff et al. 2016, Jabot & Rivadeneyra-Sicilia 2022 |
| Actors situated at the interface of different professional worlds play a role in disseminating innovations | Working across sectors and professions to promote HIA | Outer setting | Partnerships and connections | Roue le-Gall & Jabot 2017 |
| Partnership between community-based organisations and health practitioners | Collaboration and partnerships between health professionals and community organisations | Outer setting | Partnerships & Connections | Bever et al. 2021 |
| Long-standing and close collaboration between the regional and the local authorities on social and health issues. | Fostering collaboration and partnerships between local and regional authorities | Outer setting | Partnerships & Connections | Jabot & Rivadeneyra-Sicilia 2022; Roue le-Gall & Jabot 2017 |
| Ongoing collaboration (municipal and metropolitan officers and academia) to enable access to local data and funding to produce HIAs in the metropolitan area.  Creating partnerships with other public institutions to provide new sources of funding | Fostering collaboration for access to data and funding | Outer setting | Partnerships & Connections | Ramirez-Rubio 2019; Jabot & Rivadeneyra-Sicilia 2022 |
| Being a member of the Healthy Cities Network | Collaboration: Being a member of Healthy Cities  But also due to national momentum | Outer setting | Partnerships & Connections | Roue le-Gall & Jabot 2017 |
| Creating an HIA network | Creating a new, or collaborating with an existing HIA network | Outer setting | Partnerships & connection | Goff et al. 2016 |
| Inclusion of HIA in local and regional plans (ie. in the health and environment programme, sustainable cities, and local health contracts) | Promoting HIA in local and regional plans  ‘ie Inserting HIA into a local health contract is an opportunity to explain HIAs; to promote their use; and seek financing from numerous partners’ | Outer setting | Policies and laws | Roue le-Gall & Jabot 2017 |
| Embedding HIA within planning tools was an opportunity for connecting health and urban planning | Embedding HIA in planning tools | Outer setting | Policies and laws | Roue le-Gall & Jabot 2017 |
| HIA dedicated sessions are being included in national conferences to disseminate knowledge and to encourage the sharing of experiences | Building capacity | Outer setting | Local conditions: educational settings | Jabot & Rivadeneyra-Sicilia 2022 |
| A supportive political and legal context | Supportive political and legislative context | Outer setting | Policies and Laws | Ramirez-Rubio 2019 |
| HIA development in France stems from of active advocacy by health promotion actors at the Regional Health Agencies | Advocacy | Outer setting | External pressure: Societal pressure | Jabot & Rivadeneyra-Sicilia 2022 |
| Encouraging greater political commitment from the national health authorities help to encourage HIA development | Political will | Outer setting | Local conditions | Jabot & Rivadeneyra-Sicilia 2022 |
| Leveraging existing programs and networks | Networks | Outer setting | Partnerships and connections | Goff et al. 2016 |
| Building HIA programs using staff capacity in related activities  Ie community engagement, policy analysis, health and education are examples of routine work activities already employed in SHA | Capacity: Leveraging existing capacity | Inner setting | Available resources | Goff et al. 2016 |
| Considering the sustainability of HIA programs in the absence of grant funding. Embedding HIA skills. | Training staff in complementary programs to enable them to utilize HIA after funding ends | Inner setting | Available resources | Goff et al. 2016 |
| France - training activities are in place | Capacity | Inner setting | Access to knowledge and information | Jabot & Rivadeneyra-Sicilia 2022 |
| Training session that provides an overview of HIA principles and processes and showcasing a relevant case study | Capacity | Inner setting | Access to knowledge and information | Goff et al. 2016 |
| Working in partnership with local stakeholders and utilizing local knowledge and experience; | Involving local stakeholders | Process | Engaging | Goff et al. 2016 |
| Active civic participation to assist in identifying what will be analysed and to understand links between project and impacts on quality of life and equity | Involving citizens | Process | Engaging | Roue le-Gall & Jabot 2017 |
| Utilizing subject matter experts from other disciplines | Process: Multidisciplinary expertise | Process | Engaging: innovation deliverers | Goff et al. 2016 |
| Identifying goals and standards at the start of the process; | Process: HIA goals and standards at the beginning of the process | Process | Doing | Goff et al. 2016 |
| Periodic reassessment of stakeholder interests and continued public information-sharing by city and county decision-makers | Changing priorities (changes in elected leaders, funding uncertainty, shift in community priorities) | Process | Reflecting and evaluating | Bever et al. 2021 |
| Early introduction of HIA to enable draft projects and specifications to evolve to ensure that recommendations were implemented in the development project | Process: Early introduction of HIA in draft stages | Process | Planning | Roue le-Gall & Jabot 2017 |
| A willingness to go further than just straightforward consultation by taking a ‘co-construction’ approach with citizens potentially impacted by the proposal. | Meaningful engagement | Process: | engaging | Roue le-Gall & Jabot 2017 |
| Clear identification of roles: actors responsible for activities within different phases (project planning, design guidance, conducting HIA, implementing recommendations) must be clearly identified to avoid confusion between these different activities | Clear identification of roles | Process | doing | Ramirez-Rubio et al. 2019 |
| Engaging internal leadership, management, and staff simultaneously  IE. Leaders and management who recognise the value of HIA and dedicate resources | Engagement | Process | Engagement | Goff et al. 2016 |
| Starting with the connections between health and the built environment | Direct health impacts | Process | Doing | Goff et al. 2016 |
| Starting with a rapid HIA to begin capacity building | Capacity: Rapid HIA to build understanding and capacity | Process | Doing | Goff et al. 2016 |
| Contextualising data (local with data from other setting) | Data modelling | Process | doing | Ramirez-Rubio et al. 2019 |
| Systematic use of solid methods | Solid methods | Process | Doing | JaboT & Rivadeneyra-Sicilia 2022 |
| Tailoring training to the stakeholder role in the HIA process (people outside Public Health Sector) | Capacity: Training | Process | Tailoring strategies | Goff et al. 2016 |

## Table 4: Factors impeding HIA implementation (Group 2 papers, N=8)

| **Barriers and considerations** | **Main challenge** | **CFRI Domain** | **Construct** | **Source** |
| --- | --- | --- | --- | --- |
| HIAs may not be appropriate or feasible (specific to housing practitioners) given time and cost considerations | Cost and time constraints | Innovation | Cost | Bever et al. 2021 |
| The lack of high-quality data in LMIC/poor-resources settings | Scarcity of data | Innovation | Evidence base | Ramirez-Rubio et al. 2019 |
| Timescale for an urban development project is restrictive for the normally much shorter-lived HIA | Timescale | Innovation | Adaptability | Roue le-Gall & Jabot 2017 |
| Lack of quality in baseline data of quantitative HIAs as a major limitation | Scarcity of baseline data | Innovation | Evidence base | Thondoo et al. 2019 |
| Need for further study on data availability, adapted models/tools, low technical capacity, | Data, technical capacity, models and tools, | Innovation | Evidence base | Thondoo et al. 2022 |
| Sustained and systematic use of HIA is dependent on the availability of resources. | Resources | Inner setting | Available resources | Jabot & Riveneyra 2022 |
| Low policy demand for evidence for modelling studies (re. scaling and expanding quant. health impact modelling in LMIC) | Political demand | Outer setting | Local conditions | Thondoo et al. 2022 |
| HIAs’ influence may only be short-term in contexts where property ownership or policies change rapidly | Influence | Outer setting | Local conditions | Bever et al. 2021 |
| HIAs reliance on policy makers being able and willing to invest in, or collaborate with, institutions to complement existing datasets with newly conducted surveys and studies | Commitment and collaboration | Outer setting | Partnerships and connections | Ramirez-Rubio et al. 2019 |
| Policy makers making time to engage in HIA. | Engagement | Process | Engagement | Ramirez-Rubio et al. 2019 |
| Reporting stakeholder engagement transparently | Transparency in reporting | Process | Doing | Ramirez-Rubio et al. 2019 |

## Table 5. HIA case studies factors impeding implementation (Group 3, N=10)

| **Barriers and considerations** | **Main challenge** | **CFRI Domain** | **Construct** | **Source** |
| --- | --- | --- | --- | --- |
| Review and reflection perceived as more actionable than ‘monitoring and evaluation’ | Complexity | Innovation | complexity | Green et al. 2020 |
| Lack of capacity and resource allocation | Resources | Inner setting | Access to resources | Kogel et al. 2020 |
| Lack of systematic HIA processes and evidence to facilitate implementation | Innovation | Innovation | Evidence base | Kogel et al. 2020 |
| Lack of diversity in HIA team as a barrier for reaching particular populations (language, culture) | HIA team | Individual | Roles: Implementation facilitators | Negev et al.2013 |
| Group facilitators not controlling for bias (i.e.. some participants being more ‘powerful’ than others’, | HIA Team | Individual | Roles: implementation facilitators | Negev et al. 2013 |
| Experts not valuing local knowledge or local risk perceptions | Ontological | Individual | Knowledge & Beliefs  about the Innovation | Negev et al. 2013 |
| Time and resources required to ensure greater representation in the HIA process. | Available resources | Inner setting | Available resources | Negev et al. 2020 |
| Lack of formal commitment from the local administration at the preliminary stages delayed early engagement and setting up of network involving local communities | Lack of commitment from public bodies | Outer setting | Local conditions | Linzalone et al.2017 |
| Multiplicity of stakeholders (knowledge, conflicting interests, priorities) in policy decision making | Transparency | Process | Doing | Linzalone et al. 2017 |
| *Hidden stakeholder agendas | Transparency | Process | Doing | Del Rio et al. 2017 |

## Table 6. Factors facilitating implementation identified in group 3 studies

| **Facilitating factors** | **Focus of Action** | **Domain** | **Construct** | **Source** |
| --- | --- | --- | --- | --- |
| Public health actors prioritising support for HIA and investing in capacity and resource allocation | Prioritising and investing/ advocacy | Outer setting domain | Local conditions | Kogel et al. 2020 |
| Review and reflection meetings with HIA leads (experts) and teams (researchers and public health practitioners) at the end of the process | Reflection of the process after completion | Process | Reflecting and evaluating | Green et al.2020 |
| Multidisciplinary, cross-sector steering/advisory group and working group | Ensuring diversity in skills and knowledge within the HIA teams | Individual | Implementation Team members | Green et al. 2020 |
| Adapting HIA to local circumstances and needs (diversity in stakeholders, resources required) | Adapting the HIA to the local context | Process | Adapting | Negev et al.2013 |
| Non-controversial nature of WSD project, thus a safe platform for building capacity and highlighting utility of HIA in India | Assessing the political context of HIA | Process | Assessing context | Pradyumna et al. 2021 |
| multicultural stakeholder participation can uncover unaccounted for health issues, is a means to access local knowledge and as a means to scope health issues for the HIA that reflect the priorities of the people being impacted by the plan | Ensuring diverse public participation in the HIA | process | Engaging | Negev et al. 2013 |
| Mapping stakeholders to include diverse representation | Preparing and planning for diverse representation | Process | doing | Negev et al.2013 |
| Designing committee meetings to maximize diverse interactions* | Preparing and planning meeting | Process | Planning | Negev et al. 2013 |
| HIA training for those leading HIAs | Ensuring access to training for those involved in HIA | Inner setting | Access to knowledge and information | Sheffield et al. 2014 |
| A steering group/advisory group made up of professionals along with a community advisory committee made up of community representatives | Having a community advisory committee in addition to HIA steering/advisory group | Individual | Implementation team members | Sheffield et al.2014 |
| Review of published and grey literature at screening stage of HIA for insight into potential health impacts | Reviewing potential health impacts at screening stage | Process | Doing | Sheffield et al. 2014 |
| Public forum at preliminary phase of HIA to build trust among stakeholders (political-admin and civil society) and to recruit for HIA | Building trust and awareness among stakeholders before the HIA begins | Process | Engaging | Linzalone et al.2017 |
| Involvement of local government institutions to provide access to basic data and promote inter-departmental collaboration | Collaborating with institutions to access relevant data | Outer setting | Partnership and connection | Linzalone et al. 2017 |
| Stakeholder (including civil society) contribution at all stages in the decision making | Civil society involvement at all stages of HIA | Individual | Implementation team members | Linzalone et al. 2017 |
| Early and wider engagement in the process helped public institutions and community groups gain a better understanding of each other’s positions | Promoting shared understanding of the HIA through engagement | Process | Engaging | Linzalone et al.2017 |

**References**

Berensson, K., & Tillgren, P. (2017). Health impact assessment (HIA) of political proposals at the local level: Successful introduction, but what has happened 15 years later? *Global Health Promotion*, *24*(2), 43–51. https://doi.org/10.1177/1757975916683386

Bever, E., Arnold, K. T., Lindberg, R., Dannenberg, A. L., Morley, R., Breysse, J., & Pollack Porter, K. M. (2021). Use of health impact assessments in the housing sector to promote health in the United States, 2002–2016. *Journal of Housing and the Built Environment*, *36*(3), 1277–1297. https://doi.org/10.1007/s10901-020-09795-9

Bourcier, E., Charbonneau, D., Cahill, C., & Dannenberg, A. L. (2015). An Evaluation of Health Impact Assessments in the United States, 2011–2014. *Preventing Chronic Disease*, *12*, 140376. https://doi.org/10.5888/pcd12.140376

Buregeya, J. M., Loignon, C., & Brousselle, A. (2020). Contribution analysis to analyze the effects of the health impact assessment at the local level: A case of urban revitalization. *Evaluation and Program Planning*, *79*, 101746–15. https://doi.org/10.1016/j.evalprogplan.2019.101746

Busato, M. A., & Grisotti, M. (2022). Health impact assessment in the process of implementation of hydroelectric plants: Methodological contributions. *Ambiente & Sociedade*, *25*(Journal Article). https://doi.org/10.1590/1809-4422asoc20200068r1vu2022l3oa

Damari, B., Vosoogh-Moghaddam, A., & Riazi-Isfahani, S. (2018). Implementing health impact assessment at national level: An experience in Iran. *Iranian Journal of Public Health*, *47*(2), 246–255. https://go.exlibris.link/XVYpPW91

Del Rio, M., Hargrove, W. L., Tomaka, J., & Korc, M. (2017). Transportation Matters: A Health Impact Assessment in Rural New Mexico. *International Journal of Environmental Research and Public Health*, *14*(6). https://doi.org/10.3390/ijerph14060629

Fakhri, A., & Harris, P. (2021). Internationally validating a conceptual framework for health impact assessment. *International Archives of Health Sciences*, *8*(4), 231–236. https://doi.org/10.4103/iahs.iahs_42_21

Fakhri, A., Harris, P., & Maleki, M. (2015). Proposing a framework for Health Impact Assessment in Iran. *BMC Public Health*, *15*(1), 1–7. https://doi.org/10.1186/s12889-015-1698-1

Fischer, T. B., Chang, M., & Muthoora, T. (2024). Health impact assessment in two planning projects in England: Reflections on normative effectiveness. *BMC Public Health*, *24*(1), 2819. https://doi.org/10.1186/s12889-024-20203-7

Gamache, S., Diallo, T., & Lebel, A. (2022). The use of health impact assessments performed in Quebec City (Canada) – 2013–2019: Stakeholders and participants’ appreciation. *Environmental Impact Assessment Review*, *92*(Journal Article), 106693. https://doi.org/10.1016/j.eiar.2021.106693

Gamache, S., Lebel, A., Diallo, T. A., & Shankardass, K. (2020). The elaboration of an intersectoral partnership to perform health impact assessment in urban planning: The experience of quebec city (canada). *International Journal of Environmental Research and Public Health*, *17*(20), 1–15. https://doi.org/10.3390/ijerph17207556

Goff, N., Wyss, K., Wendel, A., & Jarris, P. (2016). Implementing Health Impact Assessment Programs in State Health Agencies: Lessons Learned From Pilot Programs, 2009-2011. *Journal of Public Health Management and Practice : JPHMP*, *22*(6), E8–E13. https://doi.org/10.1097/PHH.0000000000000392

Green, L., Ashton, K., Edmonds, N., & Azam, S. (2020). Process, Practice and Progress: A Case Study of the Health Impact Assessment (HIA) of Brexit in Wales. *International Journal of Environmental Research and Public Health*, *17*(18), 1–14. https://doi.org/10.3390/ijerph17186652

Green, L., Gray, B. J., & Ashton, K. (2020). Using health impact assessments to implement the sustainable development goals in practice: A case study in Wales. *Impact Assessment and Project Appraisal*, *38*(3), 214–224. https://doi.org/10.1080/14615517.2019.1678968

Haigh, F., Baum, F., Dannenberg, A. L., Harris, M. F., Harris-Roxas, B., Keleher, H., Kemp, L., Morgan, R., Chok, H. N., Spickett, J., & Harris, E. (2013). The effectiveness of health impact assessment in influencing decision-making in Australia and New Zealand 2005-2009. *BMC Public Health*, *13*(1), 1188–1188. https://doi.org/10.1186/1471-2458-13-1188

Haigh, F., Harris, E., Harris-Roxas, B., Baum, F., Dannenberg, A. L., Harris, M. F., Keleher, H., Kemp, L., Morgan, R., Chok, H. N. G., & Spickett, J. (2015). What makes health impact assessments successful? Factors contributing to effectiveness in Australia and New Zealand. *BMC Public Health*, *15*(1), 1009–1009. https://doi.org/10.1186/s12889-015-2319-8

Harris-Roxas, B., Haigh, F., Travaglia, J., & Kemp, L. (2014). Evaluating the impact of equity focused health impact assessment on health service planning: Three case studies. *BMC Health Services Research*, *14*(1), 371–371. https://doi.org/10.1186/1472-6963-14-371

Ison, E. (2013). Health Impact Assessment in a Network of European Cities. *Journal of Urban Health*, *90*(Suppl 1), 105–115. https://doi.org/10.1007/s11524-011-9644-8

Jabot, F., & Rivadeneyra-Sicilia, A. (2022). Health impact assessment institutionalisation in France: State of the art, challenges and perspectives. *IMPACT ASSESSMENT AND PROJECT APPRAISAL*, *40*(3), 179–190. https://doi.org/10.1080/14615517.2021.2012011

Jabot, F., Tremblay, E., Rivadeneyra, A., Diallo, T. A., & Lapointe, G. (2020). A comparative analysis of health impact assessment implementation models in the regions of montérégie (Québec, canada) and nouvelle-aquitaine (france). *International Journal of Environmental Research and Public Health*, *17*(18), 1–18. Scopus. https://doi.org/10.3390/ijerph17186558

Kögel, C. C., Peña, T. R., Sánchez, I., Tobella, M., López, J. A., Espot, F. G., Claramunt, F. P., Rabal, G., & Viana, A. G. (2020). Health impact assessment (HIA) of a fluvial environment recovery project in a medium-sized Spanish Town. *International Journal of Environmental Research and Public Health*, *17*(5), 1484. https://doi.org/10.3390/ijerph17051484

Kraemer, S. R. J., & Gulis, G. (2014). How do experts define relevance criteria when initiating Health Impact Assessments of national policies? *Scandinavian Journal of Public Health*, *42*(1), 18–24. https://doi.org/10.1177/1403494813504254

Kræmer, S. Johnsdatter, R., Theilgaard Nikolajsen, L and Gulis, G. (2014). Implementation of health impact assessment in Danish municipal context. *Central European Journal of Public Health*, *22*(4), Article 4. https://doi.org/10.21101/cejph.a3943

Linzalone, N., Ballarini, A., Piccinelli, C., Viliani, F., & Bianchi, F. (2018). Institutionalizing Health Impact Assessment: A consultation with experts on the barriers and facilitators to implementing HIA in Italy. *Journal of Environmental Management*, *218*, 95–102. https://doi.org/10.1016/j.jenvman.2018.04.037

Linzalone, N., Coi, A., Lauriola, P., Luise, D., Pedone, A., Romizi, R., Sallese, D., Bianchi, F., Santoro, M., Minichilli, F., Maurello, M. T., Scaringi, M., Zuppiroli, M. E., HIA21 Project Working Group, & HIA21 Project Working Grp. (2017). Participatory health impact assessment used to support decision-making in waste management planning: A replicable experience from Italy. *Waste Management (Elmsford)*, *59*(Journal Article), 557–566. https://doi.org/10.1016/j.wasman.2016.09.035

Liu, X., Liu, Y., Xu, Y., Song, L., Huang, Z., Zhu, X., & Zhang, M. (2023). Current status and influencing factors of policy identification in health impact assessment: A case study of Zhejiang Province. *Health Research Policy and Systems*, *21*(1), 118. https://doi.org/10.1186/s12961-023-01064-9

Marincova, L., Loosova, J., & Valenta, V. (2020). Experiences and needs of Licences Health Risk Assessors using Health Impact Assessment in the Czech Republic. *Central European Journal of Public Health*, *28*(2), 108–113. https://doi.org/10.21101/cejph.a5833

Mattig, T., Cantoreggi, N., Simos, J., Kruit, C. F., & Christie, D. P. T. H. (2017). HIA in Switzerland: Strategies for achieving Health in All Policies. *Health Promotion International*, *32*(1), 149–156. https://doi.org/10.1093/heapro/dav087

Morteruel, M., Bacigalupe, A., Aldasoro, E., Larrañaga, I., & Serrano, E. (2020). Health impact assessments in Spain: Have they been effective? *International Journal of Environmental Research and Public Health*, *17*(8), Article 8. https://doi.org/10.3390/ijerph17082959

Movia, M., Macher, S., Antony, G., Zeuschner, V., Wamprechtsamer, G., Delle Grazie, J., Simi, H., & Fuchs-Neuhold, B. (2022). Health Impact Assessment (HIA) of a Daily Physical Activity Unit in Schools: Focus on Children and Adolescents in Austria Up to the 8th Grade. *International Journal of Environmental Research and Public Health*, *19*(11), Article 11. https://doi.org/10.3390/ijerph19116428

Negev, M., Davidovitch, N., Garb, Y., & Tal, A. (2013). Stakeholder participation in health impact assessment: A multicultural approach. *Environmental Impact Assessment Review*, *43*(Journal Article), 112–120. https://doi.org/10.1016/j.eiar.2013.06.002

O’Mullane, M. (2014). Implementing the legal provisions for HIA in Slovakia: An exploration of practitioner perspectives. *Health Policy*, *117*(1), 112–119. Scopus. https://doi.org/10.1016/j.healthpol.2014.03.005

Pradyumna, A., Farnham, A., Utzinger, J., & Winkler, M. S. (2021). Health impact assessment of a watershed development project in southern India: A case study. *IMPACT ASSESSMENT AND PROJECT APPRAISAL*, *39*(2), 118–126. https://doi.org/10.1080/14615517.2020.1863119

Pursell, L., & Kearns, N. (2013). Impacts of an HIA on inter-agency and inter-sectoral partnerships and community participation: Lessons from a local level HIA in the Republic of Ireland. *Health Promotion International*, *28*(4), 522–532. https://doi.org/10.1093/heapro/das032

Quin, M., Carmichael, L., & Hopper, C. (2023). Implementing Health Impact Assessment policy on infrastructure development in the London Borough of Tower Hamlets. *Cities and Health*, *7*(3), 303–311. Scopus. https://doi.org/10.1080/23748834.2022.2148843

Ramirez-Rubio, O., Daher, C., Fanjul, G., Gascon, M., Mueller, N., Pajin, L., Plasencia, A., Rojas-Rueda, D., Thondoo, M., & Nieuwenhuijsen, M. J. (2019). Urban health: An example of a ‘health in all policies’ approach in the context of SDGs implementation. *Globalization and Health*, *15*(1), 87–87. https://doi.org/10.1186/s12992-019-0529-z

Roué-Le Gall, A., & Jabot, F. (2017). Health impact assessment on urban development projects in France: Finding pathways to fit practice to context. *Global Health Promotion*, *24*(2), 25–34. https://doi.org/10.1177/1757975916675577

Sheffield, P., Rowe, M., Agu, D., Rodríguez, L., & Avilés, K. (2014). Health Impact Assessments for Environmental Restoration: The Case of Caño Martín Peña. *Annals of Global Health*, *80*(4), 296–302. https://doi.org/10.1016/j.aogh.2014.07.001

Thondoo, M., De Vries, D. H., Rojas-Rueda, D., Ramkalam, Y. D., Verlinghieri, E., Gupta, J., & Nieuwenhuijsen, M. J. (2020). Framework for Participatory Quantitative Health Impact Assessment in Low- and Middle-Income Countries. *International Journal of Environmental Research and Public Health*, *17*(20), 1–20. https://doi.org/10.3390/ijerph17207688

Thondoo, M., Goel, R., Tatah, L., Naraynen, N., Woodcock, J., & Nieuwenhuijsen, M. (2022). The Built Environment and Health in Low- and Middle-Income Countries: A Review on Quantitative Health Impact Assessments. *Current Environmental Health Reports*, *9*(1), 90–103. https://doi.org/10.1007/s40572-021-00324-6

Thondoo, M., Mueller, N., Rojas-Rueda, D., de Vries, D., Gupta, J., & Nieuwenhuijsen, M. J. (2020a). Participatory quantitative health impact assessment of urban transport planning: A case study from Eastern Africa. *Environment International*, *144*, 106027. https://doi.org/10.1016/j.envint.2020.106027

Thondoo, M., Rojas-Rueda, D., Gupta, J., de Vries, D. H., & Nieuwenhuijsen, M. J. (2019). Systematic Literature Review of Health Impact Assessments in Low and Middle-Income Countries. *International Journal of Environmental Research and Public Health*, *16*(11), 2018. https://doi.org/10.3390/ijerph16112018

Walpita, Y. N., & Green, L. (2022). Health Impact Assessment (HIA): A Comparative Case Study of Sri Lanka and Wales: What Can a Developing Country Learn From the Welsh HIA System? *International Journal of Health Services*, *52*(2), 283–291. https://doi.org/10.1177/0020731420941454

Westenhöfer, J., Nouri, E., Reschke, M. L., Seebach, F., & Buchcik, J. (2023). Walkability and urban built environments-a systematic review of health impact assessments (HIA). *BMC Public Health*, *23*(1), 518–518. https://doi.org/10.1186/s12889-023-15394-4
