# Supplementary material for: Routemap for health impact assessment implementation: scoping review using the consolidated framework for implementation research
Source: Health Promot Int. 2025 Jun 30;40(3):daaf080. doi: 10.1093/heapro/daaf080 (PMC12208066; doi:10.1093/heapro/daaf080)
Supplement: daaf080_Supplementary_Data [file daaf080_supplementary_data.zip › SM File 5. Summary Table of barriers and facilitators.docx]

| **CFIR domain (application in HIA context)** | **Construct & *definition* as per CFIR* and CRIF2.0**  **(New subconstructs added**)** | **Studies where HIA facilitators were identified and classified** | **Studies where HIA barriers were identified and classified** |
| --- | --- | --- | --- |
| Innovation Characteristics (The ‘thing’ being implemented: HIA as a tool and method) | Innovation source: *The group that developed and/or visibly sponsored use of the innovation is reputable, credible, and/or trustable’* | No data | No data |
|  | Innovation Evidence base: *The innovation has robust evidence supporting its effectiveness* | No data | Kraemer, Nikolajsen, and Gulis 2014; Ramirez-Rubio et al. 2019; Fischer, Chang and Muthoora; 2024  Thondoo et al. 2019; Thondoo et al 2022, Kögel et al. 2020 |
|  | Innovation Relative advantage: *The innovation is [perceived] better than other available innovations or current practice* | Mattig et al. 2017; Thondoo et al. 2020, Damari, Vosoogh-Moghaddam and Riazi-Isfahani 2018; Jabot and Rivadeneyra-Sicilia 2022; Haigh et al. 2013; Jabot et al. 2020; O’Mullane 2014; Liu et al. 2023; Goff et al. 2016 | Damari, Vosoogh-Moghaddam, and Riazi-Isfahani 2018; Ison 2013; Mattig et al. 2017, Quin, Carmichael, and Hopper 2023; Ison 2013; O’Mullane 2014 |
|  | Innovation Adaptability: *The innovation can be modified, tailored, or refined to fit local context or needs* | Jabot et al. 2020, Jabot and Rivadeneyra-Sicilia 2022; Ramirez-Rubio et al. 2019 | Damari, Vosoogh-Moghaddam, and Riazi-Isfahani 2018; Bever et al. 2021;  Roue le-Gall & Jabot 2017 |
|  | Innovation Trialability: *The innovation can be tested or piloted on a small scale and undone* | No data | No data |
|  | Innovation complexity: *The innovation is complicated, which may be reflected by its scope and/or the nature and number of connections and steps* | No data | Busato and Grisotti 2022; Ison, 2013; Kraemer, Nikolajsen, and Gulis 2014; Green et al. 2020; Damari, Vosoogh-Moghaddam, and Riazi-Isfahani 2018 |
|  | Innovation design | No data | No data |
|  | Innovation cost: *operating costs are affordable* | No data | Quin, Carmichael, and Hopper 2023; Mattig et al 2017; Bever et al. 2021 |
| Outer Setting  (the local or national context such as external policies that may influence the working of the organisations involved in implementing HIA) | Critical incidents: Large-scale and/or unanticipated events disrupt implementation and/or delivery of the innovation. | No data | Morteruel et al., 2020 |
|  | Local Attitudes: Sociocultural values (e.g., shared responsibility in helping recipients) and beliefs (e.g., convictions about the worthiness of recipients) encourage the Outer Setting to support implementation and/or delivery of the innovation | No data | No data |
|  | Local Conditions:  *Economic, environmental, political, and/or technological conditions enable the Outer Setting to support implementation and/or delivery of the innovation* | Jabot and Rivadeneyra-Sicilia 2022. Kögel et al., 2020; Marincová, Loosova and Valenta 2020; O’Mullane 2014, Linzalone et al. 2018; Gamache et al. 2020; Liu et al. 2023, Damari et al 2018, Rivadeneyra-Sicilia 2022 | Bourcier et al. 2015; Morteruel et al. 2020; Kraemer, Nikolajsen, and Gulis 2014; O’Mullane 2014; Linzalone et al. 2017; Thondoo et al. 2022; Bever et al. 2021; Marincová, Loosova and Valenta 2020, Thondoo et al. 2022; Ramirez-Rubio et al. 2019 |
|  | Partnerships and Connections: *The Inner Setting is networked with external entities, including referral networks, academic affiliations, and professional organization networks* | Mattig et al. 2017; Gamache et al. 2020; Jabot et al. 2020; Linzalone et al. 2018; Haigh et al. 2015, Thondoo et al. 2020; Walpita and Green 2020; Kraemer, Nikolajsen, and Gulis 2014; O’Mullane 2014; Marincová, Loosova and Valenta 2020; Ison 2013; Morteruel et al. 2020; Jabot & Rivadeneyra-Sicilia 2022; Ramirez-Rubio 2019; Roue le-Gall and Jabot 2017; Bever et al. 2021; Goff et al. 2016, Linzalone et al. 2017 | Ison 2013; Ramirez-Rubio et al. 2019 |
|  | Policies and Laws: *Legislation, regulations, professional group guidelines and recommendations, or accreditation standards support implementation and/or delivery of the innovation.* | Roue le-Gall & Jabot 2017; Ramirez-Rubio 2019 | Damari, Vosoogh-Moghaddam, and Riazi-Isfahani 2018; Quin, Carmichael, and Hopper 2023; Ison 2013 Morteruel et al. 2020; Thondoo et al 2022 |
|  | Financing: *Funding from external entities (e.g., grants, reimbursement) is available to implement and/or deliver the innovation* | Potential overlap with funding in inner | Potential overlap with funding in inner |
|  | External Pressure: *External pressures drive implementation and/or delivery of the innovation* | Jabot & Rivadeneyra-Sicilia 2022 | Mattig et al. 2017; O’Mullane 2014 |
| Inner Setting (the organisational context of those involved in implementing the HIA) | Structural Characteristics  *Infrastructure components support functional performance of the Inner Setting* | Linzalone et al. 2018 | Jabot et al. 2020; Kraemer, Nikolajsen, and Gulis 2014; Morteruel et al. 2020 |
|  | Relational Connections: *There are high quality formal and informal relationships, networks, and teams within and across Inner Setting boundaries (e.g., structural, professional).* | Potential overlap with Partnerships and Connections | Potential overlap with Partnerships and Connections |
|  | Communications: *There are high quality formal and informal information sharing practices within and across Inner Setting boundaries (e.g., structural, professional).* | Potential overlap with Partnerships and Connections | Potential overlap with Partnerships and Connections |
|  | Culture: *There are shared values, beliefs, and norms across the Inner Setting* | No Data | Ison, 2013; Kraemer, Nikolajsen, and Gulis 2014; Morteruel et al. 2020 |
|  | Tension for Change:  *The current situation is intolerable and needs to change* | No Data | No Data |
|  | Compatibility: *The innovation fits with workflows, systems, and processes*. | No Data | Jambot et al. 2020; Morteruel et al. 2020 |
|  | Relative Priority: Implementing and delivering the innovation is important compared to other initiatives. | No Data | Kraemer, Nikolajsen, and Gulis 2014 |
|  | Incentive Systems: *Tangible and/or intangible incentives and rewards and/or disincentives and punishments support implementation and delivery of the innovation.* | No Data | Kraemer, Nikolajsen, and Gulis 2014 |
|  | Mission Alignment: *Implementing and delivering the innovation is in line with the overarching commitment, purpose, or goals in the Inner Setting.* | No Data | Kraemer, Nikolajsen, and Gulis 2014 |
|  | Available resources: *Resources are available to implement and deliver the innovation* (ie. trained staff, funding and access to knowledge and information such as guidance and training and sufficient time **) | Goff et al. 2016; Ison 2013;  Linzalone et al. 2018; Fakhri, Harris, and Maleki 2015; Morteruel et al. 2020; Gamache et al. 2020; Thondoo et al. 2020; Linzalone et al. 2018, O’Mullane 2014;Walpita & Green 2020, Jabot et al. 2020; Kraemer, Nikolajsen, and Gulis 2014; Quin, Carmichael, and Hopper 2023;Marincová, Loosova and Valenta 2020; Quin, Carmichael, and Hopper 2023; Purcell and Kearns 2013, Jabot and Rivadeneyra-Sicilia 2022; Goff et al. 2016; Sheffield et al. 2014 | Ison 2013; Jabot and Rivadeneyra -Sicilia 2022; Negev et al. 2013; Buregeya, Loignon, and Brousselle 2020; Fakhri and Harris 2021; Marincová, Loosova and Valenta 2020; Morteruel et al. 2020, O’ Mullane 2014, Thondoo et al 2020; Kögel et al. 2020; Jambot et al. 2020, Gamache et al. 2020, Harris-Roxas et al. 2014, Haigh et al, 2015; Damari, Vosoogh-Moghaddam and Riazi-Isfahani 2018; Bourcier et al. 2015; Fakhri, Harris, and Maleki 2015; Kraemer, Nikolajsen, and Gulis 2014; Linzalone et al. 2018; Thondoo et al 2020; Kraemer, Nikolajsen, and Gulis 2014; Quin, Carmichael, and Hopper 2023; Morteruel et al. 2020; Goff et al. 2016; Liu et al. 2023 |
| Individuals Domain*: Perceptions those involved in HIA (deliverers & recipients) and roles and characteristics | Knowledge and Belief about HIA*: *Individuals’ attitudes toward and value placed on the innovation, as well as familiarity with facts, truths, and principles related to the innovation* | No Data | Harris-Roxas et al. 2014; Liu et al. 2023; Mattig et al. 2017; Fakhri, Harris, and Maleki 2015; Gamache et al. 2020; Ison 2013; Damari, Vosoogh-Moghaddam, and Riazi-Isfahani 2018; Marincová, Loosova and Valenta 2020; Kraemer, Nikolajsen, and Gulis 2014; O’Mullane, 2014; Negev et al., 2013 |
|  | Self-efficacy*: *Individual belief in their own capabilities to execute courses of action to achieve implementation goals* (added Agency** and feelings of ‘tokenism’**) | No Data | Jabot et al. 2020, Gamache et al. 2020, Ison 2013; Harris-Roxas et al. 2014, O’Mullane, 2014 |
|  | Project Roles: *The roles and characteristics of individuals* (including high-level leaders, implementation facilitators/leads/team members) | Bourcier et al. 2015; Harris-Roxas et al. 2014; Ison 2013; Jabot et al 2020; Gamache et al. 2020; Haigh et al. 2015, Kraemer, Nikolajsen, and Gulis 2014; Linzalone et al. 2017; Gamache et al. 2020; Bourcier et al 2015; Busato and Grisotti 2022; Morteruel et al 2020; Green et al. 2020; Sheffield et al. 2014; Kögel et al. 2020; Negev et al. 2020 | Negev et al. 2013 |
| **Process**  The activities and strategies used to implement HIA | Teaming:  *Join together, intentionally coordinating and collaborating on interdependent tasks, to implement the innovation* | No Data | No Data |
|  | Assessing Needs  *Collect information about priorities, preferences, and needs of people involved (deliverers and recipients)* | Haigh et al. 2015; Linzalone et al. 2018; Busato and Grisotti 2020; Bourcier et al 2015; Purcell and Kearns 2013; Negev et al. 2013; Kögel et al. 2020 | Purcell and Kearns 2013 |
|  | Assessing context: *Collect information to identify and appraise barriers and facilitators to implementation and delivery of the innovation* | Damari, Vosoogh-Moghaddam, and Riazi-Isfahani 2018; Fakhri and Harris 2021; Haigh et al. 2015; Kraemer, Nikolajsen, and Gulis 2014; Gamache et al. 2020; Pradyumna et al., 2021; Bourcier et al. 2015; Morteruel et al. 2020 | No Data |
|  | Planning: *Identify roles and responsibilities, outline specific steps and milestones, and define goals and measures for implementation success in advance.* | Haigh et al. 2015; Jabot et al. 2020; Kraemer, Nikolajsen, and Gulis 2014; Busato and Grisotti 2020; Gamache et al. 2020Goff et al. 2016; Roue le-Gall & Jabot 2017; Ramirez-Rubio et al 2019; Negev et al. 2013 | No Data |
|  | Tailoring Strategies: *Choose and operationalize implementation strategies to address barriers, leverage facilitators, and fit context* | Morteruel et al 2020, Thondoo et al 2020; Goff et al., 2016 | No Data |
|  | Engaging (all stakeholders)  Attract and encourage participation in implementation and/or the innovation including issues of trust | Morteruel et al. 2020; Bourcier et al. 2015; Gamache, Diallo, and Lebel 2022; Buregeya, Loignon, and Brousselle 2020; Haigh et al. 2015; Thondoo et al. 2020; Goff et al. 2016; Roue le-Gall and Jabot 2017; Negev et al. 2013, Linzalone et al. 2017; Morteruel et al. 2020, Purcell and Kearns 2013; Fischer, Chang and Muthoora 2024. | Damari, Vosoogh-Moghaddam, and Riazi-Isfahani 2018; Fakhri, Harris, and Maleki 2015; Harris-Roxas et al. 2014; Ramirez-Rubio et al 2019; Purcell & Kearns 2013; Bourcier et al. 2015, |
|  | Doing: *Implement in small steps, tests, or cycles of change to trial and cumulatively optimize delivery of the innovation.*  (steps to improve transparency** included in this construct) | Bourcier et al. 2015; Busato and Grisotti, 2020; Morteruel et al. 2020; Fakhri, Harris, and Maleki 2015; Thondoo et al 2020; Marincová, Loosova and Valenta 2020; Thondoo et al. 2020a; Morteruel et al. 2020; Gamache, Diallo and Lebel et al. 2022; Linzalone et al. 2017; Berensson & Tillgren 2017; Bourcier et al. 2015; Goff et al., 2016; Ramirez-rubio et al. 2019; Westenhöfer et al. 2023; Jabot and Rivadeneyra-Sicilia 2022; Negev et al., 2013; Sheffield et al. 2014; Marincová, Loosova and Valenta 2020; Liu et al. 2023; Fischer, Chang and Muthoora 2024. | Ramirez-Rubio et al 2019; Linzalone et al. 2017; Del Rio et al. 2017 |
|  | Reflecting and evaluating: *Collect and discuss quantitative and qualitative information about the success of implementation* (including evaluating indirect benefits) | Bever et al 2021; Green et al. 2020; Haigh et al. 2013; Liu et al. 2023; Fischer, Chang and Muthoora 2024. | Bourcier et al. 2015, Damari, Vosoogh-Moghaddam, and Riazi-Isfahani 2018 |
|  | Adapting: *Modify the innovation and/or the Inner Setting for optimal fit and integration into work processes*. | Haigh et al. 2015; Jabot et al. 2020; Gamache et al. 2020; Negev et al. 2013 | Jabot et al. 2020, Morteruel et al. 2020 |

**References**

Berensson, K., & Tillgren, P. (2017). Health impact assessment (HIA) of political proposals at the local level: Successful introduction, but what has happened 15 years later? *Global Health Promotion*, *24*(2), 43–51. https://doi.org/10.1177/1757975916683386

Bever, E., Arnold, K. T., Lindberg, R., Dannenberg, A. L., Morley, R., Breysse, J., & Pollack Porter, K. M. (2021). Use of health impact assessments in the housing sector to promote health in the United States, 2002–2016. *Journal of Housing and the Built Environment*, *36*(3), 1277–1297. https://doi.org/10.1007/s10901-020-09795-9

Bourcier, E., Charbonneau, D., Cahill, C., & Dannenberg, A. L. (2015). An Evaluation of Health Impact Assessments in the United States, 2011–2014. *Preventing Chronic Disease*, *12*, 140376. https://doi.org/10.5888/pcd12.140376

Buregeya, J. M., Loignon, C., & Brousselle, A. (2020). Contribution analysis to analyze the effects of the health impact assessment at the local level: A case of urban revitalization. *Evaluation and Program Planning*, *79*, 101746–15. https://doi.org/10.1016/j.evalprogplan.2019.101746

Busato, M. A., & Grisotti, M. (2022). Health impact assessment in the process of implementation of hydroelectric plants: Methodological contributions. *Ambiente & Sociedade*, *25*(Journal Article). https://doi.org/10.1590/1809-4422asoc20200068r1vu2022l3oa

Damari, B., Vosoogh-Moghaddam, A., & Riazi-Isfahani, S. (2018). Implementing health impact assessment at national level: An experience in Iran. *Iranian Journal of Public Health*, *47*(2), 246–255. https://go.exlibris.link/XVYpPW91

Del Rio, M., Hargrove, W. L., Tomaka, J., & Korc, M. (2017). Transportation Matters: A Health Impact Assessment in Rural New Mexico. *International Journal of Environmental Research and Public Health*, *14*(6). https://doi.org/10.3390/ijerph14060629

Fakhri, A., & Harris, P. (2021). Internationally validating a conceptual framework for health impact assessment. *International Archives of Health Sciences*, *8*(4), 231–236. https://doi.org/10.4103/iahs.iahs_42_21

Fakhri, A., Harris, P., & Maleki, M. (2015). Proposing a framework for Health Impact Assessment in Iran. *BMC Public Health*, *15*(1), 1–7. https://doi.org/10.1186/s12889-015-1698-1

Fischer, T. B., Chang, M., & Muthoora, T. (2024). Health impact assessment in two planning projects in England: Reflections on normative effectiveness. *BMC Public Health*, *24*(1), 2819. https://doi.org/10.1186/s12889-024-20203-7

Gamache, S., Diallo, T., & Lebel, A. (2022). The use of health impact assessments performed in Quebec City (Canada) – 2013–2019: Stakeholders and participants’ appreciation. *Environmental Impact Assessment Review*, *92*(Journal Article), 106693. https://doi.org/10.1016/j.eiar.2021.106693

Gamache, S., Lebel, A., Diallo, T. A., & Shankardass, K. (2020). The elaboration of an intersectoral partnership to perform health impact assessment in urban planning: The experience of quebec city (canada). *International Journal of Environmental Research and Public Health*, *17*(20), 1–15. https://doi.org/10.3390/ijerph17207556

Goff, N., Wyss, K., Wendel, A., & Jarris, P. (2016). Implementing Health Impact Assessment Programs in State Health Agencies: Lessons Learned From Pilot Programs, 2009-2011. *Journal of Public Health Management and Practice : JPHMP*, *22*(6), E8–E13. https://doi.org/10.1097/PHH.0000000000000392

Green, L., Ashton, K., Edmonds, N., & Azam, S. (2020). Process, Practice and Progress: A Case Study of the Health Impact Assessment (HIA) of Brexit in Wales. *International Journal of Environmental Research and Public Health*, *17*(18), 1–14. https://doi.org/10.3390/ijerph17186652

Green, L., Gray, B. J., & Ashton, K. (2020). Using health impact assessments to implement the sustainable development goals in practice: A case study in Wales. *Impact Assessment and Project Appraisal*, *38*(3), 214–224. https://doi.org/10.1080/14615517.2019.1678968

Haigh, F., Baum, F., Dannenberg, A. L., Harris, M. F., Harris-Roxas, B., Keleher, H., Kemp, L., Morgan, R., Chok, H. N., Spickett, J., & Harris, E. (2013). The effectiveness of health impact assessment in influencing decision-making in Australia and New Zealand 2005-2009. *BMC Public Health*, *13*(1), 1188–1188. https://doi.org/10.1186/1471-2458-13-1188

Haigh, F., Harris, E., Harris-Roxas, B., Baum, F., Dannenberg, A. L., Harris, M. F., Keleher, H., Kemp, L., Morgan, R., Chok, H. N. G., & Spickett, J. (2015). What makes health impact assessments successful? Factors contributing to effectiveness in Australia and New Zealand. *BMC Public Health*, *15*(1), 1009–1009. https://doi.org/10.1186/s12889-015-2319-8

Harris-Roxas, B., Haigh, F., Travaglia, J., & Kemp, L. (2014). Evaluating the impact of equity focused health impact assessment on health service planning: Three case studies. *BMC Health Services Research*, *14*(1), 371–371. https://doi.org/10.1186/1472-6963-14-371

Ison, E. (2013). Health Impact Assessment in a Network of European Cities. *Journal of Urban Health*, *90*(Suppl 1), 105–115. https://doi.org/10.1007/s11524-011-9644-8

Jabot, F., & Rivadeneyra-Sicilia, A. (2022). Health impact assessment institutionalisation in France: State of the art, challenges and perspectives. *IMPACT ASSESSMENT AND PROJECT APPRAISAL*, *40*(3), 179–190. https://doi.org/10.1080/14615517.2021.2012011

Jabot, F., Tremblay, E., Rivadeneyra, A., Diallo, T. A., & Lapointe, G. (2020). A comparative analysis of health impact assessment implementation models in the regions of montérégie (Québec, canada) and nouvelle-aquitaine (france). *International Journal of Environmental Research and Public Health*, *17*(18), 1–18. Scopus. https://doi.org/10.3390/ijerph17186558

Kögel, C. C., Peña, T. R., Sánchez, I., Tobella, M., López, J. A., Espot, F. G., Claramunt, F. P., Rabal, G., & Viana, A. G. (2020). Health impact assessment (HIA) of a fluvial environment recovery project in a medium-sized Spanish Town. *International Journal of Environmental Research and Public Health*, *17*(5), 1484. https://doi.org/10.3390/ijerph17051484

Kraemer, S. & Gulis, G. (2014). How do experts define relevance criteria when initiating Health Impact Assessments of national policies? *Scandinavian Journal of Public Health*, *42*(1), 18–24. https://doi.org/10.1177/1403494813504254

Kræmer, S. Johnsdatter, R., Theilgaard Nikolajsen, L and Gulis, G. (2014). Implementation of health impact assessment in Danish municipal context. *Central European Journal of Public Health*, *22*(4), Article 4. https://doi.org/10.21101/cejph.a3943

Linzalone, N., Ballarini, A., Piccinelli, C., Viliani, F., & Bianchi, F. (2018). Institutionalizing Health Impact Assessment: A consultation with experts on the barriers and facilitators to implementing HIA in Italy. *Journal of Environmental Management*, *218*, 95–102. https://doi.org/10.1016/j.jenvman.2018.04.037

Linzalone, N., Coi, A., Lauriola, P., Luise, D., Pedone, A., Romizi, R., Sallese, D., Bianchi, F., Santoro, M., Minichilli, F., Maurello, M. T., Scaringi, M., Zuppiroli, M. E., HIA21 Project Working Group, & HIA21 Project Working Grp. (2017). Participatory health impact assessment used to support decision-making in waste management planning: A replicable experience from Italy. *Waste Management (Elmsford)*, *59*(Journal Article), 557–566. https://doi.org/10.1016/j.wasman.2016.09.035

Liu, X., Liu, Y., Xu, Y., Song, L., Huang, Z., Zhu, X., & Zhang, M. (2023). Current status and influencing factors of policy identification in health impact assessment: A case study of Zhejiang Province. *Health Research Policy and Systems*, *21*(1), 118. https://doi.org/10.1186/s12961-023-01064-9

Marincova, L., Loosova, J., & Valenta, V. (2020). Experiences and needs of Licences Health Risk Assessors using Health Impact Assessment in the Czech Republic. *Central European Journal of Public Health*, *28*(2), 108–113. https://doi.org/10.21101/cejph.a5833

Mattig, T., Cantoreggi, N., Simos, J., Kruit, C. F., & Christie, D. P. T. H. (2017). HIA in Switzerland: Strategies for achieving Health in All Policies. *Health Promotion International*, *32*(1), 149–156. https://doi.org/10.1093/heapro/dav087

Morteruel, M., Bacigalupe, A., Aldasoro, E., Larrañaga, I., & Serrano, E. (2020). Health impact assessments in Spain: Have they been effective? *International Journal of Environmental Research and Public Health*, *17*(8), Article 8. https://doi.org/10.3390/ijerph17082959

Movia, M., Macher, S., Antony, G., Zeuschner, V., Wamprechtsamer, G., Delle Grazie, J., Simi, H., & Fuchs-Neuhold, B. (2022). Health Impact Assessment (HIA) of a Daily Physical Activity Unit in Schools: Focus on Children and Adolescents in Austria Up to the 8th Grade. *International Journal of Environmental Research and Public Health*, *19*(11), Article 11. https://doi.org/10.3390/ijerph19116428

Negev, M., Davidovitch, N., Garb, Y., & Tal, A. (2013). Stakeholder participation in health impact assessment: A multicultural approach. *Environmental Impact Assessment Review*, *43*(Journal Article), 112–120. https://doi.org/10.1016/j.eiar.2013.06.002

O’Mullane, M. (2014). Implementing the legal provisions for HIA in Slovakia: An exploration of practitioner perspectives. *Health Policy*, *117*(1), 112–119. Scopus. https://doi.org/10.1016/j.healthpol.2014.03.005

Pradyumna, A., Farnham, A., Utzinger, J., & Winkler, M. S. (2021). Health impact assessment of a watershed development project in southern India: A case study. *IMPACT ASSESSMENT AND PROJECT APPRAISAL*, *39*(2), 118–126. https://doi.org/10.1080/14615517.2020.1863119

Pursell, L., & Kearns, N. (2013). Impacts of an HIA on inter-agency and inter-sectoral partnerships and community participation: Lessons from a local level HIA in the Republic of Ireland. *Health Promotion International*, *28*(4), 522–532. https://doi.org/10.1093/heapro/das032

Quin, M., Carmichael, L., & Hopper, C. (2023). Implementing Health Impact Assessment policy on infrastructure development in the London Borough of Tower Hamlets. *Cities and Health*, *7*(3), 303–311. Scopus. https://doi.org/10.1080/23748834.2022.2148843

Ramirez-Rubio, O., Daher, C., Fanjul, G., Gascon, M., Mueller, N., Pajin, L., Plasencia, A., Rojas-Rueda, D., Thondoo, M., & Nieuwenhuijsen, M. J. (2019). Urban health: An example of a ‘health in all policies’ approach in the context of SDGs implementation. *Globalization and Health*, *15*(1), 87–87. https://doi.org/10.1186/s12992-019-0529-z

Roué-Le Gall, A., & Jabot, F. (2017). Health impact assessment on urban development projects in France: Finding pathways to fit practice to context. *Global Health Promotion*, *24*(2), 25–34. https://doi.org/10.1177/1757975916675577

Sheffield, P., Rowe, M., Agu, D., Rodríguez, L., & Avilés, K. (2014). Health Impact Assessments for Environmental Restoration: The Case of Caño Martín Peña. *Annals of Global Health*, *80*(4), 296–302. https://doi.org/10.1016/j.aogh.2014.07.001

Thondoo, M., De Vries, D. H., Rojas-Rueda, D., Ramkalam, Y. D., Verlinghieri, E., Gupta, J., & Nieuwenhuijsen, M. J. (2020). Framework for Participatory Quantitative Health Impact Assessment in Low- and Middle-Income Countries. *International Journal of Environmental Research and Public Health*, *17*(20), 1–20. https://doi.org/10.3390/ijerph17207688

Thondoo, M., Goel, R., Tatah, L., Naraynen, N., Woodcock, J., & Nieuwenhuijsen, M. (2022). The Built Environment and Health in Low- and Middle-Income Countries: A Review on Quantitative Health Impact Assessments. *Current Environmental Health Reports*, *9*(1), 90–103. https://doi.org/10.1007/s40572-021-00324-6

Thondoo, M., Mueller, N., Rojas-Rueda, D., de Vries, D., Gupta, J., & Nieuwenhuijsen, M. J. (2020a). Participatory quantitative health impact assessment of urban transport planning: A case study from Eastern Africa. *Environment International*, *144*, 106027. https://doi.org/10.1016/j.envint.2020.106027

Thondoo, M., Rojas-Rueda, D., Gupta, J., de Vries, D. H., & Nieuwenhuijsen, M. J. (2019). Systematic Literature Review of Health Impact Assessments in Low and Middle-Income Countries. *International Journal of Environmental Research and Public Health*, *16*(11), 2018. https://doi.org/10.3390/ijerph16112018

Walpita, Y. N., & Green, L. (2022). Health Impact Assessment (HIA): A Comparative Case Study of Sri Lanka and Wales: What Can a Developing Country Learn From the Welsh HIA System? *International Journal of Health Services*, *52*(2), 283–291. https://doi.org/10.1177/0020731420941454

Westenhöfer, J., Nouri, E., Reschke, M. L., Seebach, F., & Buchcik, J. (2023). Walkability and urban built environments-a systematic review of health impact assessments (HIA). *BMC Public Health*, *23*(1), 518–518. https://doi.org/10.1186/s12889-023-15394-4
